# Scale-Up of Continuous Metallaphotoredox Catalyzed C–O Coupling to a 10 kg-Scale Using Small Footprint Photochemical Taylor Vortex Flow Reactors

**DOI:** 10.1021/acs.oprd.4c00262

**Published:** 2024-12-04

**Authors:** Rodolfo I. Teixeira, Toby H. Waldron Clarke, Ashley Love, Xue-Zhong Sun, Surajit Kayal, Michael W. George

**Affiliations:** School of Chemistry, The University of Nottingham, University Park, Nottingham NG7 2RD, U.K.

**Keywords:** scale-up, flow photochemistry, Taylor vortex reactor, photocatalysis, metallaphotoredox, ultrafast spectroscopy

## Abstract

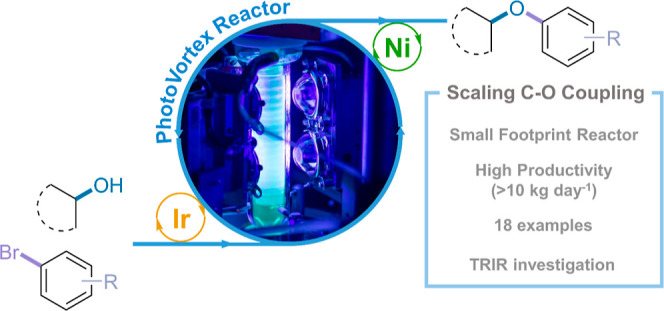

We report the development and optimization of a scalable flow process for metallaphotoredox (Ir/Ni) C–O coupling, a mild and efficient approach for forming alkyl-aryl ethers, a common motif in medicinal and process chemistry settings. Time-resolved infrared spectroscopy (TRIR) highlighted the amine as the major quencher of the photocatalyst triplet excited state, along with the formation of an Ir(II) species that, in the presence of the Ni cocatalyst, has its lifetime shortened, suggesting reductive quenching of Ir(III)*, followed by reoxidation facilitated by the Ni cocatalyst. TRIR and batch reaction screening was used to develop conditions transferrable to flow, and many processing benefits of performing the reaction in flow were then demonstrated using a simple to construct/operate, small-footprint FEP coil flow reactor, including short (<10 min) space times and reduced catalyst loadings (down to 0.1 mol % Ir, 1 mol % Ni) while retaining good yield/conversion. Scalability was demonstrated by increasing the length or diameter of the FEP coil flow reactor tubing, however, due to suspected mass transfer/mixing limitations, the yield decreased upon scale-up in some cases. Therefore, we applied a modified version of our previously reported photochemical Taylor Vortex Flow Reactor (PhotoVortex), where Taylor vortices and a short-irradiated path length allow photochemical reactions to be performed efficiently via excellent mixing. In a small PhotoVortex (8 mL irradiated volume), we have demonstrated projected productivities around 1 kg day^–1^ and >10 kg day^–1^ in a large PhotoVortex (185 mL irradiated volume) with good product yields (>90%) and low catalyst loadings (0.1 to 0.5 mol % of [Ir{dF(CF_3_)ppy}_2_dtbbpy]PF_6_), enabled by excellent mixing ensuring sufficient mass transfer between short-lived photoexcited and other transient species.

## Introduction

**Figure 1:**
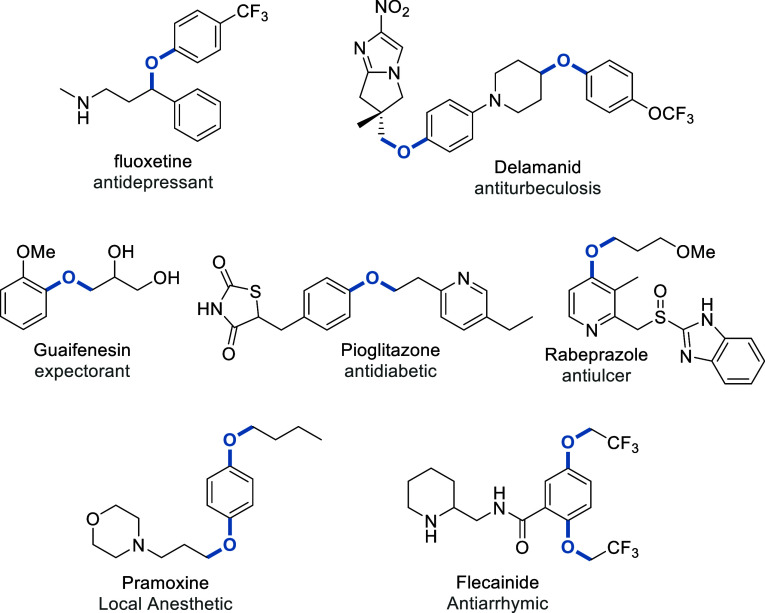
Structure of selected pharmaceuticals containing the alkyl-aryl ether motif.

Alkyl-aryl ethers (R–O–Ar) are common structural motifs found in many natural products and commercial chemicals,^1−3^ including top-selling drugs and some active pharmaceuticals, such as Fluoxetine and Delamanid (see Figure 1), listed on the WHO essential medicines list.^4^ Highly productive routes and mild conditions for the preparation of these compounds are desirable, and a variety of synthetic methodologies have been developed.^5^

Traditional synthetic approaches for the preparation of alkyl-aryl ethers include nucleophilic aromatic substitution, Mitsunobu reaction, Ullmann coupling and Buchwald–Hartwig coupling reactions.^2,5−7^ These approaches often present drawbacks, such as S_N_Ar reactions requiring highly activated aryl halides, stoichiometric phosphine oxide waste associated with Mitsunobu reactions and difficult to predict reaction outcomes highly dependent on the nature of the substrates, solvents, bases, and catalyst employed for metal-catalyzed couplings.^5,8,9^

Photoredox catalysis is a useful tool for initiating electron transfer processes under mild reaction conditions and has been widely applied to various organic transformations.^10,11^ A seminal report by MacMillan and co-workers demonstrated that an Ir photocatalyst, in tandem with a Ni cocatalyst (dual metallaphotoredox catalysis), could promptly accomplish C–O coupling reactions between aryl bromides and primary/secondary alcohols to give alkyl-aryl ethers, under mild conditions with good functional group tolerance.^12^ Further studies using this approach have been widespread, including the preparation of new scaffolds, including deuterated adducts, glycosides, and pharmaceuticals, highlighting the scope and utility of this methodology.^13−15^

Transient UV/visible absorption (TA) spectroscopy has previously been used to identify possible intermediates involved in photochemically induced ultrafast dynamic processes.^16−18^ Time-resolved infrared spectroscopy (TRIR) can also be particularly useful for probing the nature and reactivity of the excited states and the elucidation of subsequent mechanisms.^19−22^ For example, we have recently demonstrated that such measurements were key for the development of the rational design of triplet sensitizers for the execution of visible light photochemistry^23^ as well as for the integration of multistep photochemical and thermal continuous flow reactions producing bicyclic lactones with kilogram productivity.^24^

**Figure 2:**
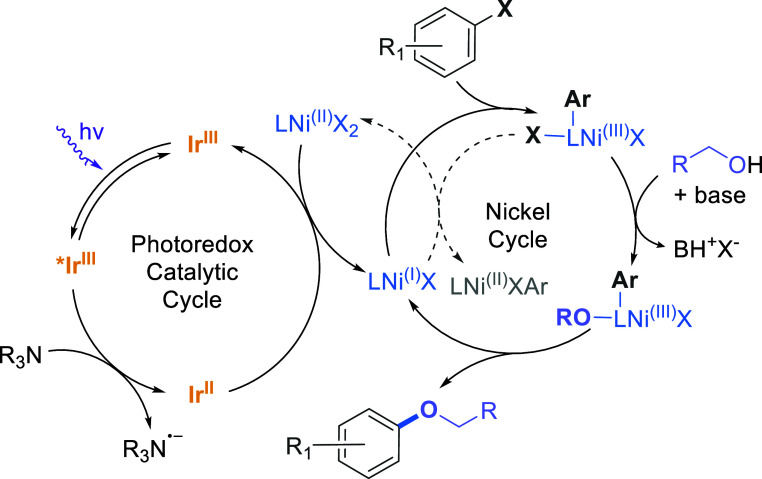
Simplified mechanism adapted from refs (10) and (25) proposed for the metallaphotoredox C–O coupling between aryl halides and alcohols. Dashed arrows show competitive pathways with main catalytic cycle.

The mechanism of the dual Ir/Ni metallaphotoredox C–O coupling reaction (and related reactions) has previously been investigated using TA spectroscopy.^25^ These investigations have shown that the amine employed (quinuclidine) was the predominant quencher of the excited state Ir(III)*, generating the Ir(II) species through electron transfer, followed by activation of the Ni(II) chloride precatalyst to the Ni(I) species via reduction by the Ir(II) photocatalyst. It has also been reported that the Ni catalytic cycle might be self-sustained;^25,26^ not every cycle involving the Ni catalyst may require a photon for turnover (quantum yield >1). However, continuous irradiation is necessary for efficient reactions, possibly due to the formation of inactive, off-cycle Ni species.^25,27,28^ These studies have allowed the development of Ni coupling protocols, highlighting the importance of mechanistic studies for reaction design and optimization for batch processes.^29^ Additionally, a theoretical investigation suggests that the amine can be involved in a hydrogen atom transfer (HAT) process with the alcohol coupling partner,^30^ and mechanistic studies suggest that chlorine radicals generated due to Ni cocatalyst photochemistry should also be considered in the systems.^31^ In this paper, we use mechanistic insights to aid reaction optimization together with new reactor developments to carry out metallaphotoredox reactions as continuous processes at scale. A simplified scheme of the reaction mechanism is shown in Figure 2.

Continuous flow processing offers benefits for photochemical reactions and a simplified means of scalability, which is important for chemical manufacturing/production.^32,33^ These benefits potentially include better light penetration, improved mixing, shorter processing times, greater energy efficiency, reduction in catalyst loadings, among others. Therefore, an increasing effort, including by our group,^34−38^ has been made to develop flow reactors and continuous methodologies for photochemical reactions relevant to chemical processing.^39^ Of particular relevance to this paper is our continuous Taylor Vortex Flow Reactor, which we have used for the scale-up of both photochemical^34,35,40^ and electrochemical processes.^41−43^ This reactor has recently attracted interest from others.^44,45^

Recent efforts to scale-up metallaphotoredox C–O coupling reactions have been made and they have been demonstrated on a multigram scale in batch.^46^ However, attempts to scale-up this reaction in flow have been unsuccessful so far due to insolubility of the base.^46,47^ Here, we present the development and optimization of a robust and scalable continuous flow approach for synthesizing alkyl-aryl ethers using dual Ir/Ni metallaphotoredox catalysis in our continuous Photochemical Taylor Vortex Flow Reactors to multikilogram scale together with TRIR mechanistic investigations to unravel reaction parameters.

## Results and Discussion

### Initial Mechanistic Considerations Using TRIR Spectroscopy

In the original paper by MacMillan and co-workers,^12^ they reported the coupling of 4-bromoacetophenone and 1-hexanol, using [Ir{dF(CF_3_)ppy}_2_dtbbpy]PF_6_ as the photocatalyst, NiCl_2_-dtbbpy as cocatalyst and quinuclidine as the electron donor in the presence of K_2_CO_3_. Our aim is to convert this process to continuous flow for easier scale-up and increased productivity. Therefore, the presence of solid reagents or byproducts is problematic, as they can cause unintended blockages. Nocera and co-workers have reported that the reaction can proceed using stoichiometric amounts of quinuclidine as a base and electron donor.^25^ To gain insights into the mechanism, we investigated this reaction using both ultrafast TA and TRIR spectroscopy by studying the behavior of [Ir{dF(CF_3_)ppy}_2_dtbbpy]PF_6_ photocatalyst along with the reaction components, which were added in different combinations to study the effects.

**Figure 3:**
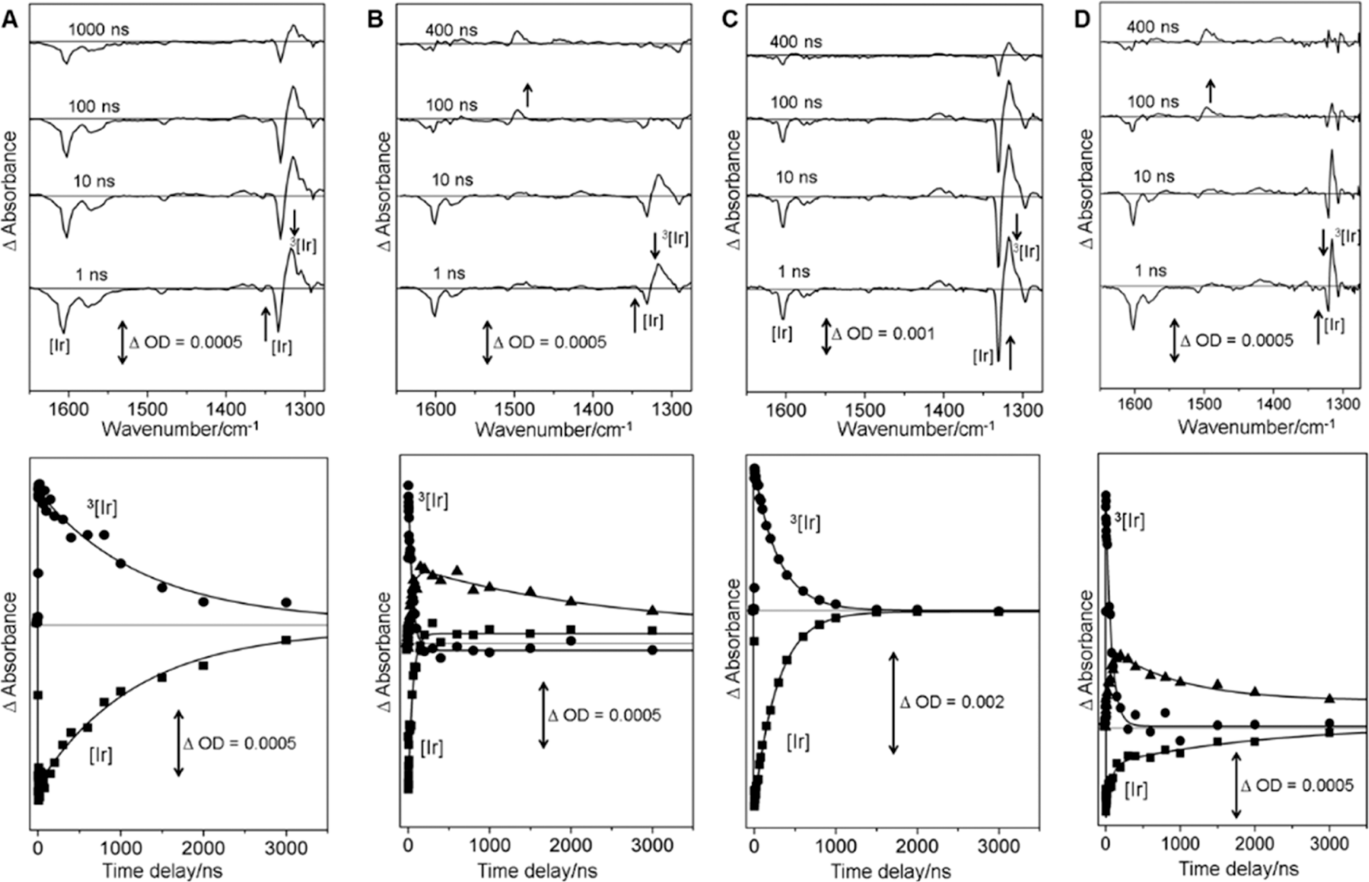
Selected ns-TRIR spectra showing the ^3^MLCT excited state of [Ir{dF(CF_3_)ppy}_2_dtbbpy]PF_6_ (1 mM) (A) and its behavior in the presence of different reaction components following 355 nm excitation. (B) TRIR spectra in the presence of 10 mM quinuclidine showing quenching of the ^3^MLCT excited state of [Ir{dF(CF_3_)ppy}_2_dtbbpy]PF_6_ and formation of [Ir{dF(CF_3_)ppy}_2_dtbbpy]^.-^. (C) TRIR spectra in the presence of 5 mM NiCl_2_-dtbbpy showing quenching of the ^3^MLCT excited state of [Ir{dF(CF_3_)ppy}_2_dtbbpy]PF_6_ and no new detectable transient. (D) TRIR spectra in the presence of the reaction mixture (10 mM 4-bromoacetophenone, 10 mM quinuclidine, 10 mM 1-hexanol, 5 mM NiCl_2_-dtbbpy) showing that with all reaction components, the quinuclidine radical cation is formed (note that the TRIR spectra at different time delays are offset for clarity).

The TA and TRIR spectra shown in Figure 3 and ESI demonstrate the formation of the triplet state of [Ir{dF(CF_3_)ppy}_2_dtbbpy]PF_6_ photocatalyst and its behavior in the presence of different reaction components. The TA obtained 1 ns following excitation (355 nm) of the [Ir{dF(CF_3_)ppy}_2_dtbbpy]PF_6_ in CD_3_CN clearly shows a transient feature at ca. 475 nm, which decays (τ = 0.9 ± 0.1 μs) and similar to one that was previously assigned to the formation of the triplet metal-to-ligand charge transfer (^3^MLCT) of [Ir{dF(CF_3_)ppy}_2_dtbbpy]PF_6_.^25^ The corresponding TRIR spectrum obtained 1 ns after excitation shows the bleach of parent absorptions (1333, 1575, and 1605 cm^–1^) and the production of a relatively strong transient absorption signal at 1316 cm^–1^ together with some weaker bands, Figure 3A. The band at 1316 cm^–1^ decays at the same rate as the parent bands are reformed (τ = 1.1 ± 0.1 μs) and it is also assigned to the ^3^MLCT excited state of the photocatalyst.

Repeating the TA and TRIR experiments in the presence of NiCl_2_-dtbbpy (5 mM) showed similar results except that the decay of the ^3^MLCT excited state and the reformation of [Ir{dF(CF_3_)ppy}_2_dtbbpy]PF_6_ ground state occurred at a faster rate. In addition, in the TA a new transient around 400 nm is rapidly formed (τ = 30 ± 5 ns), consistent with the quenching of the excited state by NiCl_2_-dtbbpy, with a quenching rate constant of *k*_q_ ∼ 1.4 ± 0.1 × 10^8^ L mol^–1^ s^–1^. The band at 400 nm can be assigned to the formation of NiCl_2_-dtbbpy excited state by comparison with previous experiments on similar compounds.^48^ Similar TRIR experiments show that the excited Ir lifetime was also shortened (τ_TRIR_ = 190 ± 15 ns). However, no new transients were observed (Figure 3B).

We next examined the decay of the ^3^MLCT excited state of [Ir{dF(CF_3_)ppy}_2_dtbbpy]PF_6_ 1 mM in the presence of quinuclidine (10 mM). The TA spectra obtained following excitation (355 nm) in the presence of quinuclidine initially show the transient band at 475 nm due to the ^3^MLCT excited state of Ir (see ESI). However, in these experiments, the ^3^MLCT decays (τ = 50 ± 5 ns) to form a new transient feature at ca. 525 nm, which subsequently decays at longer times (τ = 850 ± 100 ns). This new transient was assigned as the reduced [Ir{dF(CF_3_)ppy}_2_dtbbpy]^.-^, and it matches the previously reported absorption of the reduced Ir by spectroelectrochemistry.^25^ Similarly, the TRIR spectrum obtained 1 ns after photolysis shows the presence of the 1316 cm^–1^ band of the ^3^MLCT excited state, and the lifetime of the signal at 1316 cm^–1^ was reduced (τ = 40 ± 5 ns). A new transient absorption at 1486 cm^–1^ grew at the same rate (τ = 40 ± 5 ns) and decayed with a lifetime of (τ = 800 ± 100 ns). The parent band at 1605 cm^–1^ recovers via a biexponential process (*t*_1_ = 50 ± 5 ns and *t*_2_ = 700 ± 100 ns). New bands grow in 1569 and 1275 cm^–1^ at a similar rate as the excited state is quenched (τ = 30 ± 15 ns) and subsequently decays (τ = 700 ± 150 ns) at a similar rate as the parent reforms. These new bands are tentatively assigned to be due to the formation of [Ir{dF(CF_3_)ppy}_2_dtbbpy]^.-^. We obtained the quenching rate constants and found them to be around 20 times higher for quinuclidine (*k*_q_ ∼ 8.2 ± 0.5 × 10^9^ L mol^–1^ s^–1^) than for the NiCl_2_-dtbbpy (*k*_q_ ∼ 1.4 ± 0.1 × 10^8^ L mol^–1^ s^–1^). This indicates that electron transfer from quinuclidine predominates in the photoredox cycle.

To further understand what happens in the reaction mixture, TA and TRIR experiments were performed in the presence of all reaction components, including NiCl_2_-dtbbpy (Figure 3D). By comparing the spectra of the reaction mixture with those from the previous experiments, we observed that the reaction mixture has a similar profile to the TRIR spectra with quinuclidine, with the formation of the amine radical cation and the reduced [Ir{dF(CF_3_)ppy}_2_dtbbpy] which subsequently decays to reform the parent. These results indicate that quinuclidine is the major quencher of the photocatalytic cycle. Finally, 4-bromoacetophenone and 1-hexanol did not quench the excited state of [Ir{dF(CF_3_)ppy}_2_dtbbpy]PF_6_.

Following this initial mechanistic assessment, we performed batch reactions (Table 1) to develop reaction conditions that remain homogeneous throughout and suited to performing the process in flow. A selection of bases was screened, which were intended to be soluble in MeCN (the reaction solvent), unlike K_2_CO_3_ as previously reported.^12^ Therefore, we first screened conditions to identify where insolubilities were not present (Table S2). These batch reactions were necessary because, despite quinuclidine leading to efficient quenching of the ^3^MLCT excited state of [Ir{dF(CF_3_)ppy}_2_dtbbpy]PF_6_ when stoichiometric amounts were used, a precipitate was observed to form at the higher concentrations needed for preparative–scale reactions, rather than spectroscopic investigations. This precipitate, presumably a quinuclidine salt, could lead to blockages and reaction failures in a flow reactor. Since TA and TRIR highlighted the amine as the major quencher of the system, we tested different bases based on their quencher ability as well as their expected solubility, and that of their salts, in MeCN. Not surprisingly, amines known to quench excited state Ir complexes, such as DABCO (Table 1, entry 7), gave a comparable yield to previously reported quinuclidine (Table 1, entry 7),^12,25^ but precipitation still occurred during the reaction, probably due to the formation of insoluble salts. TEA and DIPEA were also investigated, but poor yields were observed (Table S2). The use of TMG as the base gave a comparable yield to quinuclidine but without any precipitation, and subsequent batch optimization using TMG was performed (Table 1). Importantly, it was also found that the reaction using TMG in batch was complete in 2 h rather than 24 h (Table 1, entry 2).

**Table 1:** Optimization of Reaction Conditions Using TMG as Base

entry	changed condition	yield^a^ (%)
1	1 h	40
**2**	**2 h**	**78**
3	5 h	76
4	none	61
5	no base	n.d^c^
6	K_2_CO_3_ instead TMG	n.d^c^
7	QN instead of TMG^d^	59
8	DABCO instead of TMG^d^	53
9	3.5 W 457 nm LED	41
10	2 h, [Ru(bpy)_3_](PF_6_)_2_	13
11	Ir(ppy)_3_	63
12	2 h, Ir(ppy)_3_	4
13	3 h–72 h, dark	1–2^b^
14	3 h–72 h, dark, 60 °C	n.d^c^
15	3 h–72 h, no Ir	1^b^
16	3 h–72 h, no Ni	2^b^
17	3 h–72 h, no Ni and Ir	0–2^b^
18	3 h–72 h, no Ir, dark, 60 °C	1–5^b^
19	3 h–72 h, no Ni, dark, 60 °C	1–5^b^
20	3 h–72 h, no Ni or Ir, dark, 60 °C	0–3^b^

a Isolated yield.

b GC-FID yields.

c No product detected.

d Reaction started as homogeneous, but precipitation occurs during reaction.

It was noted in the batch assessment that the temperature of the reaction mixture increased above ambient due to the heat generated by the light source used for irradiation. Although heating the reaction mixture in the absence of light led to negligible yields (Table 1, entries 14, 18-20), indicating that a purely thermal mechanism is not the primary route to the product, we sought to verify whether any thermal effect on the overall reaction was apparent by performing TA and TRIR experiments at both 20 and 60 °C.

**Figure 4:**
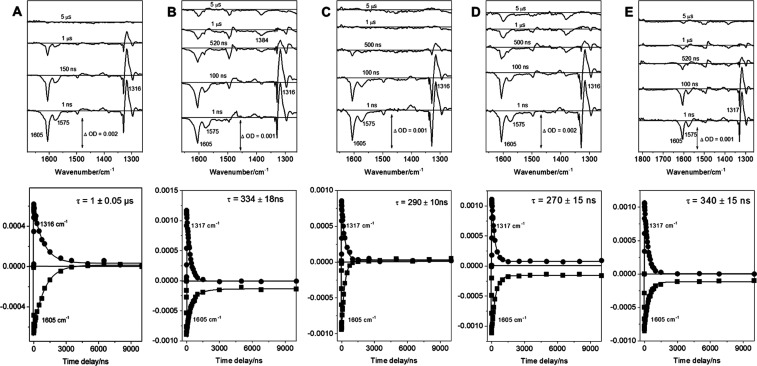
Selected ns-TRIR spectra and kinetics showing the ^3^MLCT triplet state of [Ir{dF(CF_3_)ppy}_2_dtbbpy]PF_6_ and its behavior in the presence of reaction components of the modified reaction conditions using TMG at 60 °C. Selected ns-TRIR spectra were obtained following 355 nm excitation of (A) 0.1 mM [Ir{dF(CF_3_)ppy}_2_dtbbpy]PF_6_ in CD_3_CN solution at 60 °C. (B) TRIR spectra in the presence of 10 mM TMG showing quenching of the ^3^MLCT triplet state of [Ir{dF(CF_3_)ppy}_2_dtbbpy]PF_6_. (C) TRIR spectra in the presence of 5 mM NiCl_2_-dtbbpy showing quenching of the ^3^MLCT triplet state of [Ir{dF(CF_3_)ppy}_2_dtbbpy]PF_6_ and no new detectable transient. (D) TRIR spectra in the presence of 10 mM TMG and 5 mM NiCl_2_-dtbbpy mixture. (E) TRIR spectra in the presence of 10 nM TMG, 5 mM NiCl_2_-dtbbpy, 10 mM 4-bromoacetophenone and 10 mM 1-hexanol mixture. It is possible to see a new transient around 1480 cm^–1^ in (B) attributed to reduced [Ir{dF(CF_3_)ppy}_2_dtbbpy]^.-^ species and its quenching in the presence of NiCl_2_-dtbbpy (D) and reaction components (E). See ESI for 20 °C spectra and respective TAs. Note that the TRIR spectra are offset, as in Figure 3.

The TA and TRIR were used to monitor the formation of the triplet state of [Ir{dF(CF_3_)ppy}_2_dtbbpy]PF_6_ photocatalyst at 20 or 60 °C. The TA and TRIR spectra at early times showed the features of the ^3^MLCT excited state of [Ir{dF(CF_3_)ppy}_2_dtbbpy]PF_6_, as described above and these results indicated that the temperature does not significantly affect the behavior of the ^3^MLCT excited state directly (Figure 4A). In the absence of other components, the TA band at 475 nm and TRIR signal at 1316 cm^–1^ decayed at the same rate (τ = 1.0 ± 0.1 μs) for both temperatures. However, the TA and TRIR spectra did suggest that an increase in temperature did have an effect on the dynamics of the ^3^MLCT excited state of [Ir{dF(CF_3_)ppy}_2_dtbbpy]PF_6_ and subsequent reactions when the Ir catalyst was irradiated in the presence of other reaction components.

In the presence of all components of the reaction mixture, the ^3^MLCT signal can be monitored using the TRIR band at 1316 cm^–1^, and the lifetime of the excited Ir photocatalyst decreased to 450 ± 10 ns at 20 °C and to 340 ± 15 ns at 60 °C (Figure 4E). These results indicate that the increased temperature probably resulted in more efficient quenching of the excited state of [Ir{dF(CF_3_)ppy}_2_dtbbpy]PF_6_. To further probe the temperature effects of the reaction, we investigated the influence of each component separately on the excited state dynamics of [Ir{dF(CF_3_)ppy}_2_dtbbpy]PF_6_ by TRIR.

TRIR experiments on [Ir{dF(CF_3_)ppy}_2_dtbbpy]PF_6_ in the presence of only NiCl_2_-dtbbpy (5 mM) showed faster decay of the ^3^MLCT excited state at similar rates at 20 and 60 °C (Figure 4C). A full bleach recovery is observed at both temperatures, again consistent with energy transfer with Ni reforming the ground state, and the TA experiments show the production of a new transient at 400 nm due to the excited state of NiCl_2_-dtbbpy at similar rates.

TRIR experiments on [Ir{dF(CF_3_)ppy}_2_dtbbpy]PF_6_ (1 mM) in the presence of 1,1,3,3-tetramethylguanidine (TMG) (10 mM) in place of quinuclidine as the reductive quencher showed faster decay of the ^3^MLCT excited state occurred at 500 (±20) ns at 20 °C and to 300 (±20) ns at 60 °C, indicating temperature dependence on this quenching process (Figure 4B). Similar to the experiments described above in the presence of quinuclidine, the TA spectra showed the formation of a new band at ca. 525 nm, which subsequently decayed at the same rate (τ = 2.5 ± 0.5 μs) as observed for the slower recovery of the parent bands in the TRIR experiments. We have analyzed the TRIR results further using global analysis, which is consistent with the assignments above.

### Development and Optimization of C–O Coupling in a Continuous Flow FEP Coil Reactor

Having developed a set of conditions suitable for flow processing, informed by TRIR spectroscopy and batch reaction screening, we began to investigate and optimize a flow process for the C–O coupling. Initially, a series of simple fluorinated ethylene propylene (FEP) coil flow reactors were constructed (see ESI for details).

Maintaining the conditions that we previously used in batch (0.2 M, 1 mol % Ir, 5 mol % Ni, 1.1 eq. TMG), we investigated different reaction space times (residence time in the irradiated volume of the reactor) to obtain a rapid view of the processing conditions (Table 2, entries 1-4). As expected, we observed that a decrease in the space time (faster flow rate) led to a decrease in yield. The yield was slightly higher on going from a space time of 5 min (Table 2, entry 3) to that at 10 min (Table 2, entry 4). However, a 5 min space-time gave a higher throughput and estimated productivity of 41 g day^–1^ compared to 23 g day^–1^ for the 10 min space-time. Additionally, control experiments without catalysts (Table 2, entry 5) resulted in no product detectable by GC analysis and no conversion of starting material. Similarly, only trace amounts of product were observed in the presence of a single catalyst, either Ir or Ni (Table 2, entries 6 and 7), indicating S_N_Ar or thermal Ni-catalyzed cross-coupling was not operating.

**Table 2:**
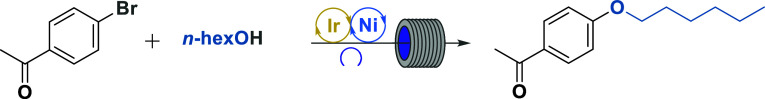
Optimization and Development of a Flow Process for the Metallaphotoredox C–O Coupling Reaction Using an FEP Coil Flow Reactor.^a^

entry	space time (min)	Ir load. (mol %)	Ni load.^b^ (mol %)	Conv (%)	yield^c^ (%)
1	1	1	5	39 ± 3	31 ± 1
2	2.5	1	5	71 ± 6	69 ± 3
3	5	1	5	90 ± 2	85 ± 4
4	10	1	5	>99	97 ± 3
5	2.5				n.d^d^
6	2.5	1			<1
7	2.5		5		<1
8	1	1	5	39 ± 3	31 ± 1
9	5	1	2.5	98 ± < 1	93 ± 4
10	5	1	1	59 ± 5	49 ± 8
11	5	1	0.5	27 ± 3	19 ± 1
**12**	**5**	**0.5**	**2.5**	**93 ± 1**	**90 ± 6**
**13**	**5**	**0.1**	**2.5**	**61 ± 8**	**59 ± 9**
14	5	0.1	1	30 ± 2	24 ± 2

a Reaction conditions: 4-bromoacetophenone 0.2 M in MeCN, 1.6 eq 1-hexanol, 1.1 eq. TMG, × mol % [Ir{dF(CF_3_)ppy}_2_dtbbpy]PF_6_, 410 nm LEDs, ∼ 60–70 °C (heat from LED without cooling), 3.8 mL FEP coil flow reactor. (See ESI for further details).

b The mol % loadings of NiCl_2_-glyme and dtbbpy ligand were kept equal.

c GC-FID yields (see ESI).

d No product detected.

We investigated whether efficient light penetration and improved heat/mass transfer in flow could lead to lower concentrations of metal-based catalyst needed, thereby improving the processing metrics, which become a much more important consideration on scale-up. First, we varied the Ni catalyst loading used in the reaction (Table 2, entries 8-10). In these experiments, it was observed that a loading of 2.5 mol % Ni catalyst (Table 2, entry 8) gave yields comparable to those obtained from previous experiments using twice the amount of Ni catalyst (Table 2, entry 2). This is consistent with previously reported observations^25^ that high Ni catalyst concentrations can lead to the formation of a mixed-valent Ni(I/III) dimeric complex that inhibits the reaction. A further decrease in the concentration of the Ni catalyst from 2.5 mol % to 1 or 0.5 mol % resulted in lower yields (Table 2, entry 10-11). Ultrafast spectroscopy experiments also highlighted a concentration dependence on the lifetime of the excited state of the photocatalyst. We have applied lower photocatalyst loadings, and it was observed that the Ir photocatalyst loading, like the Ni cocatalyst loading, could also be halved (Table 2, entry 12) without substantial change in yield while maintaining a short space time of 5 min. Notably, a 10-fold reduction of Ir photocatalyst to 0.1 mol % (Table 2, entry 13) still resulted in a moderate yield of 60%, possibly due to an improved light penetration and the decreased photocatalyst self-quenching. Finally, we also observed fair yields with a 10-fold reduction of the Ir catalyst and a 5-fold reduction in the Ni catalyst (Table 2, entry 14).

As previously discussed, our TRIR studies have demonstrated a reduced lifetime of the excited state of [Ir{dF(CF_3_)ppy}_2_dtbbpy]^+^ in the presence of TMG at elevated temperatures. Ni catalysts have been shown to facilitate the *O*-arylation of aliphatic alcohols under high temperatures and using strong bases and complex Ni catalysts.^13,49,50^ While photoredox catalysis facilitates the rate-limiting C–O bond-forming step by modulation of Ni oxidation state to allow for a favorable Ni(III)–Ni(I) reductive elimination,^12^ we hypothesized that a synergistic application of light and temperature could further accelerate this reaction based on our spectroscopic observations and in the same way that temperature is often a key component of conventional cross-coupling reactions. This would allow shorter space times and improve the overall throughput of the system.

**Figure 5:**
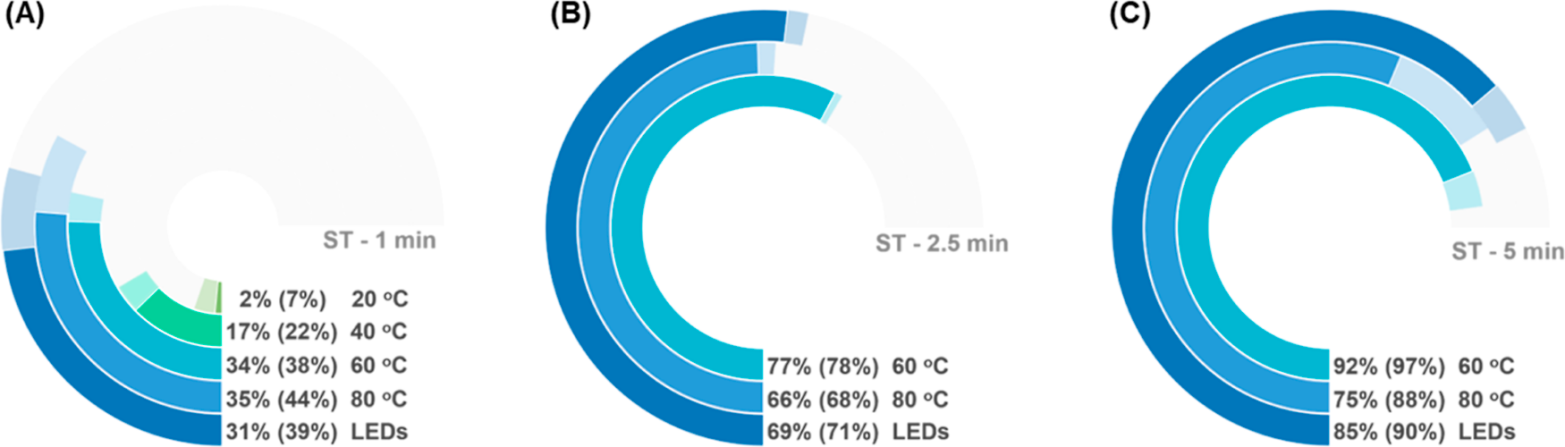
Schematic summary of the effects of temperature on the C–O coupling at (A) 1 min, (B) 2.5 min and, (C) 5 min space-time. Yields are shown as solid bars, with values displayed on the chart. Conversions are shown as lighter bars, with values displayed in brackets. LEDs = heat provided by the LED source without cooling on reaction, the average temperature measured was about 60–70 °C. Reaction conditions: 4-bromoacetophenone 0.2 M in MeCN, 1.6 eq 1-hexanol, 1.1 eq. TMG, 1 mol % [Ir{dF(CF_3_)ppy}_2_dtbbpy]PF_6_, 5 mol % NiCl_2_-glyme, 5 mol % dtbbpy, 410 nm LEDs, 3.8 mL FEP coil flow reactor.

We performed experiments in a coiled flow reactor across a range of temperatures from 20 to 80 °C. Our reactor (see ESI) consisted of a coil of FEP tubing wrapped around a jacketed borosilicate glass tube; the temperature of the tubing could be controlled by recirculating heating/cooling fluid through the jacket. Alternatively, the reaction could be heated by the excess heat from the LED lighting assembly inside the glass tube, which produced a temperature around 60 °C (termed below “LED heating”). The latter was investigated to verify if active cooling/heating of the reaction was detrimental to the reaction rate and if LEDs alone could be used to provide sufficient control over the reaction temperature. The latter was performed to understand how active cooling/heating of the reaction affects the reaction rate and if LEDs were providing not only photons but also increased heating to drive the reaction. As our starting point we chose conditions that gave less than 100% conversion (so-called “stressed conditions”, see Table 2, entry 1). The yields at 20 and 40 °C were notably lower than those observed at 60 and 80 °C (Figure 5a and Table S3) and with the LED heating. These findings suggest the involvement of a photothermal component, potentially in conjunction with acceleration of the Ni catalytic cycle.

Further investigation in the 60–80 °C range (Figure 5b–c) with extended residence times (slower flow rates) of 2.5 and 5 min revealed lower yields at 80 °C compared to 60 °C for both durations. The yields at 60 °C were comparable to those obtained without the application of a heating/cooling fluid, unsurprising as these were essentially at the same temperature as induced by the LEDs. Notably, the outlet stream from the 80 °C experiments contained a black precipitate, likely attributable to the aggregation and precipitation of Ni(0) as Ni black-a deactivation pathway previously identified in related dual photoredox/Ni-catalyzed C–N cross-coupling reactions.^51,52^ In the absence of light, no product was detected by GC, indicating the absence of a purely thermal mechanism (Table S3, entry 12).

We next established the dependence on reaction concentration, maintaining the conditions informed by the initial flow investigations working at 60 °C with reduced catalyst loadings. These results are summarized in Table 3.

**Table 3:**
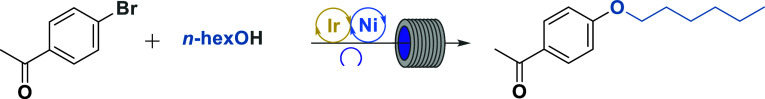
Concentration Optimization in Flow Using an FEP Coil Flow Reactor.^a^

entry	conc. (M)	space time (min)	conv. (%)	yield^b^ (%)
1	0.23	5	39 ± 1	32 ± 2
2	0.43	5	65 ± 1	63 ± 4
**3**	**0.47**	**10**	**97 ± 1**	**96 ± 1**
**4**	**0.85**	**10**	**85 ± 1**	**84 ± 2**
5	1.14	10	46 ± 3	33 ± 2
6^c^	0.22	5	97 ± 1	96 ± 1
**7**^c^	**0.85**	**5**	**92 ± 1**	**87 ± 2**
8^c^	0.85	10	>99	89 ± 3

a Reaction conditions: 4-bromoacetophenone **x**M in MeCN, 1.6 eq 1-hexanol, 1.1 eq. TMG, 0.1 mol % [Ir{dF(CF_3_)ppy}_2_dtbbpy]PF_6_, 1 mol % NiCl_2_-glyme, 1 mol % dtbbpy, 410 nm LEDs, ∼ 60–70 °C (heat from LED without cooling), 3.8 mL FEP flow reactor.

b NMR yields.

c 0.5 mol % [Ir{dF(CF_3_)ppy}_2_dtbbpy]PF_6_ and 2.5 mol % NiCl_2_-glyme and dtbbpy.

Employing reduced catalyst loadings of 0.1 mol % Ir and 1 mol % Ni loadings, we observed that increasing the concentration of 4-bromoacetophenone (from 0.2 to 0.43–0.85 M) led to an increase in yield/conversion (Table 3, entries 1-4). However, a significant decrease in yield and conversion occurs when the concentration is increased any further (Table 3, entry 5). Increasing catalyst loadings to 0.5 mol % Ir and 2.5 mol % Ni gave over 90% conversion and good yields at a concentration of 0.85 M in only 5 min space time (Table 3, entry 7). It was found that, for 0.85 M or higher concentrations, the outlet tubing had to be maintained above 40 °C to avoid precipitation of the TMG-salt formed in the reaction, which would otherwise lead to blockages in the tubing.

### Substrate Scope for Metallaphotoredox C–O Coupling in Continuous Flow

**Figure 6:**
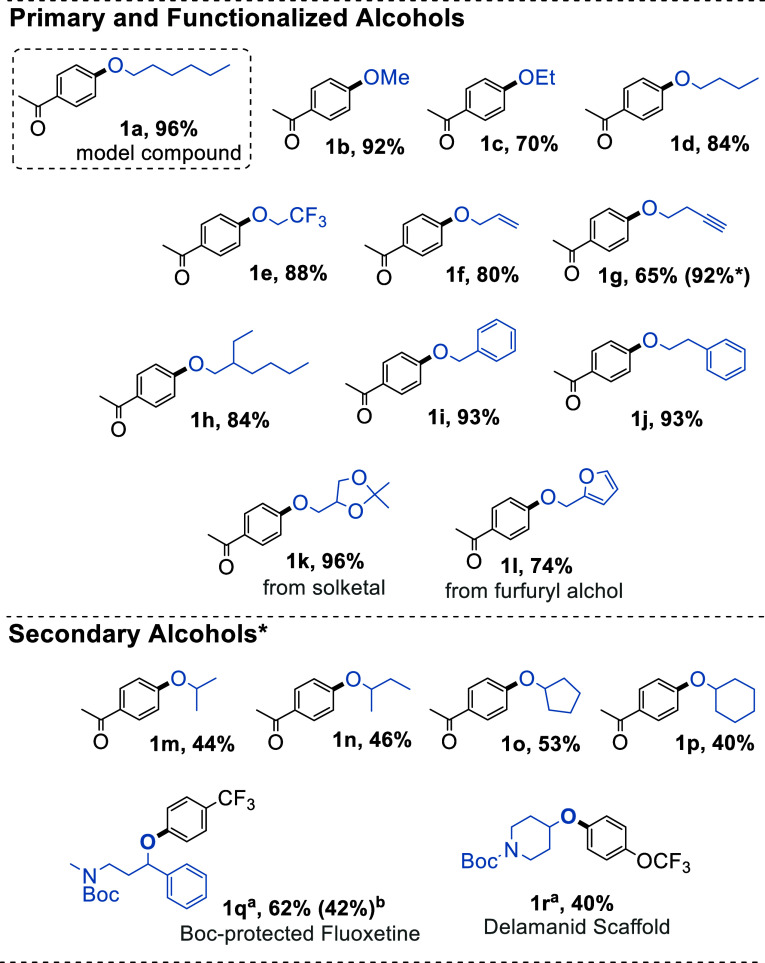
Reaction scope for the metallaphotoredox C–O coupling in flow. Reaction conditions: 1 eq. aryl halide, 1.6 eq. alcohol, 1.1 eq. base, 0.1 mol % [Ir{dF(CF_3_)ppy}_2_dtbbpy]PF_6_, 1 mol % NiCl_2_-glyme, 1 mol % dtbbpy, MeCN 0.47 M, 410 nm LEDs, ∼ 60–70 °C (LED temp.), 10 min space time, 3.8 mL FEP reactor. *20 min space-time for secondary alcohols. ^a^1 eq. of alcohol. ^b^10 min space time.

We have explored the scope of coupling partners used in the reaction to determine the applicability of this method for different substrates, including pharmacologically and industrially relevant scaffolds. Due to the high yields and low catalyst loadings employed, the study was carried out using the conditions described in entry 3 of Table 3. A range of primary alcohols was coupled in good to excellent yields (including alkyl, alkene, alkyne, aromatic, and heteroatom-containing compounds, Figure 6). Secondary alcohols could also be used, but they were lower yielding than primary alcohols and required at least doubled space time to achieve moderate yields. No conversion was observed for tertiary alcohols. Interestingly, etherification of biomass-based furfuryl alcohol was obtained with good yield (**1L**). Similarly, the protected glycerol derivative solketal was converted to the corresponding ether with excellent yield (**1k**). We also examined pharmaceutical-related molecules, demonstrating the synthesis of N-Boc fluoxetine (**1q**) with a moderate yield of 62% for the coupling step. Near quantitative yields were obtained for the protection and deprotection steps, leading to a 3-step synthesis for the antidepressant fluoxetine with an overall yield of 58% from commercially available building blocks. In addition, antituberculosis delamanid-related compound **1r** could be prepared with a 40% yield.

### Scale-Up of C–O Coupling in an FEP Coil Flow Reactor

We have investigated the scalability and determined the maximum process productivity on our FEP flow reactors. The reactions were performed in larger volume FEP coil reactors (see ESI), using the optimized conditions described in Table 3 (entries 4 and 7). Initially, we doubled the length of the FEP coil using two FEP coils (1/16″ OD, 1/32″ ID), giving a total irradiated volume of 7.6 mL. We also employed a dimensioning style approach, using a larger diameter (1/8″ OD, 1/16″ ID) FEP coil with an irradiated volume of 15.2 mL (see Table 4). Similar approaches have been previously used to scale up photochemical reactions to an industrial scale.^53^

**Table 4:**
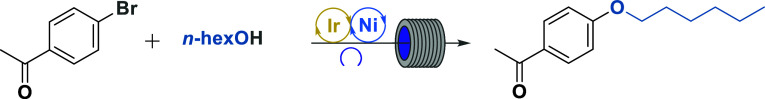
Scale-up of the Metallaphotoredox C–O Coupling Using a Numbering-up and Dimensioning Approach in FEP Coil Flow Reactors.^a^

entry	Ir load. (mol %)	Ni load. (mol %)	reactor vol. (mL)	space time (min)	conv. (%)	yield (%)	extrapolated productivity (g day^–1^)	STY (mol day–1 mL–1)
1	0.1	1.0	3.8	10	85 ± 1	84 ± 2	86	0.10
2	0.5	2.5	3.8	5	92 ± 1	87 ± 2	174	0.21
3	0.1	1.0	7.6	10	99 ± 1	89 ± 3	180	0.11
4	0.5	2.5	7.6	5	71 ± 1	70 ± 1	290	0.17
5	0.1	1.0	15.2	10	77 ± 3	63 ± 2	255	0.08
**6**	**0.5**	**2.5**	**15.2**	**10**	**90±1**	**83±3**	**340**	**0.10**
7^b^	0.1	1.0	15.2	10	92 ± 2	85 ± 4	190	0.06

a Reaction conditions: 1 eq. aryl halide 0.85 M, 1.6 eq. alcohol, 1.1 eq. TMG, MeCN. 60 °C applied using a heating jacket.

b 0.47 M instead of 0.85 M.

Increasing the length of the 1/16″ OD FEP coils resulted in good yields while still employing lowered catalyst loadings (Table 4, entries 3 and 4). Not surprisingly, the projected productivity obtained in this approach was around double that of the smaller-scale reactor, reaching an estimated value of ca. 300 g day^–1^ vs 175 g day^–1^ for the original, smaller FEP reactor (3.8 mL). Increasing the diameter of the tubing (Table 4, entries 5-7) also resulted in higher projected productivities, up to an estimated value of ca. 340 g day^–1^ (Table 4, entry 6). However, for similar conditions a lower yield was observed in the larger bore tubing reactor compared to the smaller bore reactors (Table 4, entries 3 and 5). Lowering the starting material concentration resulted in good conversion and yields at both Ir/Ni loadings employed (Table 4, entry 7).

### Scale-Up of C–O Coupling in Photochemical Taylor Vortex Flow Reactors (PhotoVortex)

**Figure 7:**
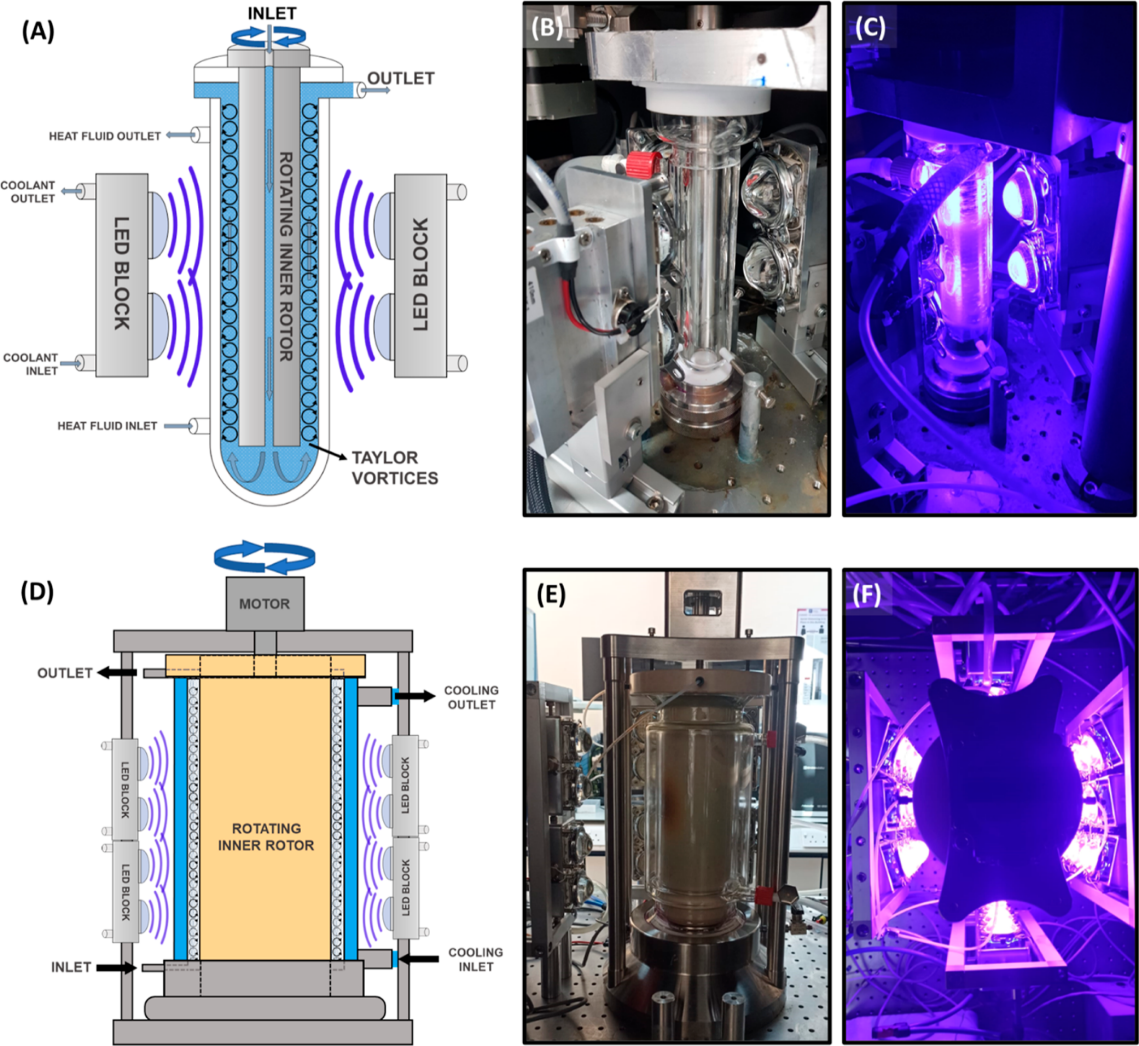
Schematic views (vertical cross-section, not to scale) of (A) the PhotoVortex and (D) the scaled-up PhotoVortex (B) Photograph of the PhotoVortex showing the jacket, rotor and the modified 410 nm high-powered LEDs (600W, 3 × 200W blocks) with LEDs turned off and (C) LEDs turned on. (E) Photograph of the large PhotoVortex showing the jacket, rotor and half of the modified 410 nm high-powered LEDs (3 kW, 15 × 200W blocks). (F) Photograph of the large PhotoVortex taken from directly above when LEDs are turned on to illustrate the configuration of the LEDs around the reactor.

In order to scale-up photochemical reactions further, we have investigated the use of our scalable continuous Photochemical Taylor Vortex Flow Reactor (PhotoVortex).^34,35^ This design consists of an outer glass jacket with a central stainless steel or polymer rotor, creating a narrow annular path for the reaction solution to flow through the gap between the rotor and the glass jacket. Rapid rotation (high RPM) of the central rotor has been demonstrated to generate Taylor vortices in the solution flowing through the narrow channel in the reactor, resulting in highly efficient mixing between, for example, gas and liquid phases in photooxidation reactions.^32^ The increased mass transfer due to the vortices could benefit other photochemical systems. The vortices create fast mixing, which ensures a fast renewal of fresh material close to the irradiated surface so that all of the molecules in the solution are exposed to light. Initially, we probed the use of PhotoVortex reactors for metallaphotoredox C–O coupling using our original design,^35^ where the reactor was equipped with white light LEDs (i.e., broadband visible light). However, there was poor overlap between the photocatalyst absorption bands and the emission profile of the white light LEDs. As photochemical reactions are strongly dependent on the wavelength and intensity of the light source,^39,54^ we modified our original design using three high-intensity blue-violet LED blocks (3 × 200W 410 nm) and we were able to obtain high productivities in the PhotoVortex (Figure 7A–C).

**Figure 8:**
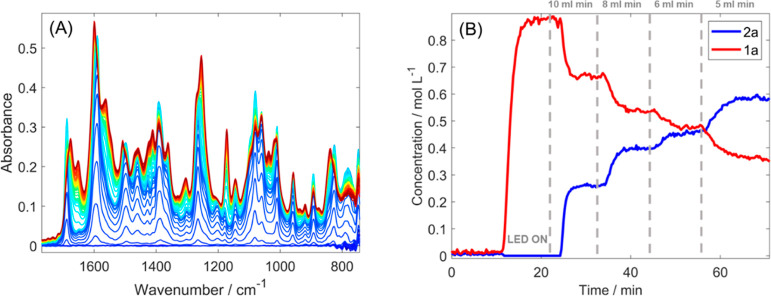
(A) FTIR spectra of the solution flowing out of the reactor and (B) MCR concentrations of reaction components obtained predicted from the online FTIR spectra.

Process analytical technologies (PAT) are key for the online monitoring of parameters that affect the efficiency of processes. Therefore, we utilized inline FTIR (Figure 8) to observe the reactor output and determine the response to the changing parameters. The spectra obtained were processed via multivariate curve resolution (MCR) to obtain pure component spectra and their respective concentration profiles. The results allowed us not only to monitor changes due to reactant consumption and product formation but also to obtain the concentrations of both starting materials and product 1a (Figure 8) with good agreement with offline NMR and GC-FID analysis. Therefore, we used inline FTIR to access the process optimization on the Vortex Reactor.

The reactions were initially performed using the same conditions as optimized for the 7.6 mL FEP coil reactor in Table 4 (entry 3), as the irradiated volume of the PhotoVortex was very similar (8 mL irradiated volume). However, using the PhotoVortex we were able to reduce the required space time to get high conversion and yield significantly from 10 min for the FEP to only 2 min for the PhotoVortex (Table 5, entry 2). Notably, we achieved a projected productivity of 870 g day^–1^ under these conditions. This corresponds to a space-time yield of ∼0.5 mol day^–1^ mL^–1^ compared to a maximum of ∼0.2 mol day^–1^ mL^–1^ in the FEP tubular reactor. Further optimization was performed varying space time (flow rate), the rotation speed of the PhotoVortex inner rotor, and catalyst loadings. Reducing the space time from 2 to 1.6 min (Table 5, entry 3) led to a small decrease in conversion and yield. However, the projected productivity increased to > 1 kg day^–1^ (STY = 0.57 mol day^–1^ mL^–1^). This represents more than 500-fold increase in processing productivity compared to the initial disclosure of the reaction (batch 1 mmol in 6 mL for 24 h).^12^ Changing the rotation speed from 2000 to 3000 rpm did not change conversion or yield significantly (Table 5, entry 7), but a further increase to 4000 rpm or a decrease to 1250 rpm resulted in decreased yields (Table 5, entry 6 and 8) consistent with the lower rotation speed leading to less efficient mixing and the higher rotation resulting in a turbulent flow.

**Table 5:**
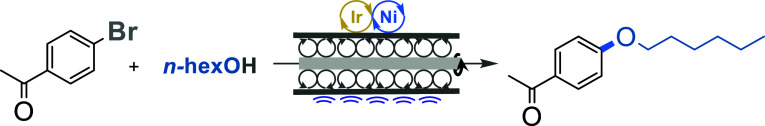
Metallaphotoredox C–O Coupling in the PhotoVortex.^a^

entry	conc. (M)	space time (min)	conv. (%)	yield (%)	extrapolated productivity (g day^–1^)	STY (mol day–1 mL–1)
small PhotoVortex						
1	0.81	2.7^f^	93 ± 4	80 ± 3	610	0.35
**2**	**0.82**	**2.0**	**90±2**	**85±1**	**870**	**0.49**
**3**	**0.82**	**1.6**	**84±2**	**78±2**	**1000**	**0.57**
4	0.81	1.3	70 ± 3	56 ± 3	860	0.49
5	0.85	0.8	47 ± 3	34 ± 5	920	0.53
6^b^	0.82	1.6	82 ± 2	71 ± 3	930	0.53
7^c^	0.82	1.6	80 ± 1	77 ± 1	1000	0.57
8^d^	0.82	1.6	78 ± 2	62 ± 3	810	0.46
9^e^	0.81	8.0^f^	58 ± 4	54 ± 3	140	0.08
10^e^	0.81	4.0	67 ± 1	62 ± 1	310	0.18
11^e^	0.81	2.7	63 ± 1	58 ± 1	450	0.26
large PhotoVortex						
12	0.85	3.0	59	47 ± 2	7830	0.19
**13**	**0.85**	**4.0**	**94**	**90±2**	**11,100**	**0.27**
14	0.85	6.0	95	94 ± 3	7800	0.19
15^e^	0.85	4.0	52	43 ± 1	5400	0.13
**16**^e^	**0.85**	**6.0**	**97**	**95±1**	**7900**	**0.19**
17^e^	0.85	8.0	95	93 ± 3	5742	0.14

a Reaction conditions: 1 eq. aryl halide, 1.6 eq. alcohol, 1.1 eq. TMG, 0.5 mol % [Ir{dF(CF_3_)ppy}_2_dtbbpy]PF_6_, 2.5 mol % NiCl_2_-glyme, 2.5 mol % dtbbpy, MeCN, PhotoVortex, 2000 rpm, 65 °C.

b 1250 rpm.

c 3000 rpm.

d 4000 rpm.

e 0.1 mol %Ir and 1 mol %. STY = productivity/reactor irradiated volume (8 mL for Small PhotoVortex or 185 mL for Large PhotoVortex).

f Black fouling observed.

We also investigated the use of lower catalyst loadings (0.1 mol % Ir and 1 mol % Ni) and moderate to good yields were obtained, with productivity up to 450 g day^–1^. Thus, a 5-fold decrease in catalyst loading only resulted in halving the process productivity. Attempts to further increase the yield by increasing the space time were unsuccessful due to the formation of a black precipitate at lower flow rates (Table 5, entries 1 and 9). This was attributed to the formation of Ni black, as commented above.

The advantages of continuous flow photochemistry for further scale-up are exemplified in various examples.^39,55,56^ Nevertheless, the transfer of these reactions from a laboratory scale to production scale (>1 kg/day) is still challenging.^57^ We recently described a scaled-up Photochemical Taylor Vortex Flow Reactor, namely a large PhotoVortex (185 mL irradiated volume, 280 mL total volume). Using similar conditions to those in the smaller PhotoVortex, we were able to achieve an increase in projected productivity up to around 11 kg per day (Table 5, entry 13), equivalent to ca. 4 tonnes per year. This represents a 13× increase in productivity while maintaining the yield at 90% (Table 5, entries 3 and 13). The large reactor uses a 2 mm path length, double that of the smaller PhotoVortex (1 mm). We were able to achieve these high yields without any noticeable fouling of the glass body of the reactor. Next, we investigated lowering the catalyst loading to 0.1 mol % (Table 5, entries 15-17). By only increasing the space-time in the reactor to 6 min, we obtained a 95% yield (Table 5, entry 16), achieving projected productivity of ca. 8 kg per day with a 5× lower concentration of catalyst. We believe that these results demonstrate the robustness of the PhotoVortex reactor for scaling up photoredox catalysis to the production scale. Typically, a larger reactor was only run for a total of 5 h at a time, so all our productivities are extrapolated to 24 h. In addition, it was found that for the PhotoVortex reactors, the outlet tubing needed to be externally heated using a heating cell to maintain the temperature above 40 °C and prevent precipitation of the TMG-salt formed during the reaction, ensuring the smooth performance of the reactor. Only when the outlet was not heated, the TMG-salt precipitated, leading to blockages.

Despite metallaphotoredox catalysis presenting an efficient approach for bond construction and having demonstrated an approach to scale-up, the use of precious metal Ir photocatalysts requires consideration of sustainability and metal recovery. Therefore, we also explored a laboratory strategy for recovering the Ir photocatalyst used in this C–O coupling reaction in the small Photovotex. We were able to recover 86 (±5)% of the Ir photocatalyst simply by extending the column chromatographic purification using more polar eluents (see experimental section for details). The recovered catalyst shows retention of structure and activity over 5 different runs on the small PhotoVortex reactor (see ESI). Alternative methods are under consideration, which involve using heterogenized metal complexes^58−60^ or membrane separation technology.^61^ We have demonstrated high productivity metallaphotoredox reactions (>10 kg day^–1^) in a very small footprint PhotoVortex reactor, and coupling this with the possibility of employing catalyst recovery offers an opportunity for further process development in photoredox catalysis.

## Conclusion

We have developed a protocol for scalable continuous flow C–O coupling for the synthesis of alkyl-aryl ethers synthesis using dual metallaphotoredox catalysis. We have investigated the dual Ir/Ni metallaphotoredox C–O coupling reaction using TRIR spectroscopy, which supporting the accepted mechanism with the amine acting as a reductant of the excited state ^3^MLCT excited state of [Ir{dF(CF_3_)ppy}_2_dtbbpy]PF_6_. We also observed detectable amounts of an Ir(II) species that activate the Ni(II) cocatalyst to a Ni(I) species via reduction by the photogenerated Ir(II). Spectroscopic measurements suggested that the nature of the base, temperature, and catalyst concentration are the main mechanistic factors for process optimization. Notably, the reaction outcome was considerably impacted by temperature, supporting that photothermal mechanisms are involved in agreement with spectroscopic observations. An initial investigation of this transformation was carried out using a simple to construct FEP tubular flow photoreactor and excellent yields were observed. The versatility of our protocol has been demonstrated with 18 different examples, including the valorization of biomass (furfuryl alcohol and solketal) and the preparation of pharmaceuticals/pharmaceutical derivatives, including WHO essential medicine fluoxetine. The process was demonstrated on a projected 340 g day^–1^ scale simply by scaling-up the flow reactor. Finally, our PhotoVortex reactors with high-intensity 405 nm LED light sources were shown to provide the productivities required for small scale manufacture, achieving >1 kg day^–1^ projected productivities (STY = 0.57 mol day^–1^ mL^–1^) with the small PhotoVortex (ca. 8 mL) while the larger PhotoVortex (ca. 185 mL) gave production scale projected productivities up to 11 kg day^–1^ (STY = 0.19 mol day^–1^ mL^–1^). We believe that these results are highly promising for the scale-up of dual metallaphotoredox catalysis and present considerable opportunities for synthesizing pharmacologically and industrially relevant compounds.

## Safety Note

These experiments involve the use of high intensity light sources (410 nm). It is the responsibility of each researcher to take appropriate precautions depending on the apparatus used to repeat this work. In particular, LEDs should be housed in a suitable light-tight enclosure and operators should wear appropriate eye protection and PPE to avoid skin exposure. Particularly, 410 nm LEDs can cause eye damage and protection should always be worn. All experiments were performed in well-ventilated fume cupboards.

## Supporting Information Available

description of the reactors, ultrafast spectroscopic data, structural assignments, and NMR spectra for compounds (PDF)
