# Supplementary material for: Scale-Up of Continuous Metallaphotoredox Catalyzed C–O Coupling to a 10 kg-Scale Using Small Footprint Photochemical Taylor Vortex Flow Reactors
Source: Org Process Res Dev. 2024 Dec 4;29(1):34–47. doi: 10.1021/acs.oprd.4c00262 (PMC11744928; doi:10.1021/acs.oprd.4c00262)
Supplement: Supplementary file 1 — op4c00262_si_001.pdf [file op4c00262_si_001.pdf]

Electronic Supporting Information

Scale-up of Continuous Metallaphotoredox Catalyzed C-O Coupling to a 10 kg-Scale Using Small Footprint Photochemical Taylor Vortex Flow Reactors

Rodolfo I. Teixeira,[§] Toby H. Waldron Clarke,[§] Ashley Love, Xue-Zhong Sun, Surajit Kayal, and Michael W. George*

School of Chemistry, The University of Nottingham, University Park, Nottingham, NG7 2RD, UK.

Keywords: Scale-up, Flow Photochemistry, Taylor Vortex Reactor, Photocatalysis, MetallaPhotoredox, Ultrafast Spectroscopy.
*corresponding author: michael.george@nottingham.ac.uk
§These authors contributed equally to this work

Experimental

General

Reagents, solvents and gases were purchased from commercial suppliers and used without further purification, unless otherwise described. Photocatalysts were supplied by Johnson Matthey as part of their PGM catalysts. Stainless steel tubing, fittings and connections were purchased from Swagelok. FEP tubing was purchased from Cole-Parmer, and plastic fittings/connections were purchased from commercial sources (Gilson, Omni-Fit). IR spectra were recorded using a Thermo Nicolet 6700 FT-IR instrument.

Automatic column chromatography was performed using a Teledyne ISCO CombiFlash Rf + system, using UV detection. GC analyses were carried out using a Shimadzu GC-2014 system equipped with a 30 m length, 0.25 mm diameter Supelco Equity-1701 column (0.25 µm particles, fused silica with polycyanopropylphenyl/polydimethylsiloxane bonded phase – intermediate polarity. A temperature gradient from 50 °C to 280 °C, over 15 min, then holding at 280 °C for a further 5 min. Flame ionisation detection was used, with a temperature of 300 °C, 4 µL injection volume of sample (50.0 split ratio) and He as the carrier gas (column flow rate of 1.36 mL min^{-1}).

Proton nuclear magnetic resonance (^{1}H NMR) spectra and proton-decoupled carbon nuclear magnetic resonance (^{13}C NMR) spectra were recorded at 25 °C (unless stated otherwise) using AV400 (400 MHz) and AV(III)400hd (400 MHz) spectrometers. Chemical shifts for proton are reported in parts per million downfield from tetramethylsilane and are referenced to residual protium in the NMR solvent according to values reported in the literature. Chemical shifts for carbon are reported in parts per million downfield from tetramethylsilane and are referenced to the carbon resonances of the solvent. Data are represented as follows: chemical shift, integration, multiplicity (s = singlet, d = doublet, t = triplet, q = quartet, dd = doublet doublet, ddd = doublet doublet doublet, dt = doublet triplet, tdt = triplet doublet triplet, m = multiplet), coupling constants (J) is in Hertz (Hz). NMR spectra were processed with MestReNova Software (v 14.0.0-23239).

Yields and conversions were determined in triplicate by ^{1}H NMR against trimetroxybenzene as an external standard and samples were taken as the reactor was at steady state (3 reactor

volumes). Samples of 1-2 mL were taken from the reactor when running in its steady state, and aliquots of 100uL were concentrated using a nitrogen flow. The external standard was added from a stock solution of trimethoxybenzene in MeCN-d_3 (typically ~5 mg ml^{-1}).

De-ionised water was used as the fluid in all recirculating chillers, in order to ensure that no contaminants were present that could interfere with the absorption of light.

Tubular reactor Design and Operation

The continuous flow reactors used in this work were built as part of the project and were based upon a Fluorinated Ethylene Propylene (FEP) coil continuous flow reactor. The scheme of the build reactor is show in the Figure below.

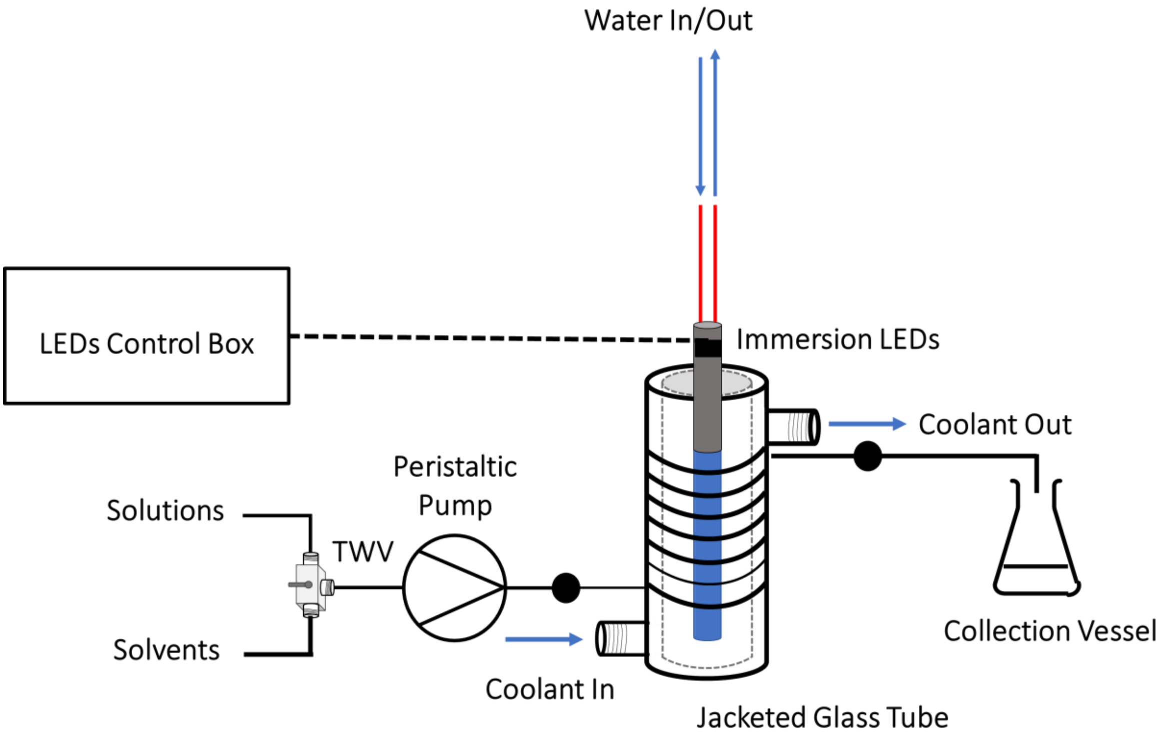

Figure S1. Diagram of the photochemical FEP flow reactor built. Solid black line (-) denotes FEP tubing, dashed black line (---) denotes electrical wiring, TWV = three-way valve.

Two lengths of 1/32” ID FEP tubing were pierced through two respective Suba-seals. To the opposite end of the Suba-seal “stopper side” was connected two Omni-Fit plastic connectors, held in place with a plastic ferrule. Each of these connectors was used to join the tubing lengths to an Omni-Fit three-way valve (TWV). This was intended to allow for attachment of two round bottomed flasks (to the Suba-seals) with the ability to close the three way-valve to the third outlet, allowing for each flask to be handled without exposure to the surrounding

atmosphere. The third outlet of the three-way valve could then be connected to peristaltic pump-head PTFE tubing (Masterflex, 2 mm ID, 4 mm OD, 38 cm length), using an Omni-Fit plastic connector. This tubing was used with a Masterflex L/S 77390-00 (Cole-Parmer) peristaltic pump and the tubing was inserted into the pump-head of the pump. To the outlet end of the peristaltic pump tubing was another Omni-Fit plastic connector. This connected the pump-head tubing to the irradiated reactor coil which was either a length of 1/8", 1/16" or 1/32" FEP tubing (details below). This tubing was coiled around a jacketed Pyrex tube (described below) and at the other end, was joined (again using Omni-Fit plastic connectors and appropriate seals/ferrules) to a short (*ca*. 30 cm) length of 1/32" ID FEP tubing, forming the reactor outlet. The jacketed tube was made in-house by the glassblowing workshop at the University of Nottingham. The jacket was designed to appropriately house the Lightsabre LED light source in the center and to allow for the tubing to be coiled around the outside, with a inner jacket of the tube was connected to a recirculating chiller/heater in between, The inner jacket was connected to a VWR-1162A recirculating chiller with the pump speed set to high and a thermocouple inserted into the cooling/heating bath to ensure the temperature was at the desired value. The coolant fluid was always 50/50 H_2O:ethylene glycol, or H_2O, maintaining transparency to visible light. The figure S2 show the image of the reactor in operation.

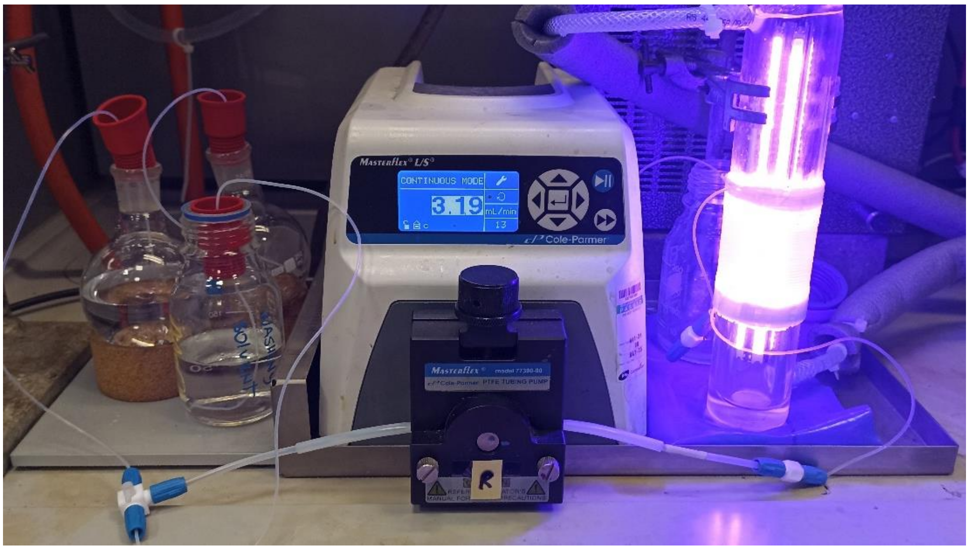

Figure S2. Photograph of the photochemical FEP flow reactor built in operation.

It was used three different volume reactors and the specifications of each piece of tubing used in this work as a reactor coil are given below.

Table S1. dimensions and specifications of tubing used as a reactor coil in this work containing the required information to calculate an internal volume of the reactor coil tubing.

Tubing	ID / “ (mm)	OD / “ (mm)	Wall Thickness / “ (mm)	Length / cm	Vol. Per cm / mL	Total Vol / mL
1/32 “	1/32 (0.79)	1/16 (1.59)	1/64 (0.40)	762	0.005	3.8
1/32 “*	As above			1524	0.005	7.6
1/16 “	1/16 (1.59)	1/8 (3.2)	1/32 (0.79)	762	0.020	15.2

**Tubing 4 consisted of 2 identical lengths of Tubing 3 joined together with an Omni-Fit connector and each coiled around the jacketed tube in the reactor system.*

The Immersion LED source involves 6 blue LED strips, mounted on a water-cooled hollow metal tube, with attachments for connecting water cooling and an electrical connection to the in-house built control box/power source. The LED emission maximum wavelength was measured experimentally to be 410 nm over the range of power (current) outputs available using the in-house built control box. The in-house built control box operates using a driver supplied by Mean Well, operating within 48 V and 3.2 A, giving a maximum power output of 153.6 W. For all the experiments performed, the value was set to the maximum value of 500 mA, and the power output was assumed to be slightly less than 140 W. The Immersion LED source, control box and emission spectrum (obtained previously) are shown below.

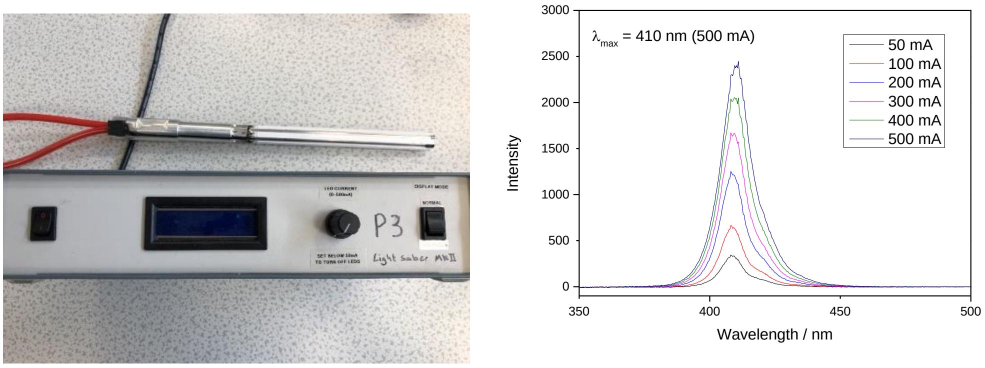

Figure S3. Immersion LED source used in the continuous flow reactor in this work and control unit (left) and output emission spectrum.

General Vortex Reactor Design and Operation

The reactor was used as described in Org. Process Res. Dev. 2017, 21, 1042 with modification on the light source. Three custom water-cooled blocks of 410nm LEDs were used. Each block contains 2 LED chips of 100 W each. The blocks were cooled in series by a single recirculating chiller (Julabo FL601).

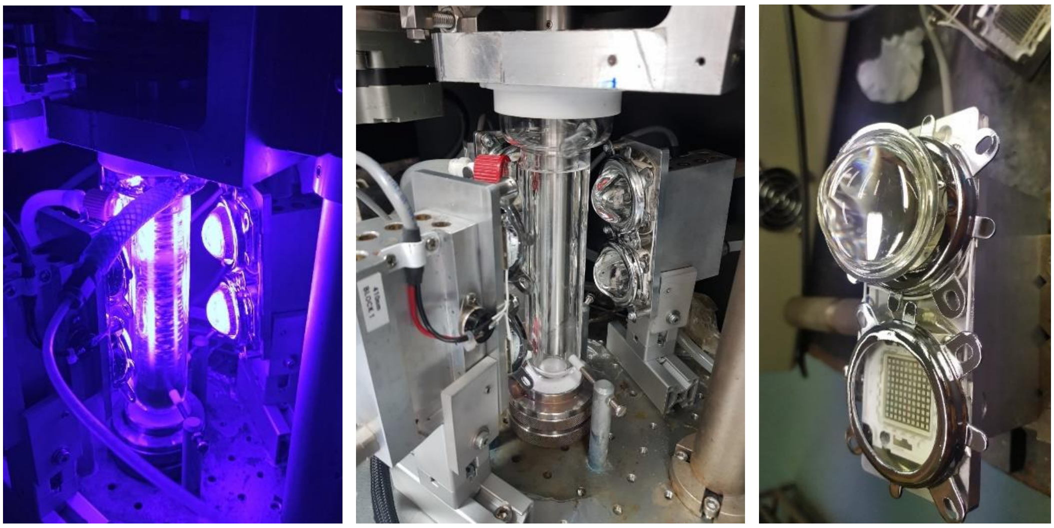

Figure S4. Photographs of the modified Vortex Reactor and LEDs.

The LED recirculating chiller was turned on and set to 10 °C whilst the reactor chiller was set to the appropriate temperature for the reaction (usually 60 °C). The chillers were left to equilibrate for ca. 30 min. The reactor was then flushed with N_2 at flushed at 10 mL min^{-1} for 10min to avoid oxygen. The reactor was then. The reaction mixture prepared as described below and on of the inlet pipes for the peristaltic pump was fed into the solution and the other inlet into degassed solvent (same as reaction). The desired flow rate was set on the peristaltic pump and the degassed solvent was pumped. After the reactor was filled with the degassed solvent, the gas flow was turned off. The peristaltic pump to remove the solutions from the reactor was set to double the inlet flow rate. The rotation motor was turned on and slowly increased until the desired rotation speed was achieved (typically 2000 rpm). The degassed solvent was flushed for 3 systems volumes (including tubbing, *c.a.* ~ 60 mL). The inlet Omni-Fit three-way valve was then switched to the reaction mixture. The LEDs were turned on at full brightness. Two full reactor volumes were allowed to pass before taking a sample for analysis, this ensured that the reactor had reached a steady state. Once the operation was complete, the LEDs turned off, and the rotation motor was slowly decreased to 0 rpm. The

recirculating chillers were also turned off, providing the LED blocks were not excessively hot. The inlet Omni-Fit three-way valve was then switched back to the container with solvent (usually the reaction solvent) and the reactor was flushed at 5 mL min^{-1}. The rotation speed was kept at 2000 rpm to ensure that all material was removed from the reactor. Once the reactor was clean, the motor and pumps were turned off.

Time-resolved Infrared Experiments

All spectroscopy samples were prepared in a flow system, where the material could be constantly pumped through the CaF2 windows of the Harrick cell, allowing for avoiding degradation of the sample. 390 μm PTFE spacers between the windows were used in all cases here (determining the path length). All solutions were made up CD_3CN solvent following here freeze-pump-thaw cycles and purging with Ar.

General Experimental Procedure for Batch Reactions

To solution of 4-bromoacetophenone (1mmol), $[Ir\{dF(CF_3)ppy\}_2(dtbbpy)]PF_6$ (1 mol%) in MeCN (2mL) was added the base (1.1 eq., 1.1 mmol, see tables bellow), and a pre-mixed solution of $NiCl_2$.glyme (5mol%) and dtbbpy (5mol%) in MeCN (2mL). The reaction solution was subjected to one freeze-pump-thaw cycle. 1-hexanol (1.6 mmol) was added the solution and then two further freeze-pump-thaw cycles were applied. The solution was then clamped ca. 1 cm from a white LED block and stirred overnight. Crude reaction mixture was diluted with EtOAc (10 mL) and washed with H_2O (10mL). The aqueous layer was further extract with EtOAc (2x10mL). The organic layers were combined and washed with brine (2 x 10 mL), dried over MgSO4 and combined, rotary evaporated give dry crude product and purified by using automatic column chromatography (95:5 cyclohexane/EtOAc, 4 g or 12 g Redisep Rf Gold column, 15 mLmin^{-1}, 10 min) to the desired product, 4-hexyloxy acetophenone, as a yellow oil (see yields in the tables bellow). ^{1}H NMR (400 MHz, Chloroform-d) δ 7.92 (d, J = 8.9 Hz, 2H), 6.92 (d, J = 8.9 Hz, 2H), 4.02 (t, J = 6.6 Hz, 2H), 2.55 (s, 3H), 1.89 – 1.72 (m, 2H), 1.52 – 1.40 (m, 2H), 1.34 (q, J = 3.6 Hz, 4H), 0.99 – 0.84 (m, 3H). ^{13}C NMR (101 MHz, $CDCl_3$) δ 196.95, 163.28, 130.73, 130.25, 114.28, 68.41, 31.68, 29.21, 26.48, 25.80, 22.73, 14.16.

Table S2. Base system optimization

Base System	Homogeneous System	Yield[a] (%)	pKa*
1 eq. K_2CO_3 + 0.1 eq. QN	N	69 ± 6[*b*]	
1.1 eq. QN	N[d]	59	11.0
1.1 eq. DABCO	N[d]	53	8.82
1.1 eq. TEA	Y	9[c]	10.75
1.1 eq. DIPEA	Y	24	10.75
**1.1 eq. TMG**	**Y**	**61**	**13.6**
1.1 eq. DBU	Y	7[e]	12.0
1.1 eq. TMAOH	N	--	Not found
1.1 eq. TBAOH	N	--	Not found
1.1 eq. Pyridine	Y	7	5.2
1.1 eq. DMAP	N	2	9.2
1.1 eq. imidazole	N	--	6.95

[a]Isolated yield. [b]Average of 4 isolated yields. [c]Estimated by ^{1}H NMR by product and SM co-eluted in chromatograph. [d]Started as homogeneous, but precipitation occurs during reaction. *pKa of conjugate acid, taken directly from the Ripin and Evans pKa table, unless noted.

General Experimental Procedure for FEP flow Reactions

To solution of 4-bromoacetophenone (1 eq.), [Ir{dF(CF_3)ppy}$_2$(dtbbpy)]PF_6 (0.1, 0.5 or 1 mol%), tetramethylguanidine (1.1 eq,) in MeCN, was added a pre-mixed solution of $NiCl_2$.glyme (1, 2.5 or 5mol%) and dtbbpy (1, 2.5 or 5mol%) in MeCN. The reaction solution was subjected to one freeze-pump-thaw cycle. The alcohol (1.6 mmol) was added to the solution and then two further freeze-pump-thaw cycles were applied. The mixtures were transferred and pumped through a flow reactor fit with a blue LED light source at a controlled temperature. Samples of product were collected and analysed using gas chromatograph and/or NMR.

General Experimental Procedure for Vortex flow Reactions

To solution of 4-bromoacetophenone (1 eq.), [Ir{dF(CF_3)ppy}$_2$(dtbbpy)]PF_6 (0.1 or 0.5mol%), was added a pre-mixed solution of $NiCl_2$.glyme (1, 2.5 or 5mol%) and dtbbpy (1 or 2.5 mol%) in MeCN (typically ~500mL, concentration ~0.85M). The reaction mixture was deaerated by bubbling with argon for about 60 min. tetramethylguanidine (1.1 eq.) and 1-hexanol (1.6 mmol) was added after 20 min and 40min under argon bubbling, respectively. The mixtures were transferred and pumped through the flow reactor as described above. Samples of product were collected after stationary state was achieve (~3 reactor volumes) and analysed using NMR.

Temperature Effect

Table S3. Temperature assessment in Flow

Entry	Temp. (°C)	Space Time (min)	Conv. (%)	Yield (%)
1	LED[a]	1	39 ± 3	31 ± 1
2	20	1	7 ± 3	2 ± <1
3	40	1	22 ± 4	17 ± 1
4	60	1	38 ± 4	34 ± 2
5	80	1	44 ± 3	35 ± 3
6	LED[a]	2.5	71 ± 6	69 ± 3
7	60	2.5	78 ± 4	77 ± 6
8	80	2.5	68 ± 2	66 ± 6
**9**	**LED[a]**	**5**	**90 ± 2**	**85 ± 4**
**10**	**60**	**5**	**97 ± 1**	**92 ± 4**
11	80	5	88 ± 2	75 ± 6
12	60[b]	5	2 ± 1	n.d.[c]

Reaction conditions: MeCN, 1.6 eq. 1-hexanol, 1.1 eq TMG, 1 mol% [Ir(dF(CF_3)$_2$dtbbpy)]PF_6, 5 mol% $NiCl_2$-glyme, 5 mol% dtbbpy , 410 nm Lightsaber LEDs, 3.8 mL FEP reactor. [a]Heat provided by the LED source without cooling on reaction, the average temperature measured was about 60-70ºC. [b]No light. [c]no product detected.

Substrate Scope

To solution of arylbromide (1 eq.), [Ir{dF(CF_3)ppy}$_2$(dtbbpy)]PF_6 (0.1mol%) and tetramethylguanidine (1.1 eq,) was added a pre-mixed solution of $NiCl_2$.glyme (1mol%) and dtbbpy (1mol%) in MeCN (typically 50mL, concentration ~0.45M). The reaction solution was subjected to one freeze-pump-thaw cycle. The alcohol (1.6 mmol) was added the solution and then two further freeze-pump-thaw cycles were applied. The mixtures were transferred and pumped through a flow reactor fit with a blue LED light source at a controlled temperature. Samples of product were collected and analysed using gas chromatograph and/or NMR.

General Purification Procedure: Crude reaction mixture was diluted with EtOAc and washed with H_2O. The aqueous layer was further extract twice with EtOAc. The organic layers were combined and washed with, dried over MgSO4 and combined, rotary evaporated give dry crude product and purified by using automatic column chromatography (95:5 → 80:20 pentane/EtOAc) to afford the desired product.

1b. ^{1}H NMR (400 MHz, $CDCl_3$) δ 7.94 (d, *J* = 8.8 Hz, 2H), 6.93 (d, *J* = 8.8 Hz, 2H), 3.87 (s, 3H), 2.55 (s, 3H). ^{13}C NMR (101 MHz, $CDCl_3$) δ 196.92, 163.61, 130.72, 130.48, 113.81, 55.60, 26.48.

1c. ^{1}H NMR (400 MHz, $CDCl_3$) δ 7.93 (d, *J* = 8.8 Hz, 2H), 6.92 (d, *J* = 8.8 Hz, 2H), 4.10 (q, *J* = 7.0 Hz, 2H), 2.55 (s, 3H), 1.44 (d, *J* = 14.0 Hz, 3H).

1d. ^{1}H NMR (400 MHz, $CDCl_3$) δ 7.92 (d, *J* = 8.8 Hz, 2H), 6.91 (d, *J* = 8.8 Hz, 2H), 4.02 (t, *J* = 6.5 Hz, 2H), 2.55 (s, 3H), 1.78 (dt, *J* = 14.5, 6.6 Hz, 2H), 1.50 (h, *J* = 7.4 Hz, 2H), 0.98 (t, *J* = 7.4 Hz, 3H). ^{13}C NMR (101 MHz, $CDCl_3$) δ 196.94, 163.26, 130.70, 130.23, 114.25, 68.07, 31.26, 26.45, 19.31, 13.93.

1e. ^{1}H NMR (400 MHz, $CDCl_3$) δ 7.97 (d, *J* = 8.8 Hz, 2H), 6.99 (d, *J* = 8.8 Hz, 2H), 4.42 (q, *J* = 8.0 Hz, 2H), 2.57 (s, 3H). ^{13}C NMR (101 MHz, $CDCl_3$) δ 196.72, 160.96, 132.04, 130.85, 114.61, 65.86, 65.50, 65.15, 26.58.

1f. ^{1}H NMR (400 MHz, $CDCl_3$) δ 7.92 (d, *J* = 8.9 Hz, 2H), 6.94 (d, *J* = 8.8 Hz, 2H), 6.05 (ddt, *J* = 15.8, 10.5, 5.3 Hz, 1H), 5.42 (d, *J* = 17.3 Hz, 1H), 5.32 (d, *J* = 10.5 Hz, 1H), 4.60 (d, *J* = 5.2 Hz, 2H), 2.55 (s, 3H). ^{13}C NMR (101 MHz, $CDCl_3$) δ 196.90, 162.60, 132.62, 130.70, 130.56, 118.33, 114.52, 69.01, 26.47.

1g. ^{1}H NMR (400 MHz, $CDCl_3$) δ 8.00 – 7.91 (m, 2H), 7.00 – 6.92 (m, 2H), 4.18 (t, *J* = 7.0 Hz, 2H), 2.73 (td, *J* = 7.0, 2.7 Hz, 2H), 2.58 (s, 3H), 2.08 (t, *J* = 2.7 Hz, 1H). ^{13}C NMR (101 MHz, $CDCl_3$) δ 196.75, 162.31, 130.67, 130.62, 114.24, 79.98, 70.18, 66.09, 26.37, 19.47.

1h. ^{1}H NMR (400 MHz, $CDCl_3$) δ 7.92 (d, *J* = 8.8 Hz, 2H), 6.92 (d, *J* = 8.8 Hz, 2H), 3.90 (d, *J* = 5.7 Hz, 2H), 2.55 (s, 3H), 1.74 (dt, *J* = 12.2, 6.1 Hz, 1H), 1.55 – 1.24 (m, 8H), 1.04 – 0.83 (m, 6H). ^{13}C NMR (101 MHz, $CDCl_3$) δ 196.94, 163.51, 132.03, 130.69, 130.19, 114.29, 70.86, 39.41, 30.59, 29.18, 26.46, 23.94, 23.15, 14.20, 11.22.

1i. ^{1}H NMR (400 MHz, $CDCl_3$) δ 7.94 (d, *J* = 8.8 Hz, 2H), 7.46 – 7.33 (m, 7H), 7.01 (d, *J* = 8.8 Hz, 2H), 5.14 (s, 2H), 2.56 (s, 3H). ^{13}C NMR (101 MHz, $CDCl_3$) δ 196.93, 162.76, 136.31, 130.75, 130.67, 128.84, 128.39, 127.61, 114.68, 70.28, 26.50.

1j. ^{1}H NMR (400 MHz, $CDCl_3$) δ 7.92 (d, *J* = 8.8 Hz, 2H), 7.31 (dt, *J* = 15.3, 7.7 Hz, 5H), 7.24 (s, 1H), 6.92 (d, *J* = 8.8 Hz, 2H), 4.24 (t, *J* = 7.1 Hz, 2H), 3.13 (t, *J* = 7.0 Hz, 2H), 2.55 (s, 3H). ^{13}C NMR (101 MHz, $CDCl_3$) δ 196.93, 162.88, 137.94, 130.74, 130.49, 129.14, 128.72, 126.82, 114.34, 69.01, 35.77, 26.49.

1k. ^{1}H NMR (400 MHz, $CDCl_3$) δ 7.98 – 7.90 (m, 2H), 7.00 – 6.92 (m, 2H), 4.51 (dq, *J* = 6.5, 5.7 Hz, 1H), 4.19 (dd, *J* = 8.5, 6.4 Hz, 1H), 4.12 (dd, *J* = 9.5, 5.5 Hz, 1H), 4.02 (dd, *J* = 9.6, 5.7 Hz, 1H), 3.92 (dd, *J* = 8.5, 5.8 Hz, 1H), 2.56 (s, 3H), 1.48 (s, 3H), 1.42 (d, *J* = 0.8 Hz, 3H). ^{13}C NMR (101 MHz, $CDCl_3$) δ 196.71, 162.40, 130.71, 130.58, 130.55, 114.22, 109.93, 73.82, 68.87, 66.67, 26.78, 26.36, 25.33.

1l. ^{1}H NMR (400 MHz, $CDCl_3$) δ 7.94 (d, *J* = 8.8 Hz, 2H), 7.46 (s, 1H), 7.02 (d, *J* = 8.8 Hz, 2H), 6.47 (s, 1H), 6.44 – 6.37 (m, 1H), 5.07 (s, 2H), 2.56 (s, 3H).

1m. ^{1}H NMR (400 MHz, $CDCl_3$) δ 7.92 (d, *J* = 8.7 Hz, 2H), 6.90 (d, *J* = 8.7 Hz, 2H), 4.65 (hept, *J* = 6.0 Hz, 1H), 2.55 (s, 3H), 1.37 (d, *J* = 6.0 Hz, 6H). ^{13}C NMR (101 MHz, $CDCl_3$) δ 196.92, 162.15, 130.77, 130.07, 115.20, 70.23, 26.46, 22.06.

1n. ^{1}H NMR (400 MHz, $CDCl_3$) δ 7.91 (d, *J* = 8.7 Hz, 2H), 6.90 (d, *J* = 8.7 Hz, 2H), 4.40 (h, *J* = 5.9 Hz, 1H), 2.54 (s, 3H), 1.85 – 1.58 (m, 3H), 1.32 (d, *J* = 6.1 Hz, 3H), 0.98 (t, *J* = 7.4 Hz, 3H). ^{13}C NMR (101 MHz, $CDCl_3$) δ 196.89, 162.49, 130.76, 130.01, 115.19, 75.31, 29.21, 26.44, 19.25, 9.83.

1o. ^{1}H NMR (400 MHz, $CDCl_3$) δ 7.91 (d, *J* = 8.7 Hz, 2H), 6.89 (d, *J* = 8.7 Hz, 2H), 4.93 – 4.73 (m, 1H), 2.55 (s, 3H), 2.06 – 1.60 (m, 8H). ^{13}C NMR (101 MHz, $CDCl_3$) δ 196.95, 162.36, 130.68, 129.95, 115.19, 79.78, 33.00, 26.46, 24.19.

1p. ^{1}H NMR (400 MHz, $CDCl_3$) δ 7.91 (d, *J* = 8.8 Hz, 2H), 6.91 (d, *J* = 8.8 Hz, 2H), 4.35 (tt, *J* = 8.6, 3.7 Hz, 1H), 2.55 (s, 3H), 2.08 – 1.93 (m, 2H), 1.90 – 1.75 (m, 2H), 1.64 – 1.57 (m, 2H), 1.53 (d, *J* = 12.7 Hz, 1H), 1.39 (ddt, *J* = 18.6, 8.7, 4.7 Hz, 3H). ^{13}C NMR (101 MHz, $CDCl_3$) δ 196.91, 162.10, 130.75, 130.04, 115.31, 75.59, 31.74, 26.46, 25.65, 23.78.

1q. ^{1}H NMR (400 MHz, CD_3CN) δ 7.49 (d, *J* = 8.7 Hz, 2H), 7.43 – 7.31 (m, 4H), 7.31 – 7.23 (m, 1H), 7.00 (d, *J* = 8.6 Hz, 2H), 5.32 (dd, *J* = 8.6, 4.2 Hz, 1H), 3.42 (s, 2H), 2.80 (s, 3H), 2.22 – 2.00 (m, 3H), 1.33 (s, 9H). ^{13}C NMR (101 MHz, CD_3CN) δ 160.77, 141.09, 128.71, 127.88, 126.78, 126.74, 126.70, 126.66, 126.04, 121.87, 116.16, 78.65, 45.17, 27.54.

1r. ^{1}H NMR (400 MHz, $CDCl_3$) δ 7.16 – 7.09 (m, 2H), 6.93 – 6.85 (m, 2H), 4.43 (tt, *J* = 7.1, 3.5 Hz, 1H), 3.69 (ddd, *J* = 13.4, 7.6, 3.8 Hz, 2H), 3.34 (ddd, *J* = 13.6, 7.7, 3.8 Hz, 2H), 2.16 (s, 1H), 1.97 – 1.85 (m, 2H), 1.80 – 1.67 (m, 2H), 1.47 (s, 9H). ^{13}C NMR (101 MHz, $CDCl_3$) δ 155.73, 154.81, 142.87, 142.85, 142.83, 122.50, 121.83, 119.28, 116.86, 79.65, 72.77, 30.40, 28.41.

Recovered Catalyst Data

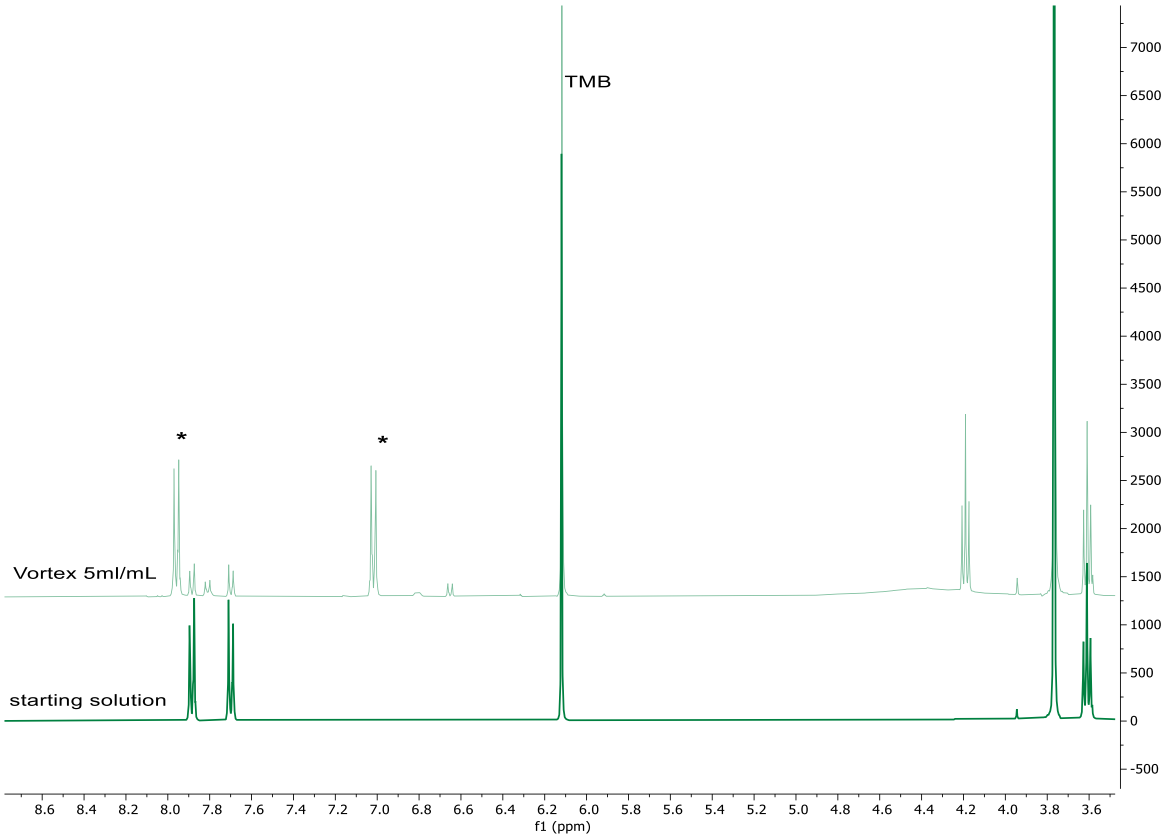

Figure S5. ^{1}H NMR of reaction performed using the recovered iridium catalyst in MeCN-*d3*. Spectrum were obtained with 50 µL of sample and Trimetoxybenzene (4.3 mg, TMB) was used as standard. Yield: 76% (83% conversion). Reaction conditions: 1 eq. aryl halide, 1.6 eq. alcohol, 1.1 eq base, 0.5 mol% [Ir{dF(CF_3)ppy}$_2$dtbbpy)]PF_6, 2.5 mol% $NiCl_2$-glyme, 2.5 mol% dtbbpy, MeCN, Vortex Reactor, 1.6 min space-time

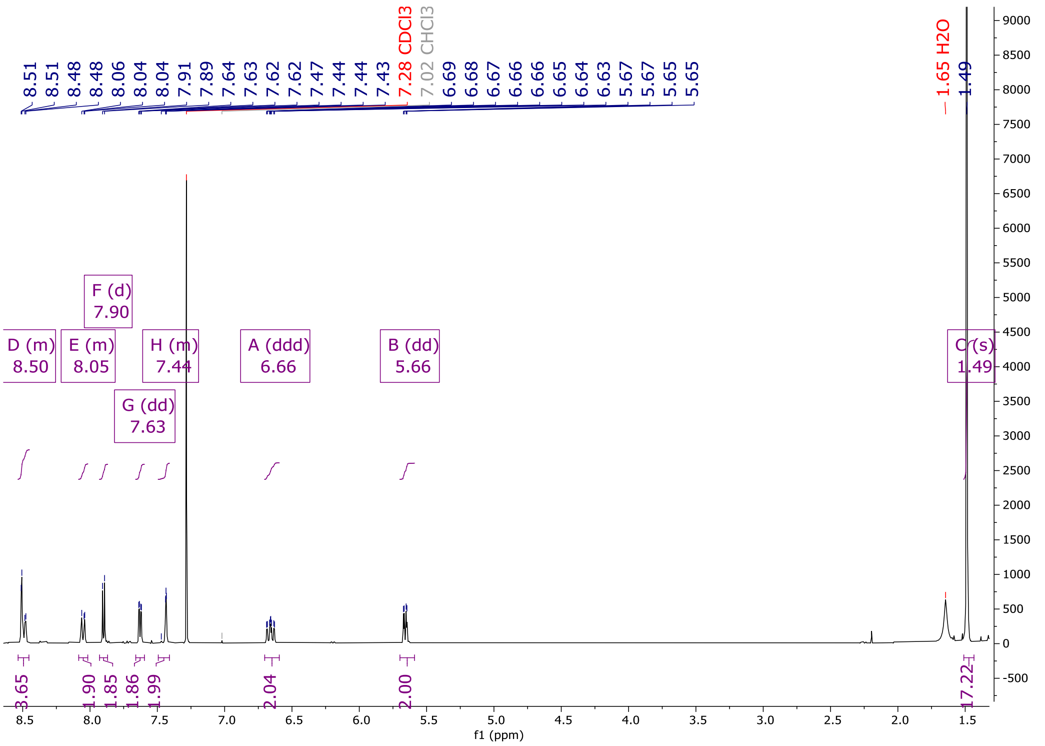

Figure S6. ^{1}H NMR of virgin iridium catalyst in $CDCl_3$.

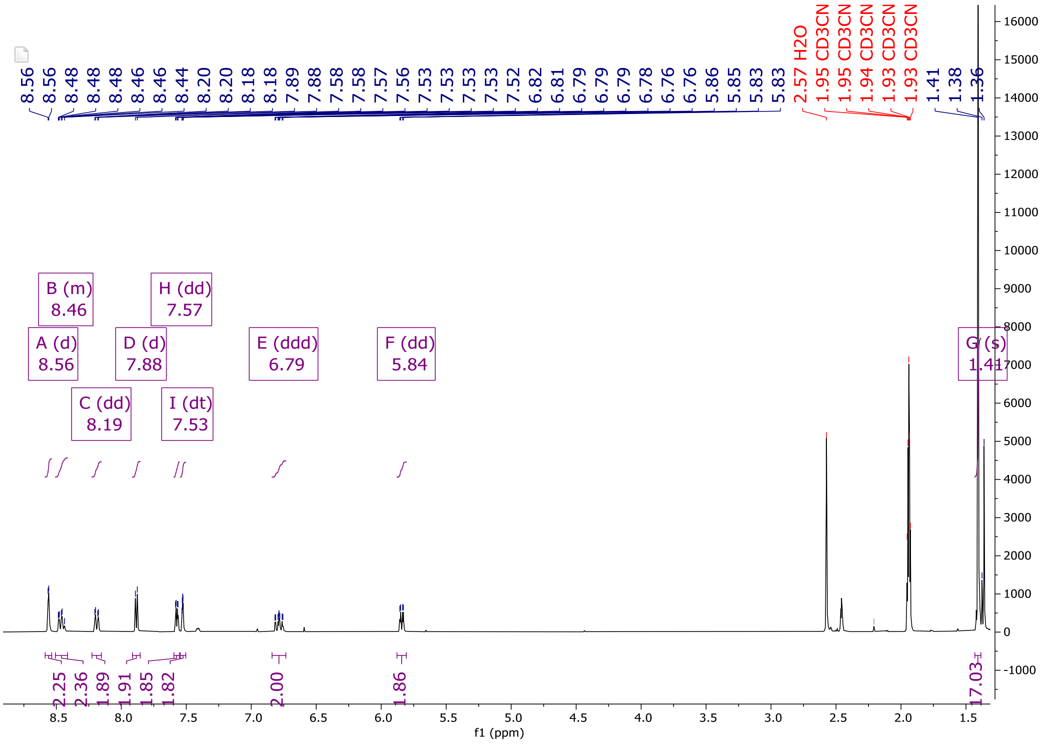

Figure S7. ^{1}H NMR of recovered iridium catalyst in MeCN-*d3* (with 10% DMSO-*d6*).

NMR Characterisation Data of Compounds

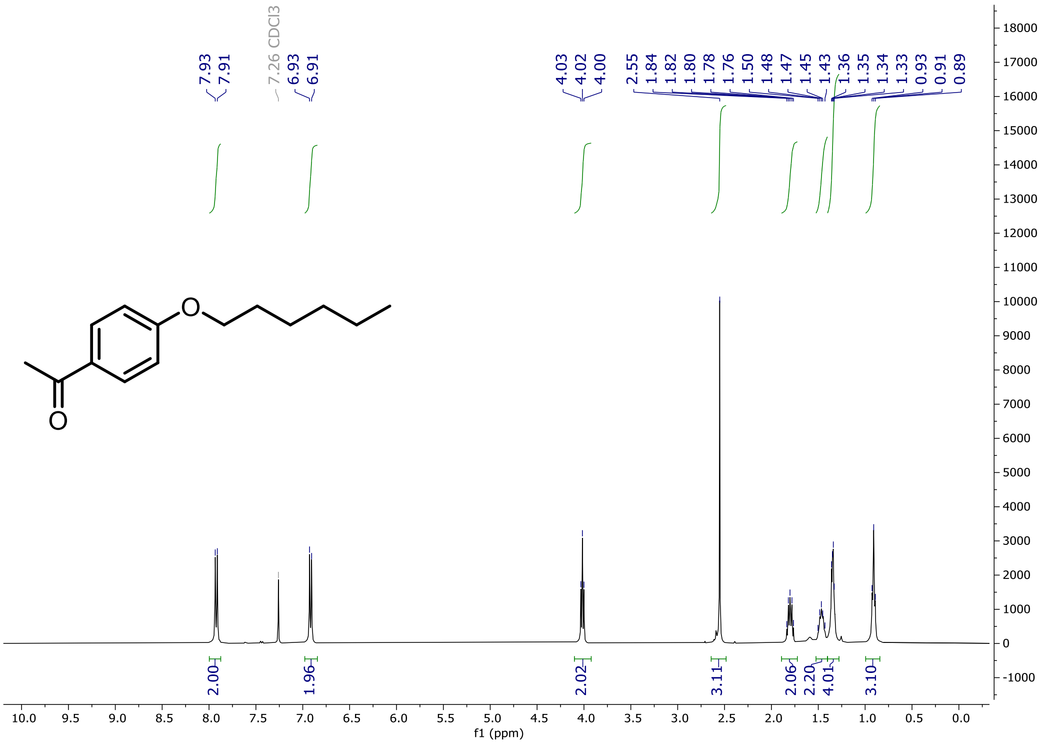

^{1}H NMR of **1a** in $CDCl_3$. Consistent with previous reports.[170]

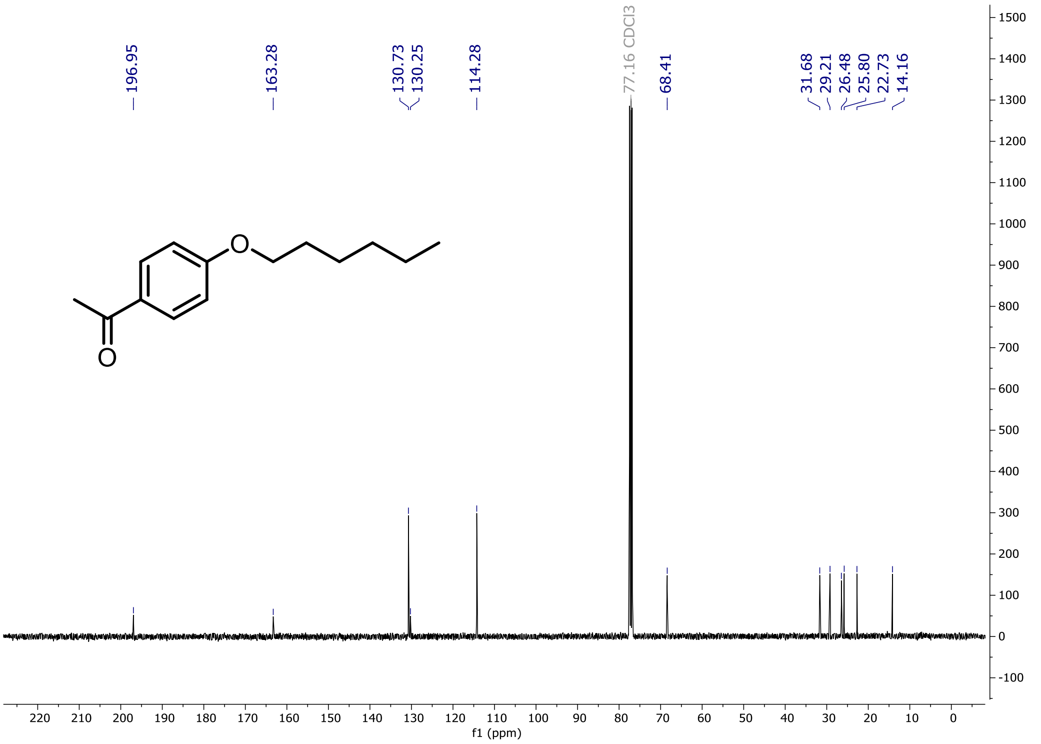

^{13}C NMR of **1a** in $CDCl_3$. Consistent with previous reports.[170]

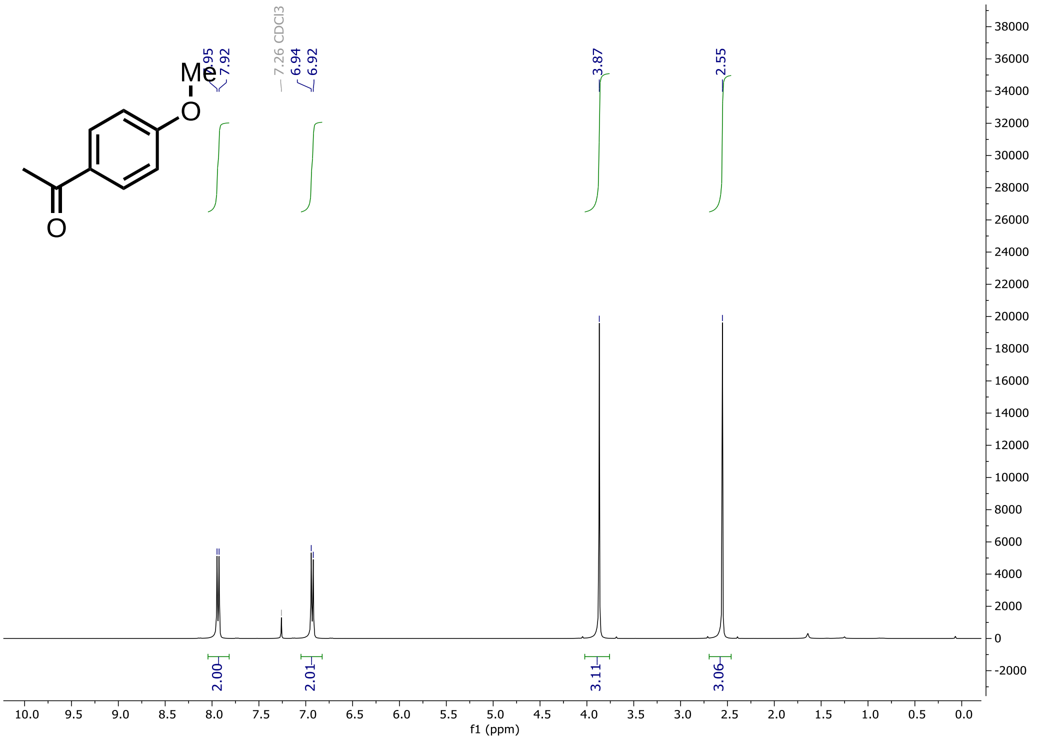

^{1}H NMR of **1b** in $CDCl_3$.

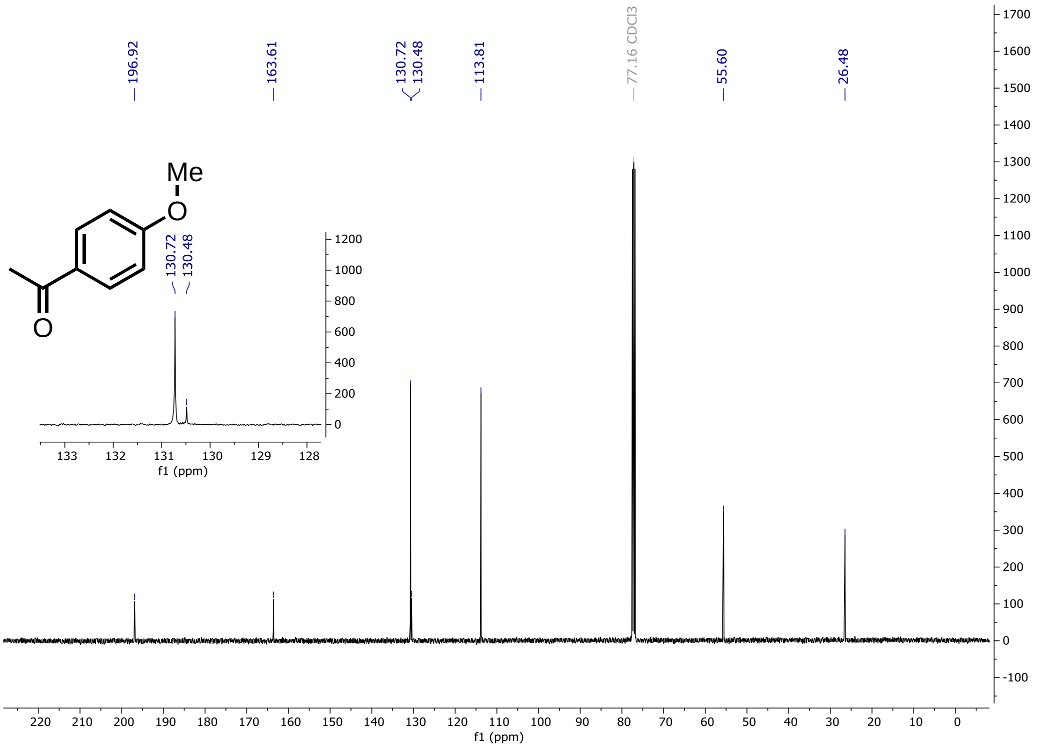

^{13}C NMR of **1b** in $CDCl_3$.

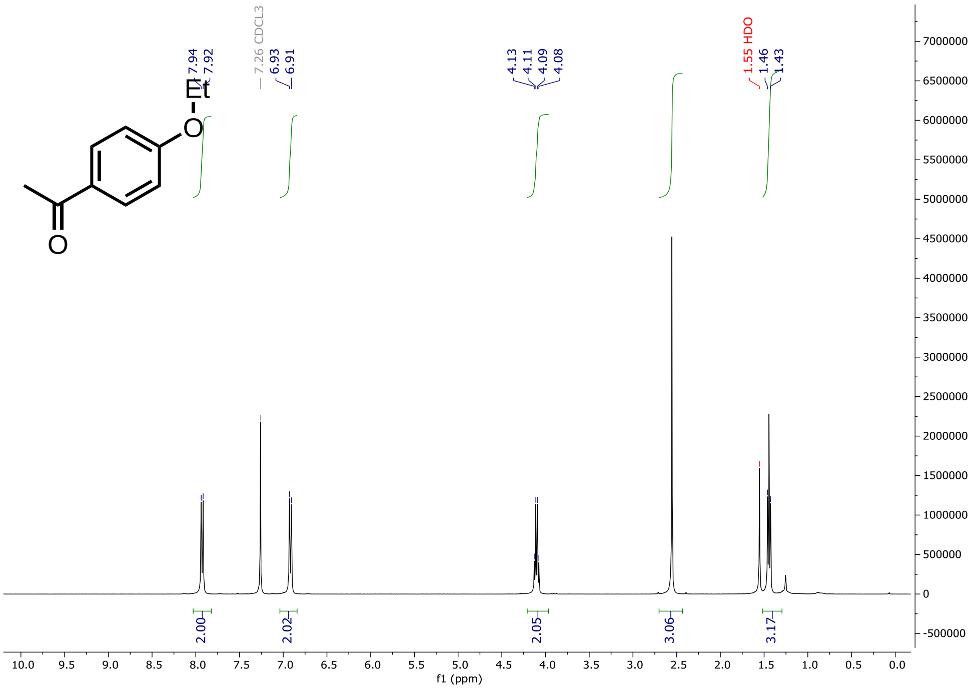

^{1}H NMR of **1c** in $CDCl_3$.

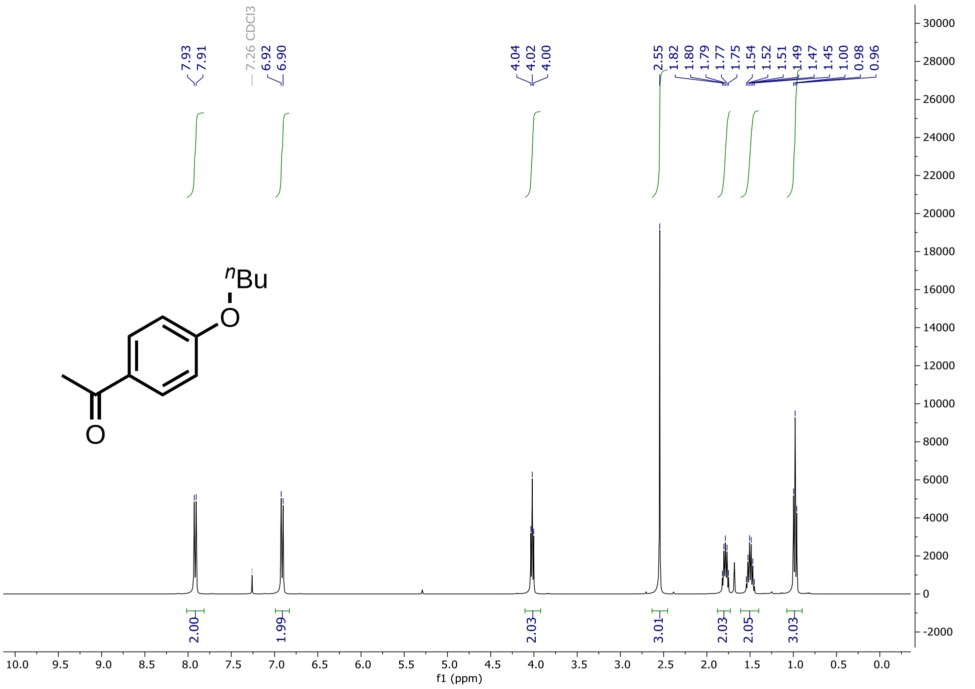

^{1}H NMR of **1d** in $CDCl_3$.

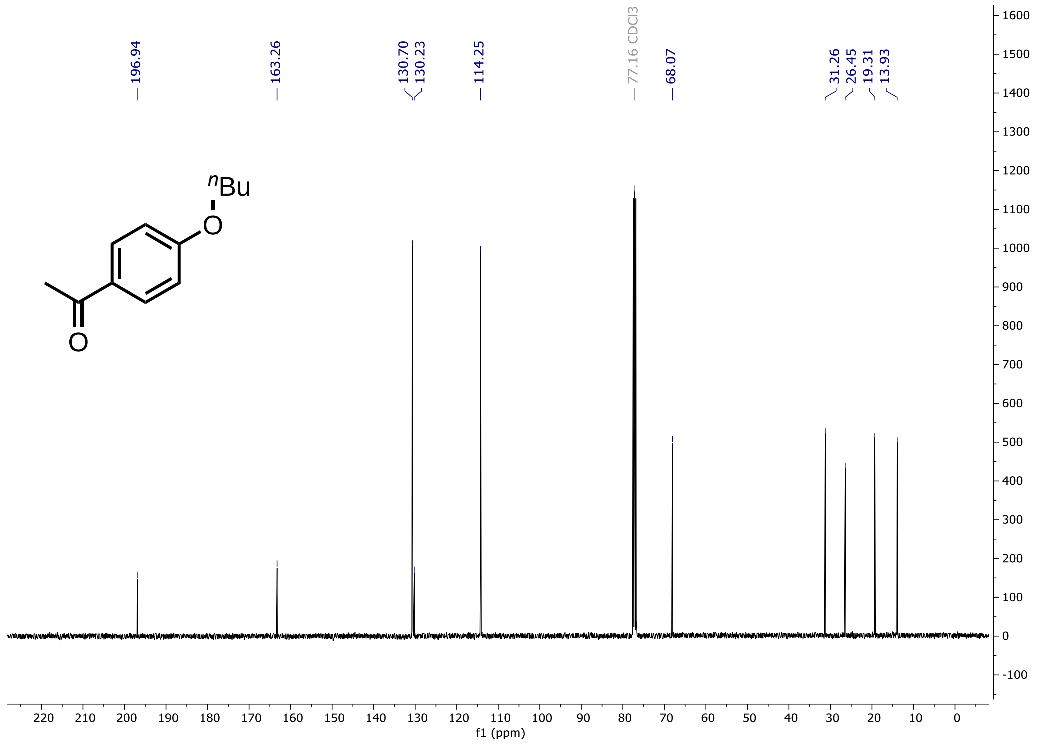

^{13}C NMR of **1d** in $CDCl_3$.

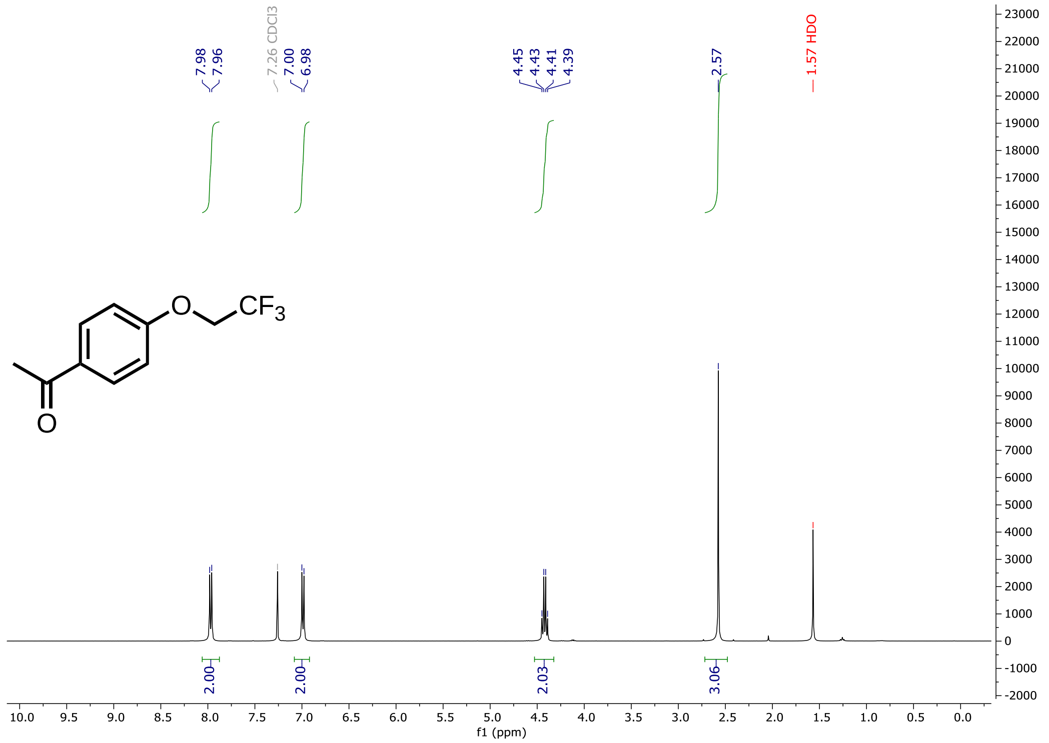

^{1}H NMR of **1e** in $CDCl_3$.

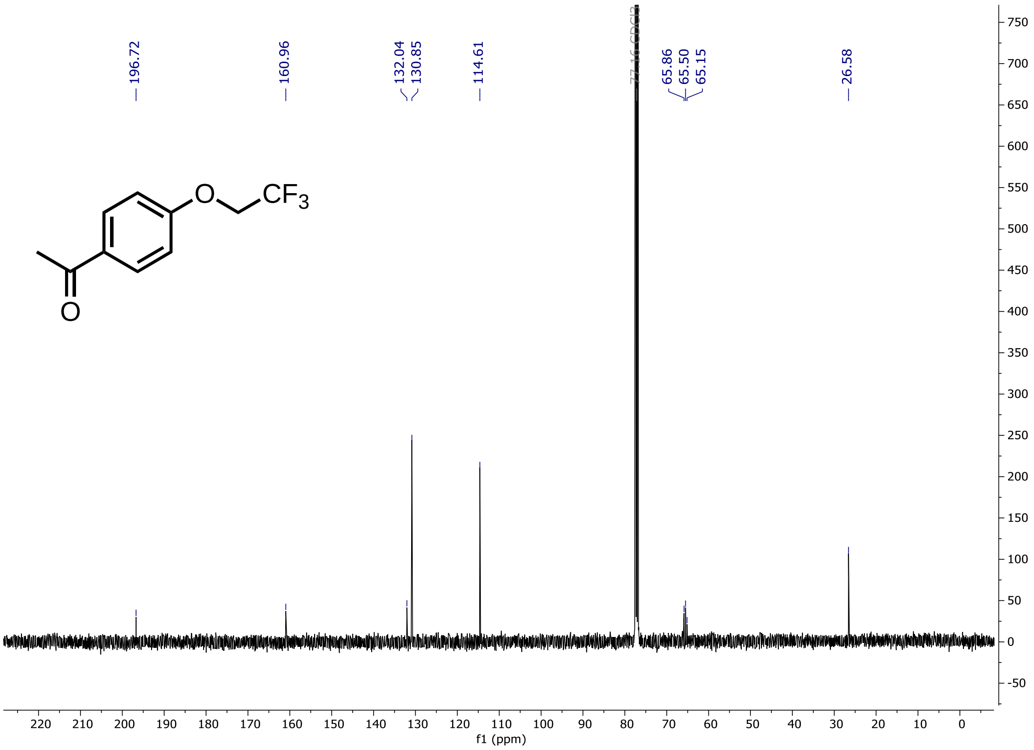

^{13}C NMR of **1e** in $CDCl_3$.

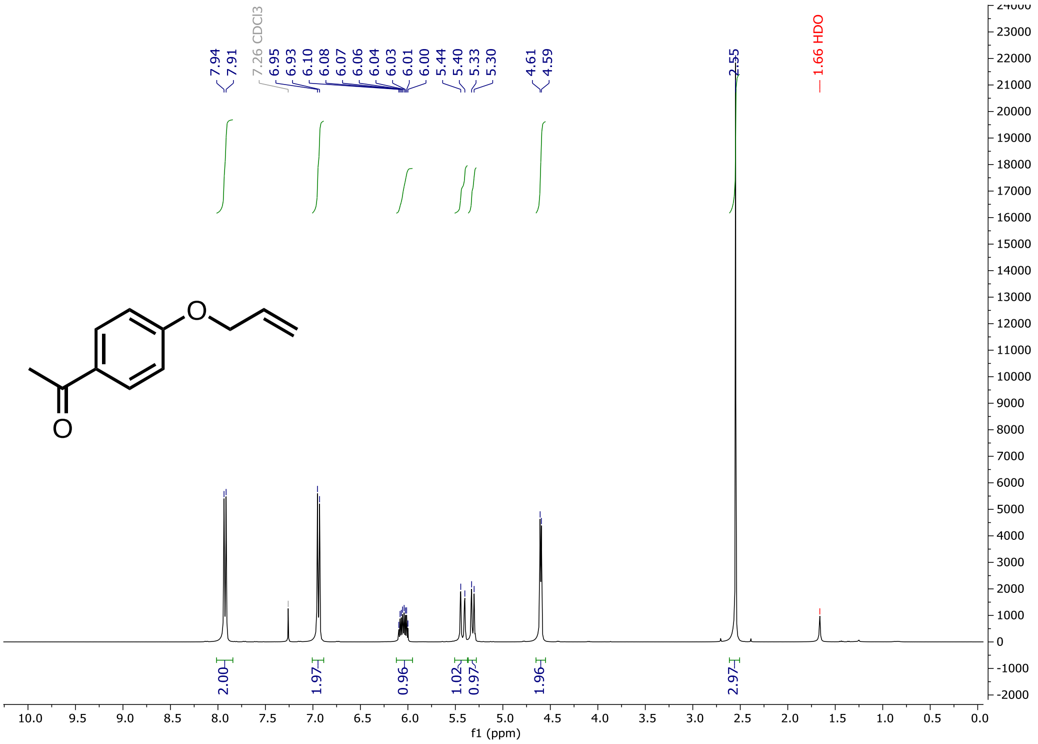

^{1}H NMR of **1f** in $CDCl_3$.

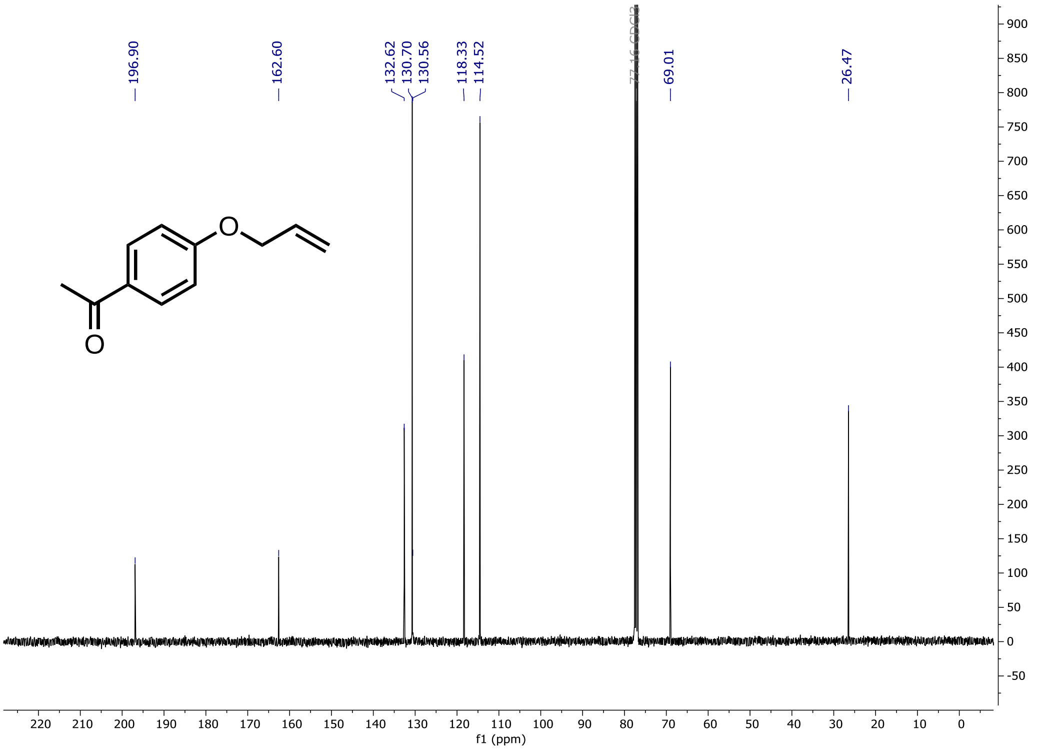

^{13}C NMR of **1f** in $CDCl_3$.

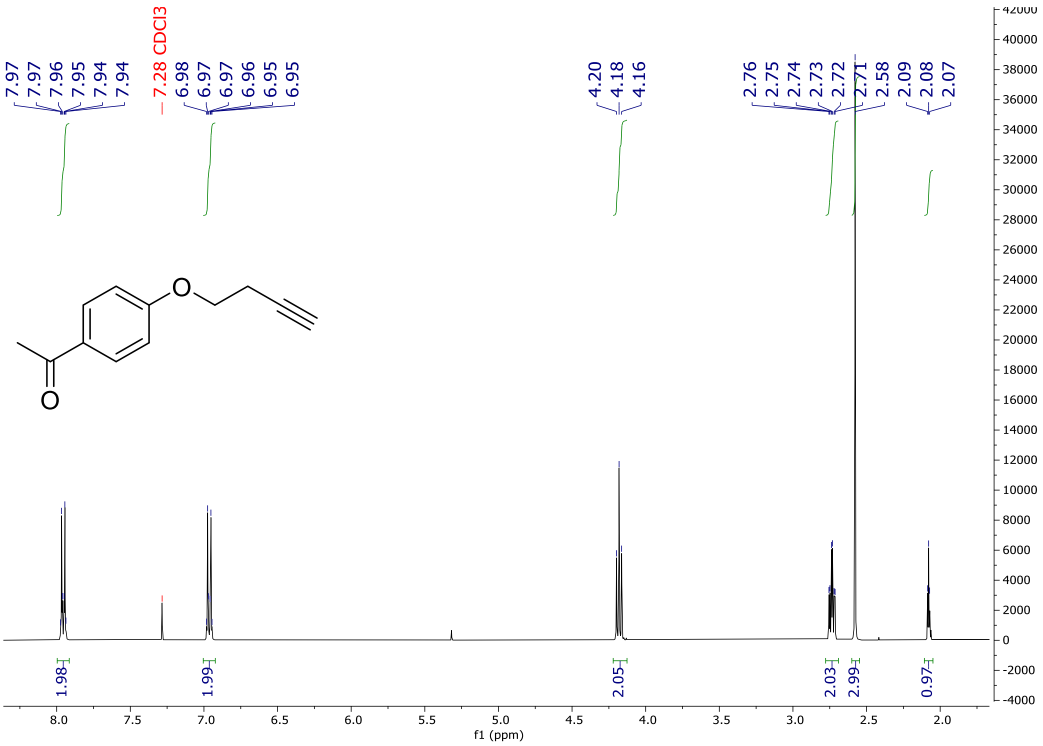

^{1}H NMR of **1g** in $CDCl_3$.

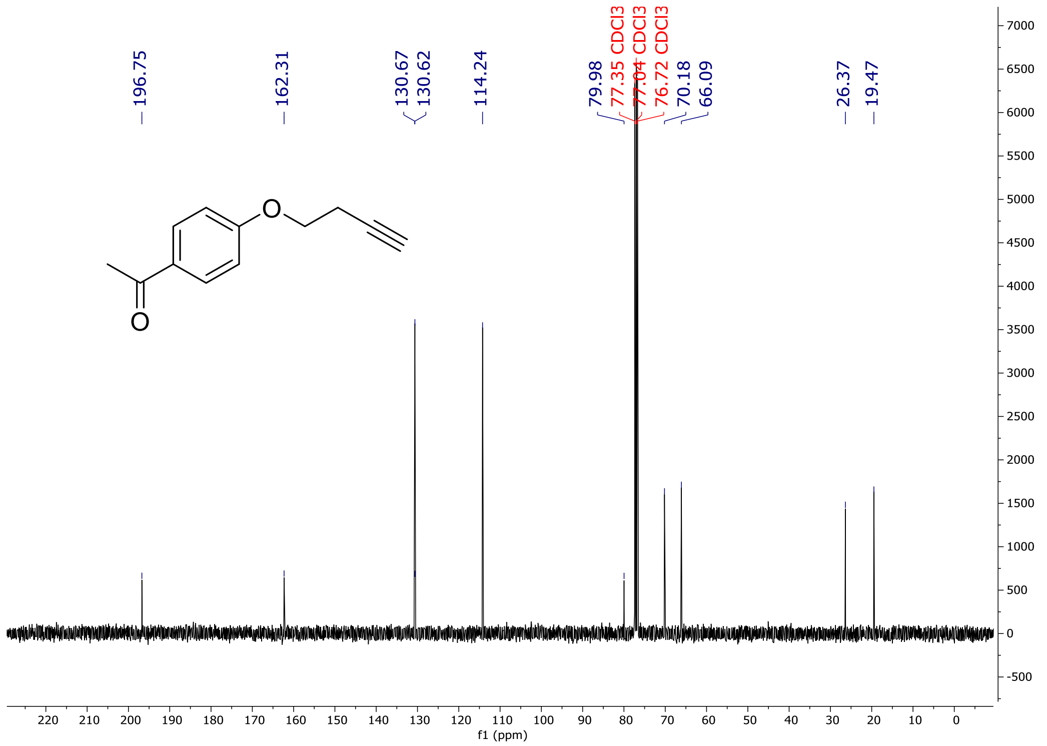

^{13}C NMR of **1g** in $CDCl_3$.

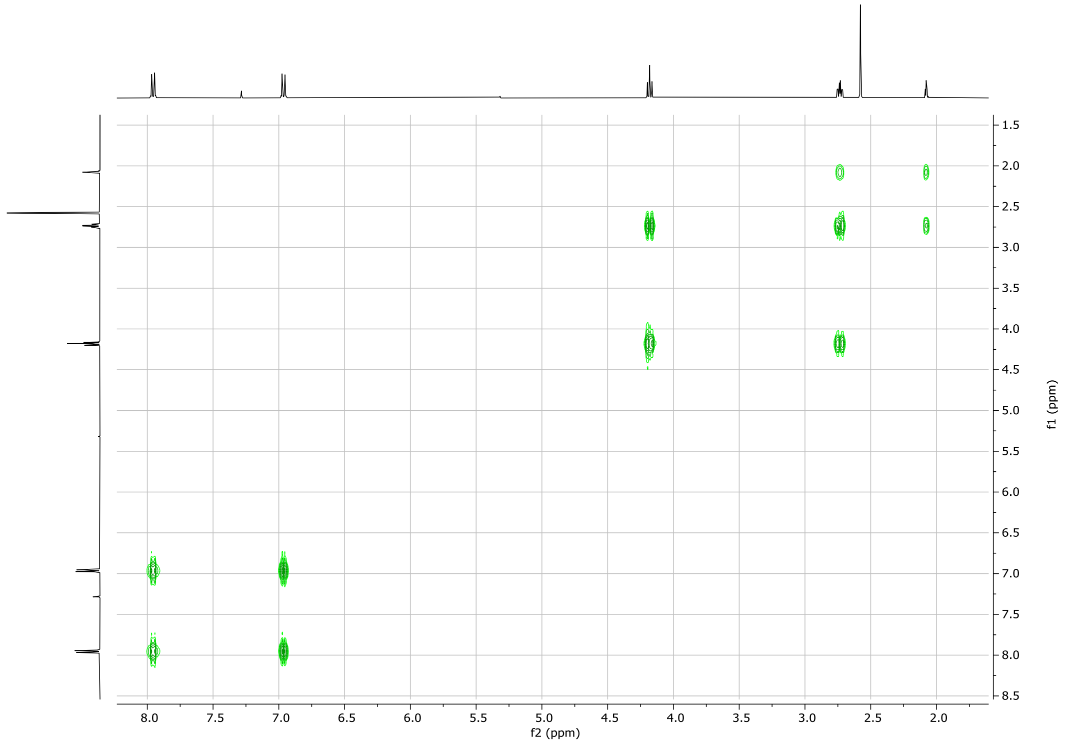

COSY NMR of **1g** in $CDCl_3$.

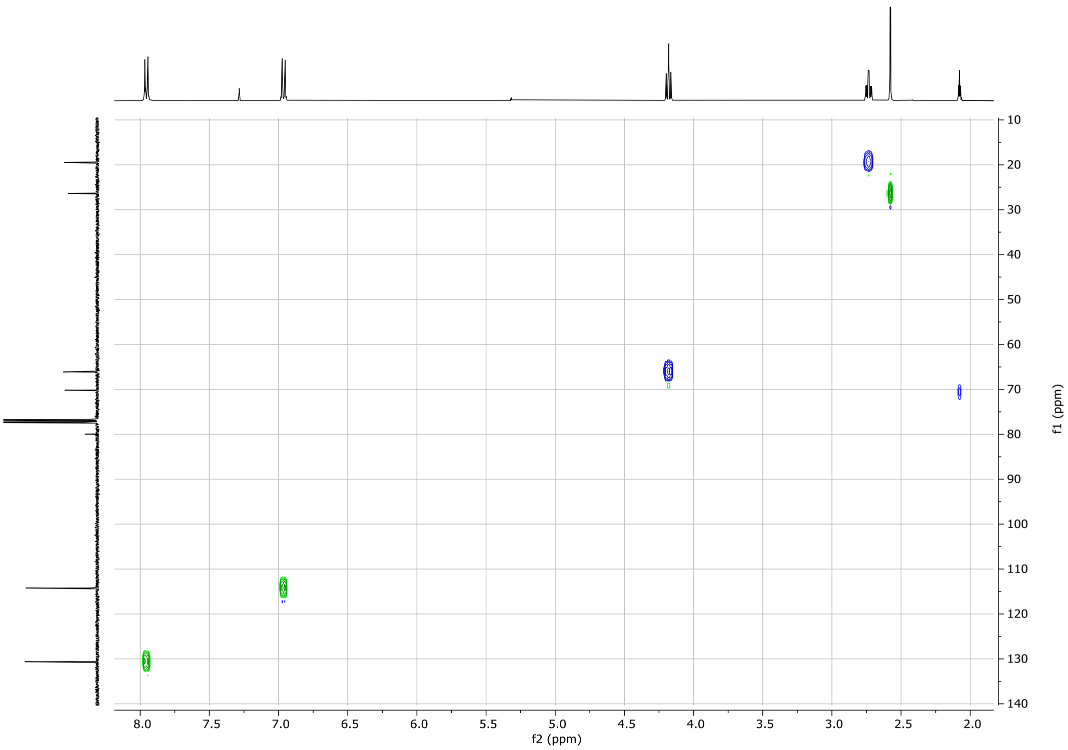

HSQC NMR of **1g** in $CDCl_3$.

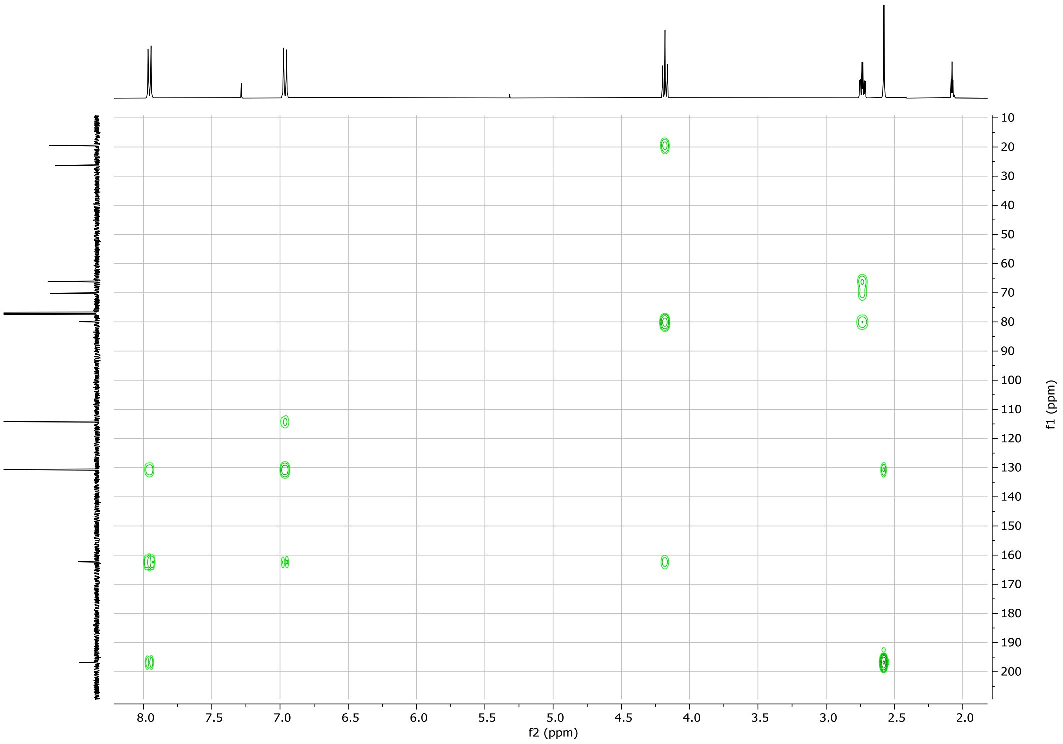

HMBC NMR of **1g** in $CDCl_3$.

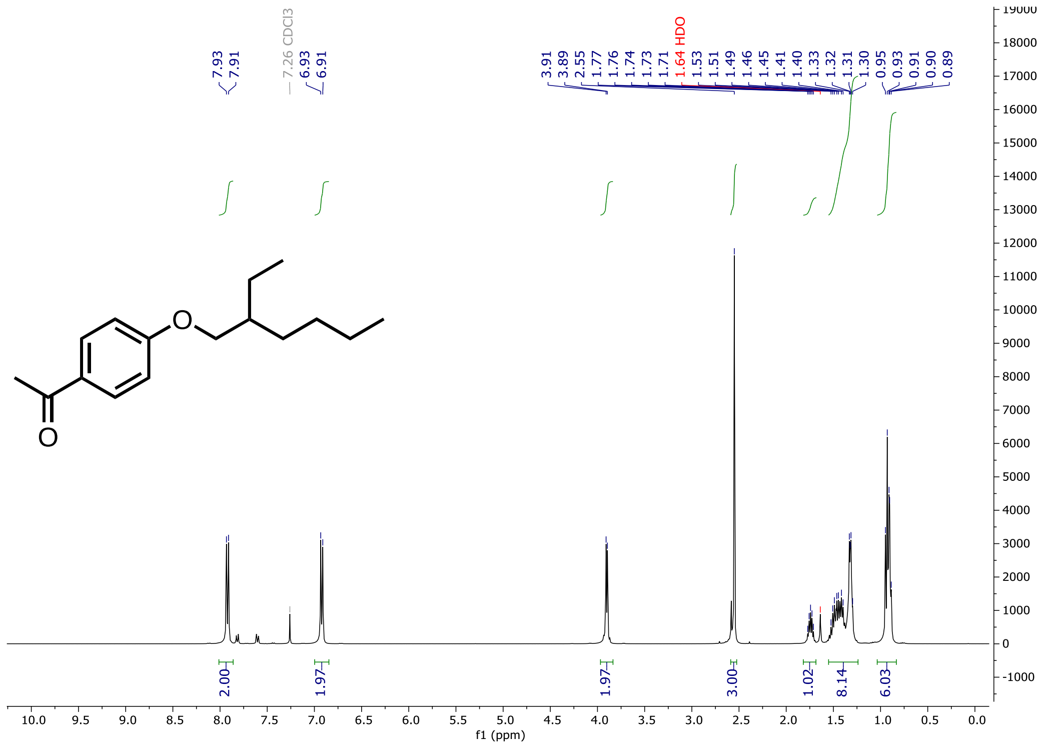

^{1}H NMR of **1h** in $CDCl_3$.

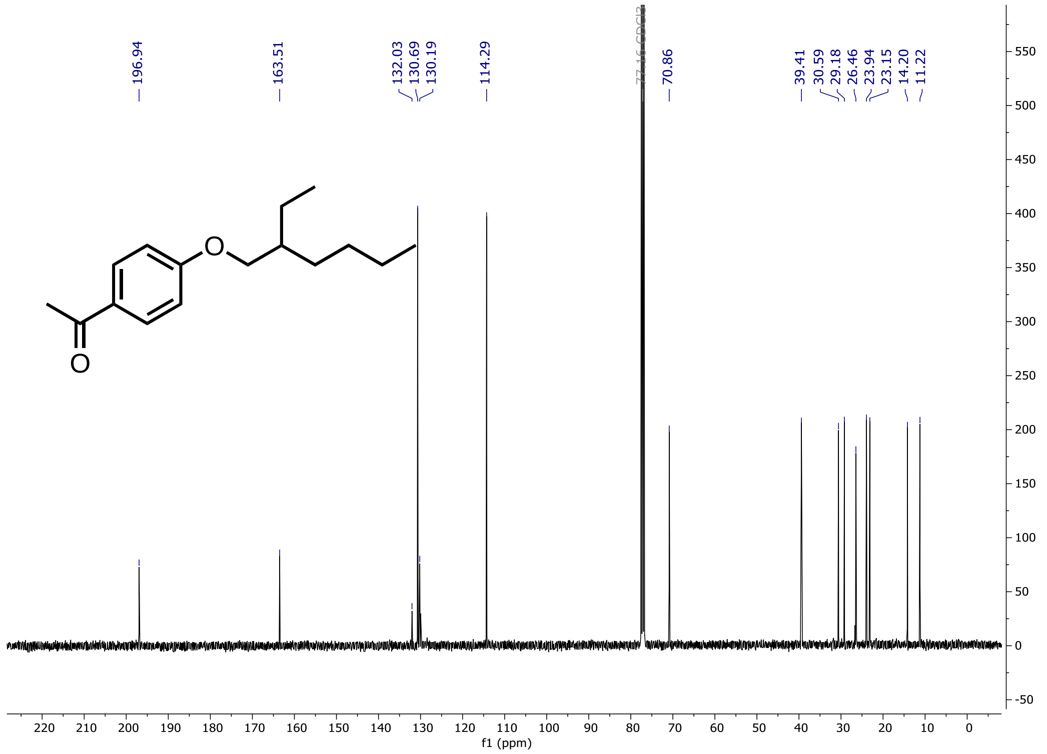

^{13}C NMR of **1h** in $CDCl_3$.

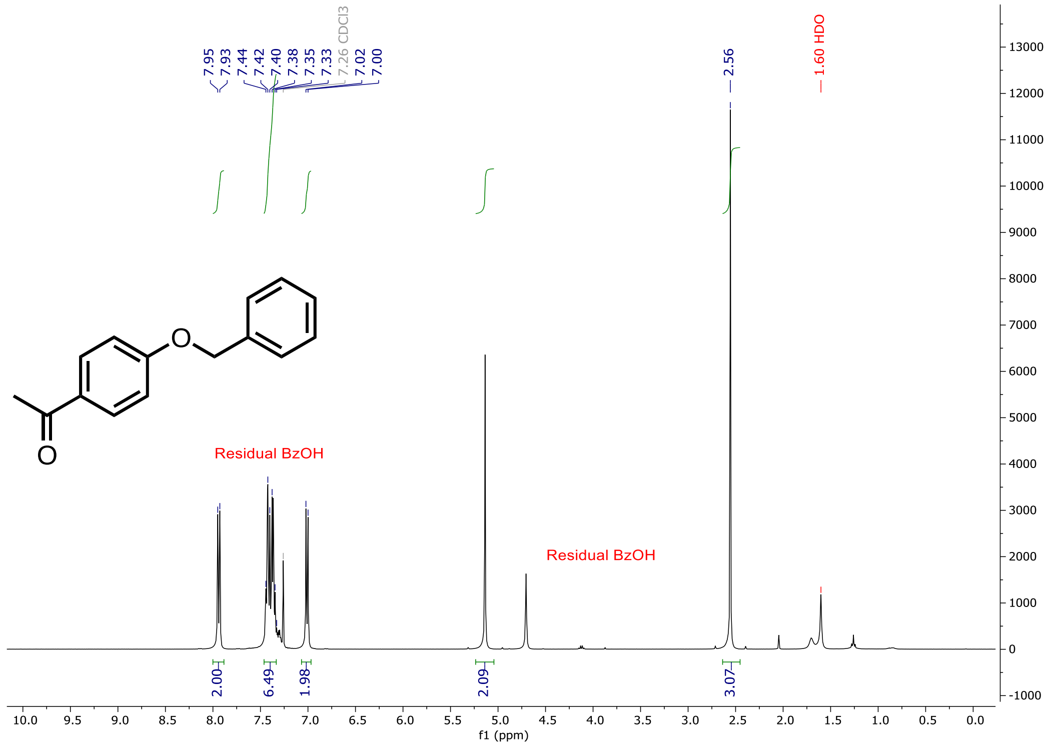

^{1}H NMR of **1i** in $CDCl_3$.

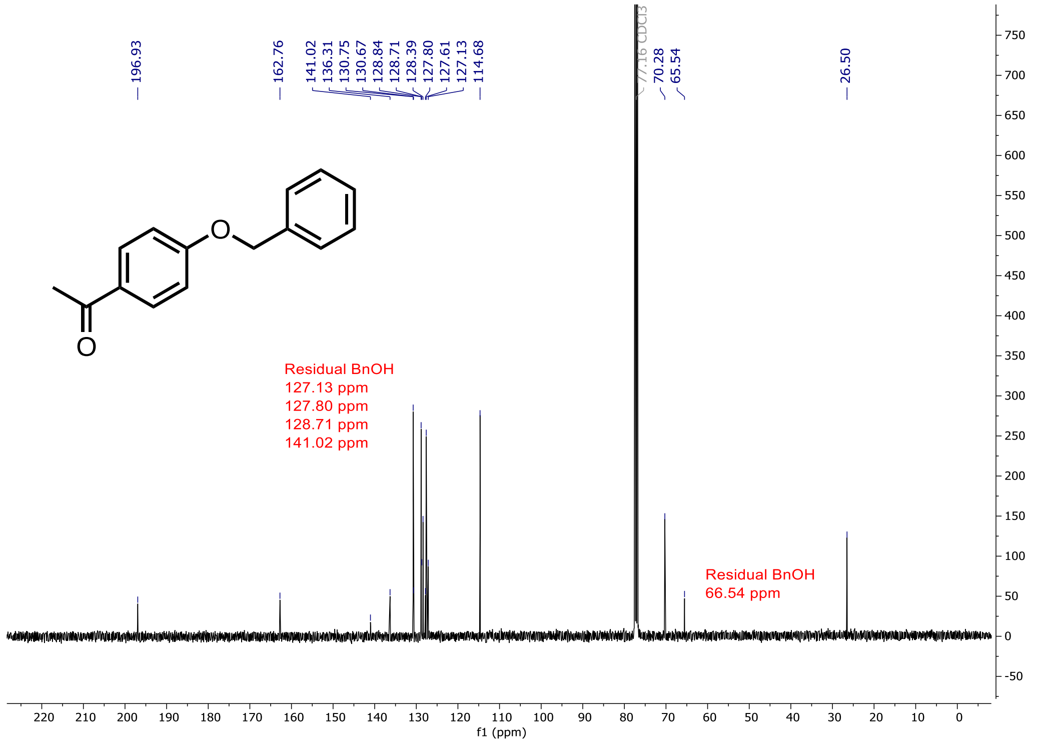

^{13}C NMR of **1i** in $CDCl_3$.

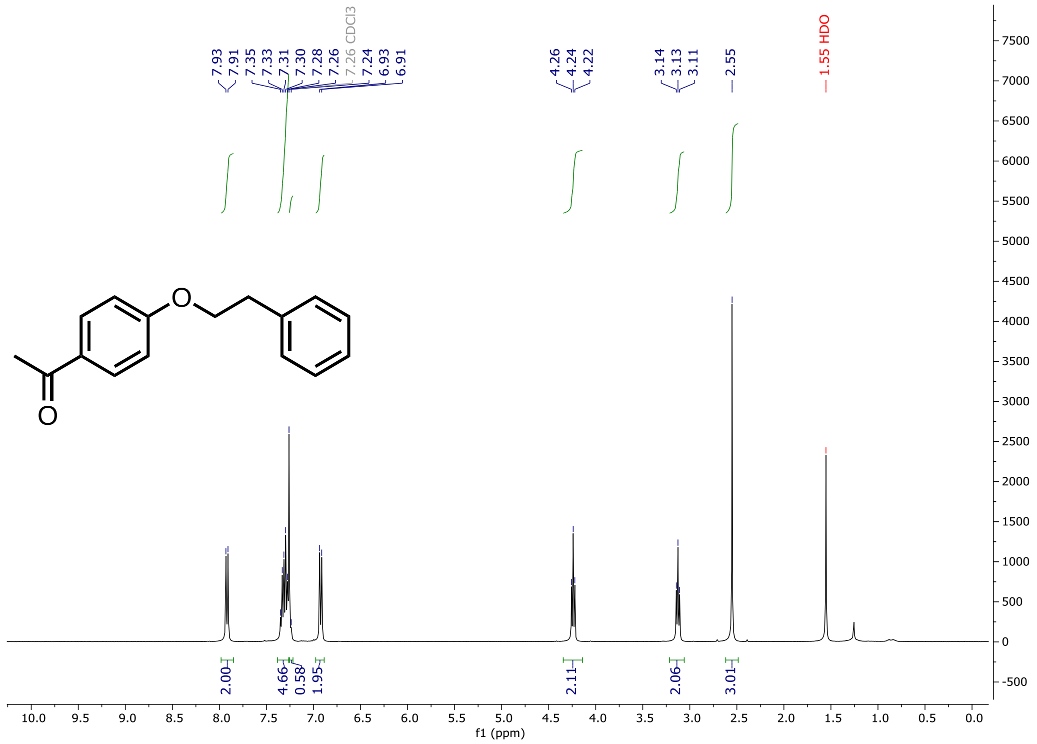

^{1}H NMR of **1j** in $CDCl_3$.

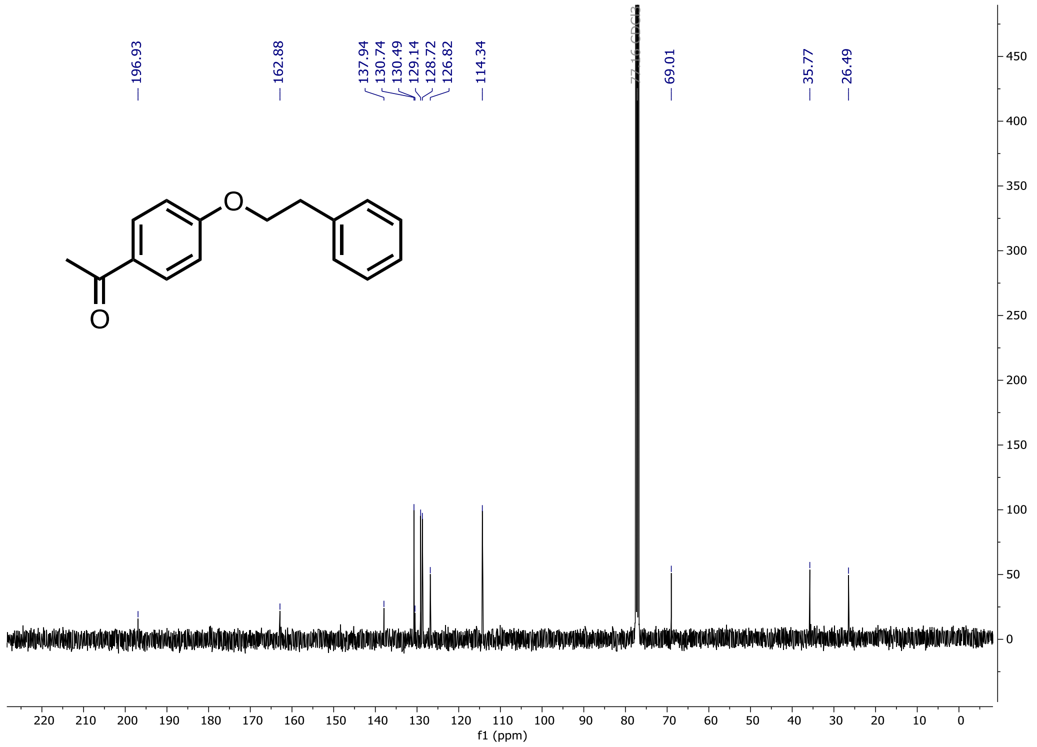

^{13}C NMR of **1j** in $CDCl_3$.

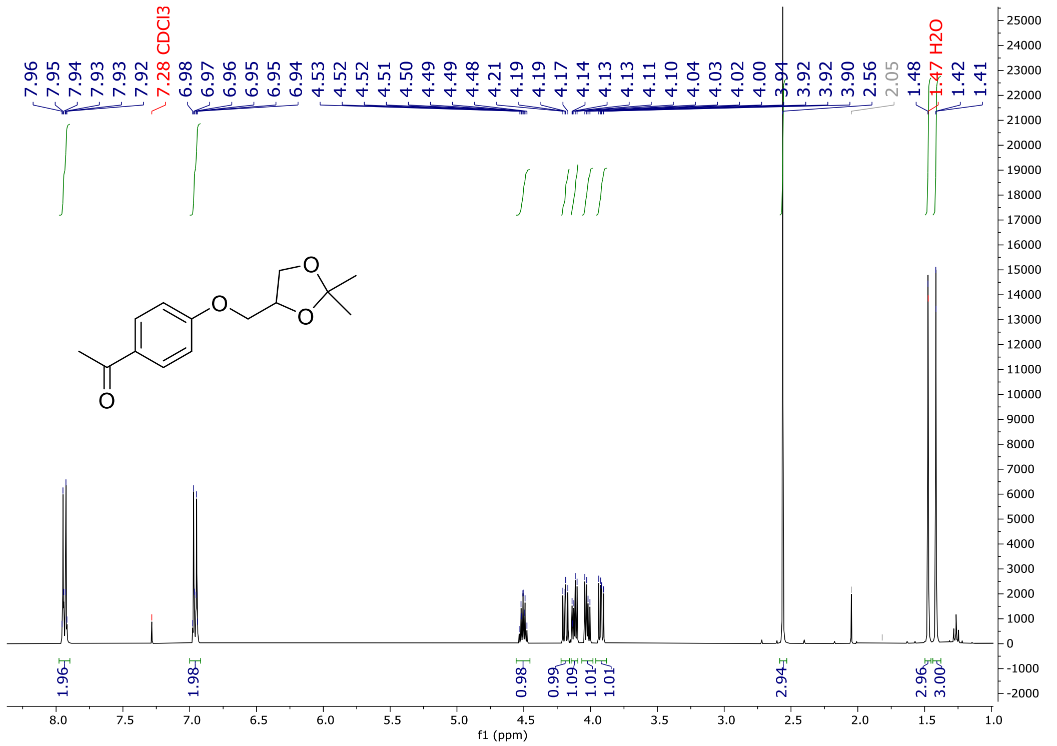

^{1}H NMR of **1k** in $CDCl_3$.

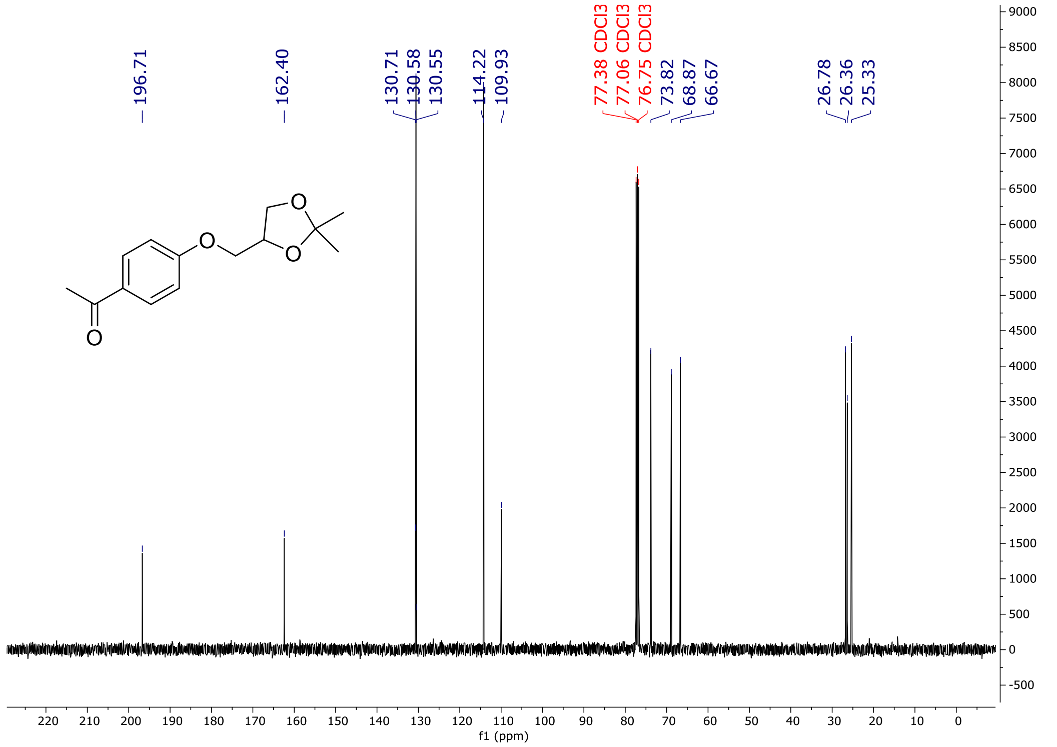

^{13}C NMR of **1k** in $CDCl_3$.

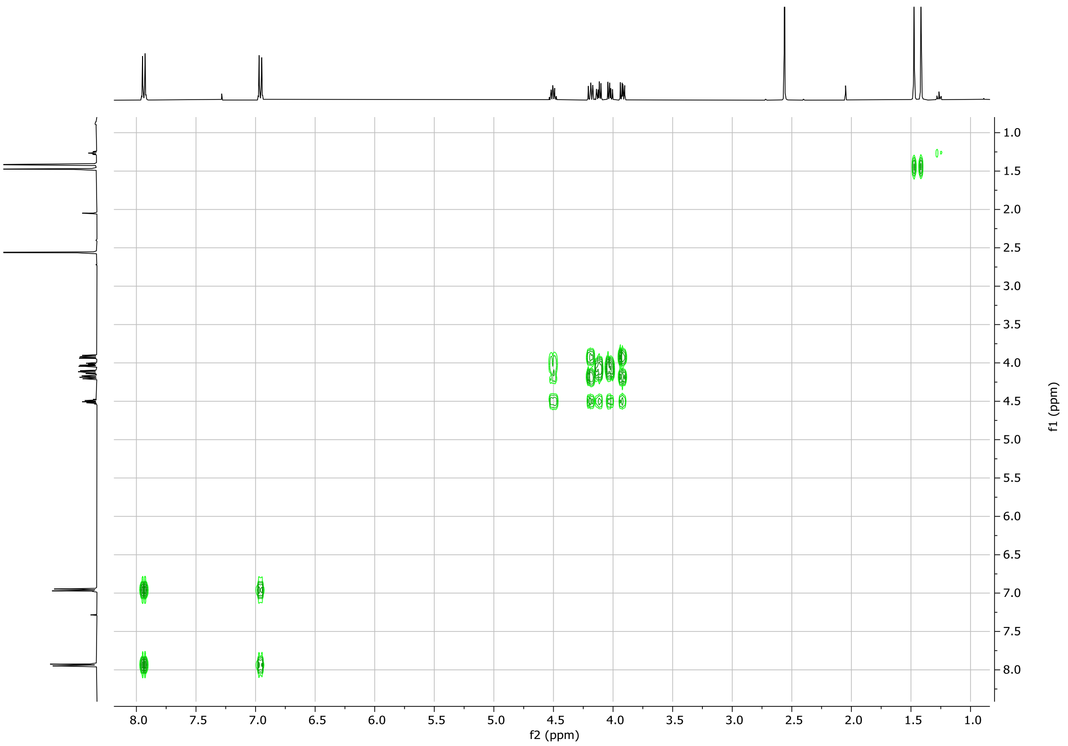

COSY NMR of **1k** in $CDCl_3$.

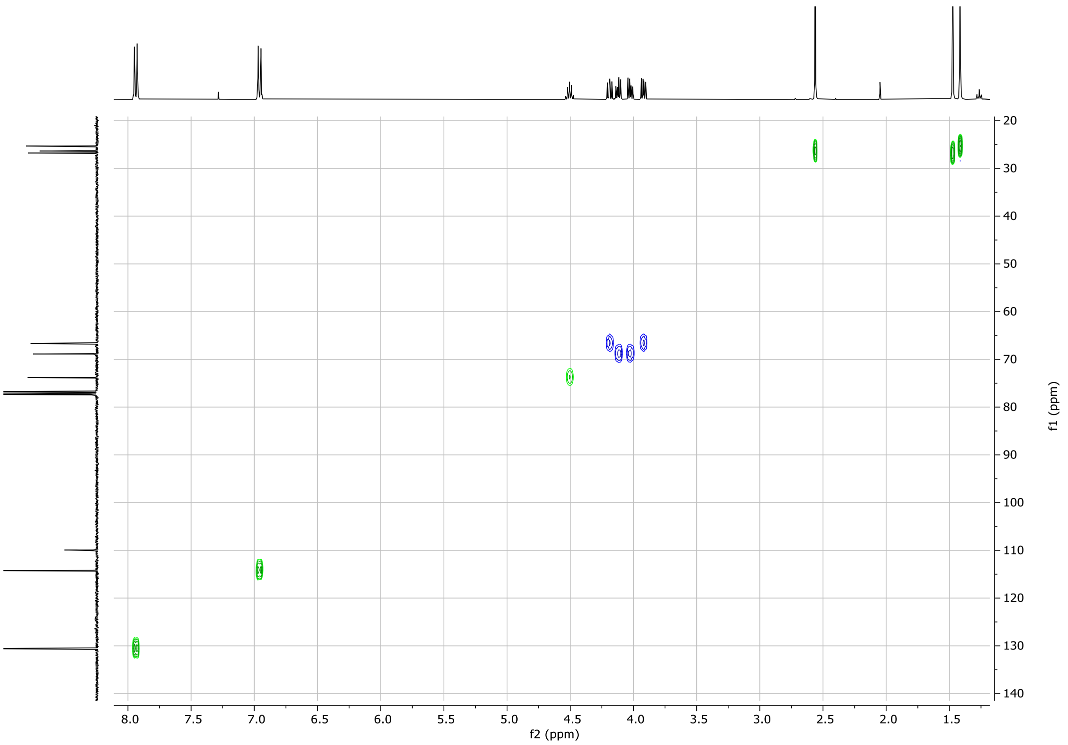

HSQC NMR of **1k** in $CDCl_3$.

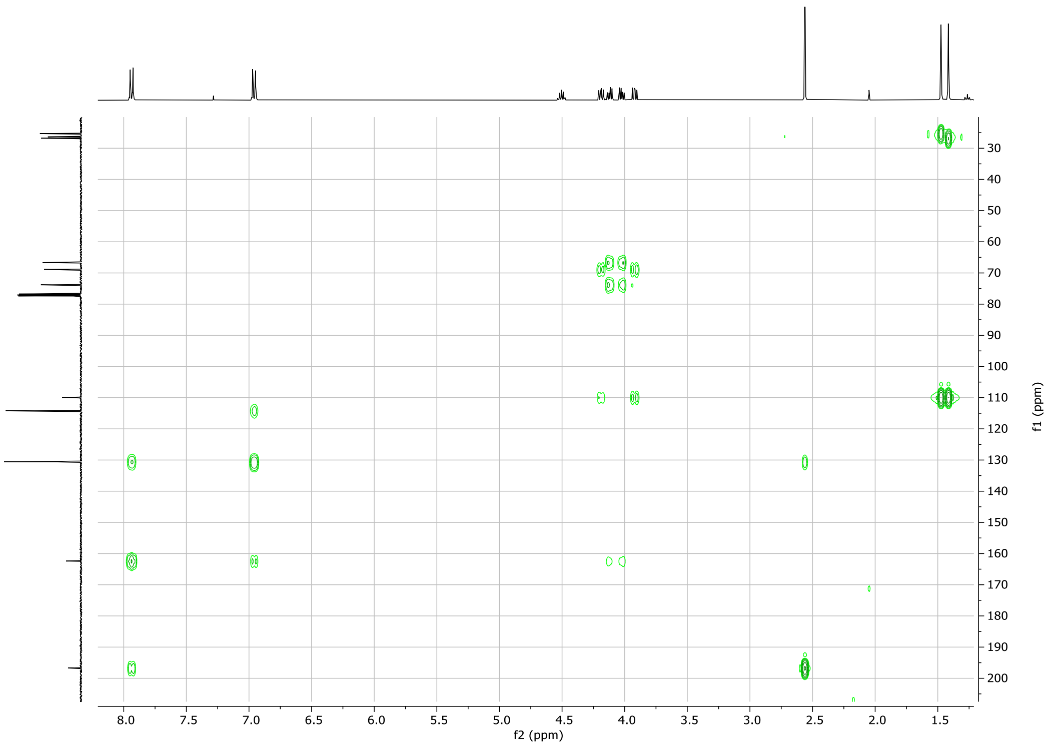

HMBC NMR of **1k** in $CDCl_3$.

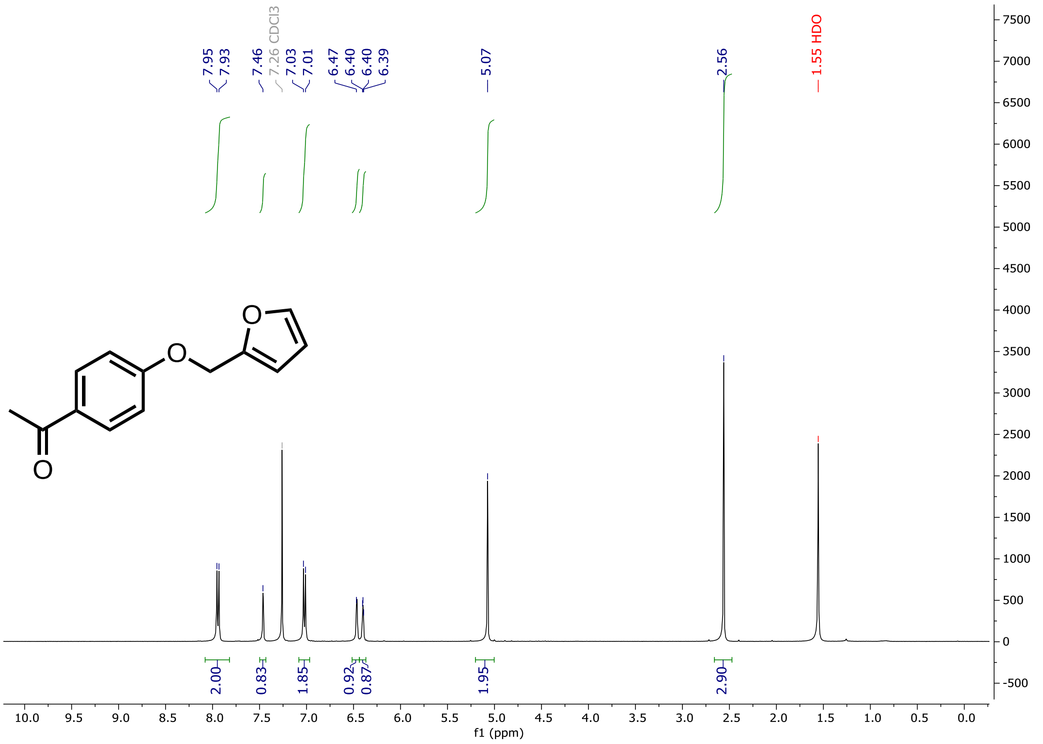

^{13}C NMR of **1l** in $CDCl_3$.

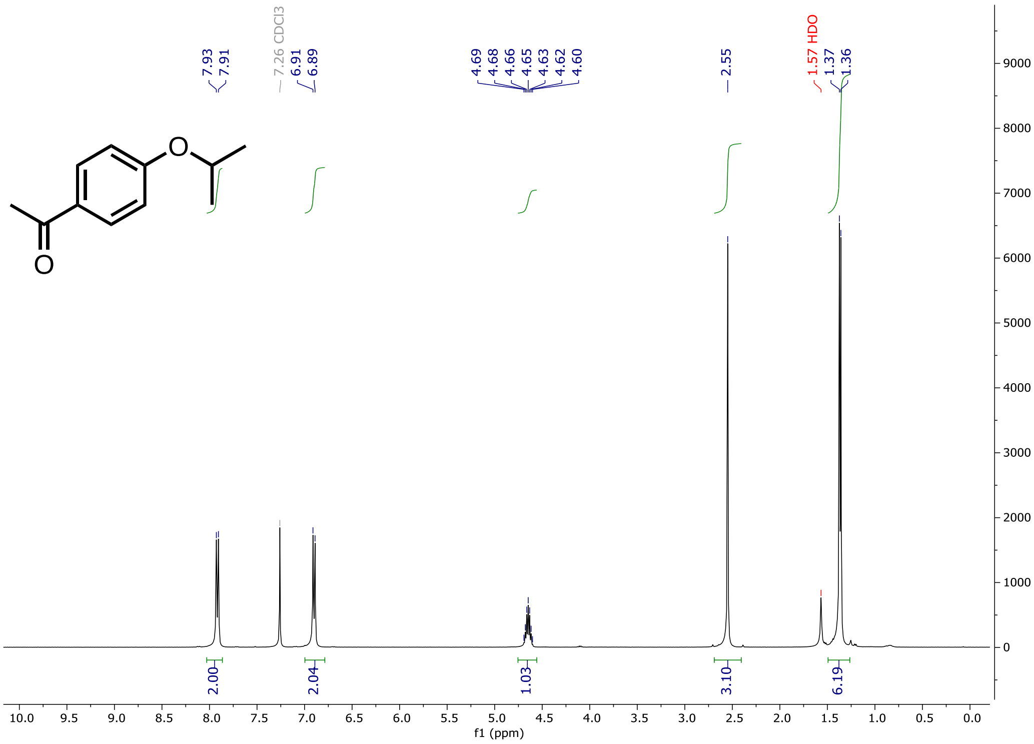

^{1}H NMR of **1m** in $CDCl_3$.

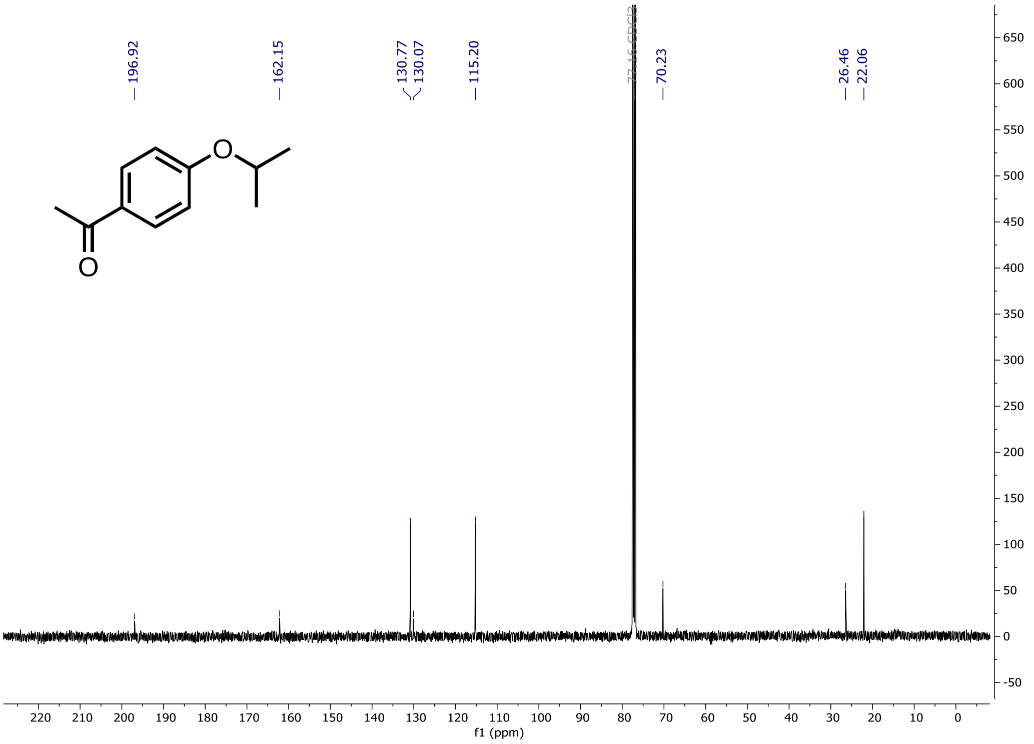

^{13}C NMR of **1m** in $CDCl_3$.

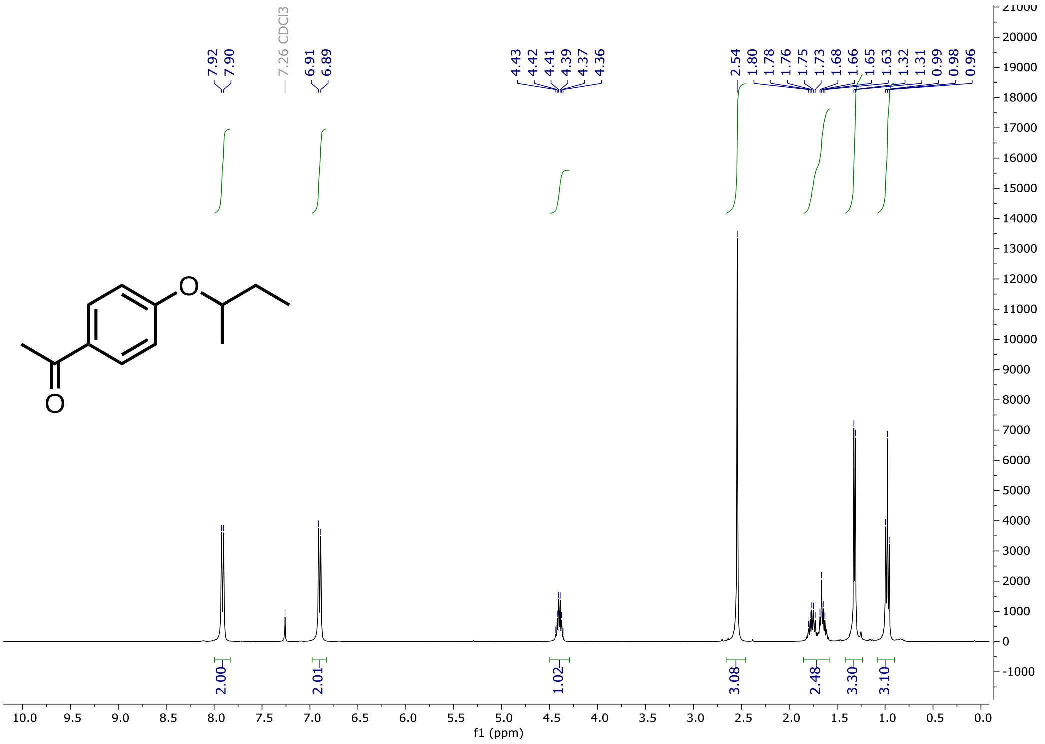

^{1}H NMR of **1n** in $CDCl_3$.

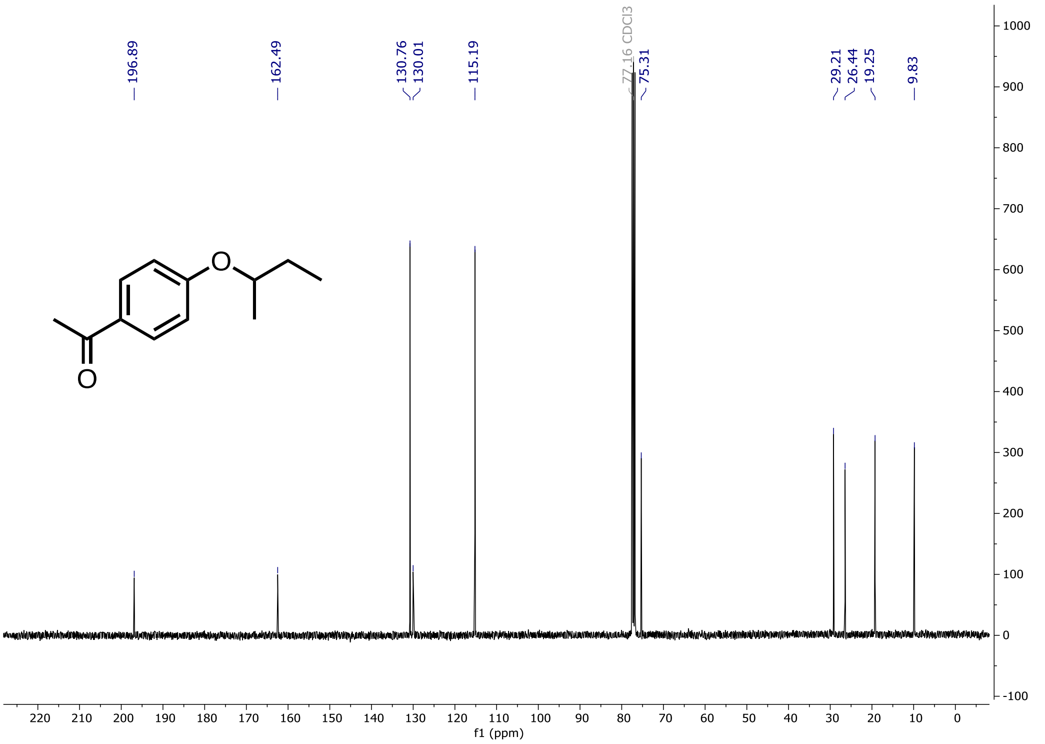

^{13}C NMR of **1n** in $CDCl_3$.

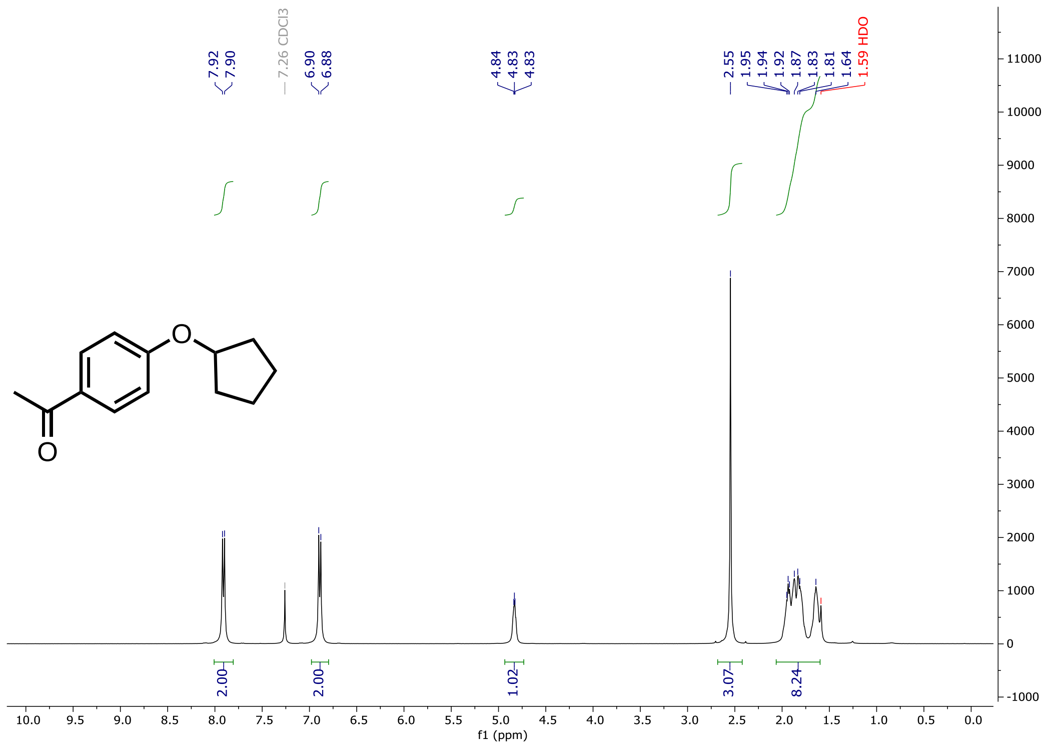

^{1}H NMR of **1o** in $CDCl_3$.

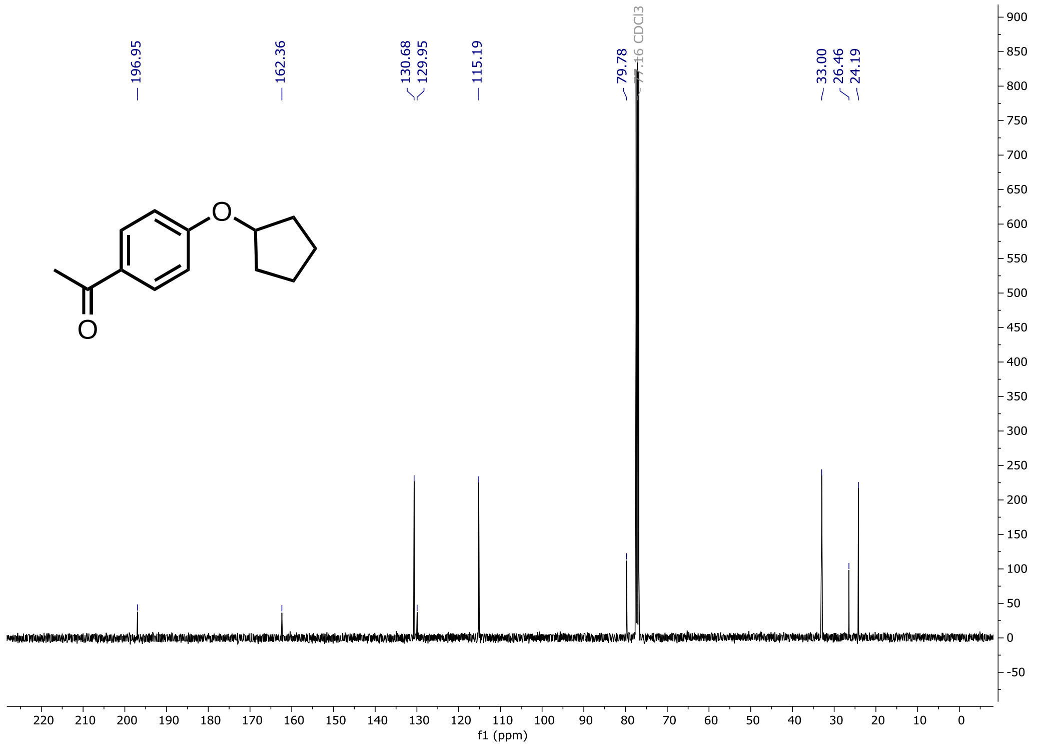

^{13}C NMR of **1o** in $CDCl_3$.

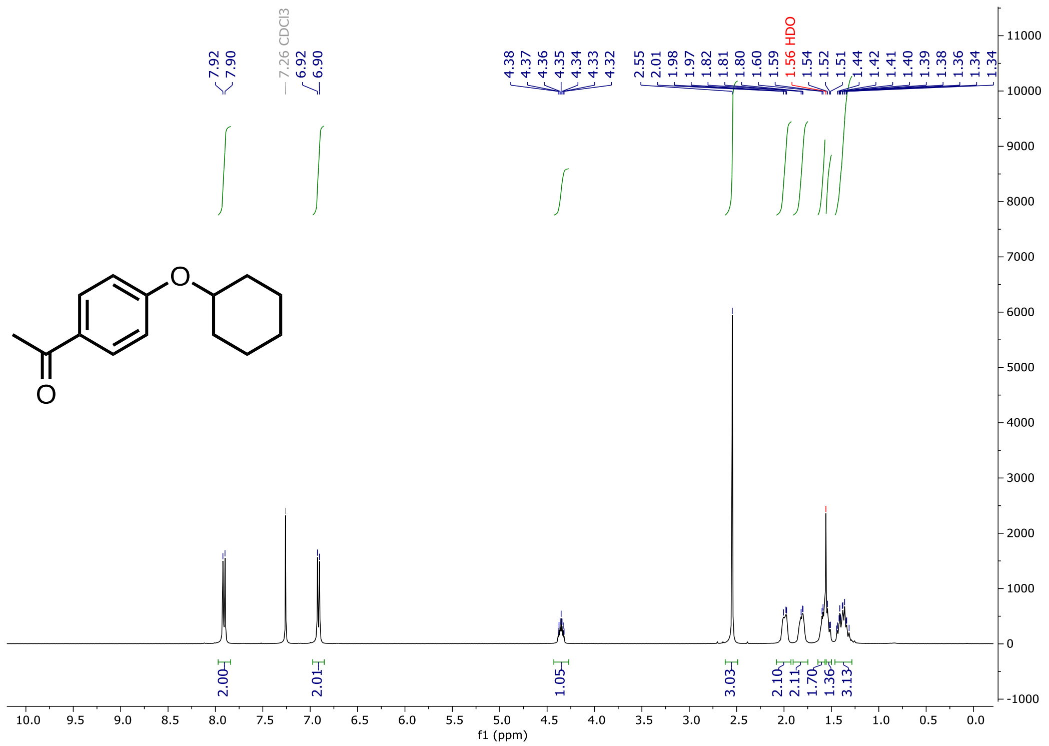

^{1}H NMR of **1p** in $CDCl_3$.

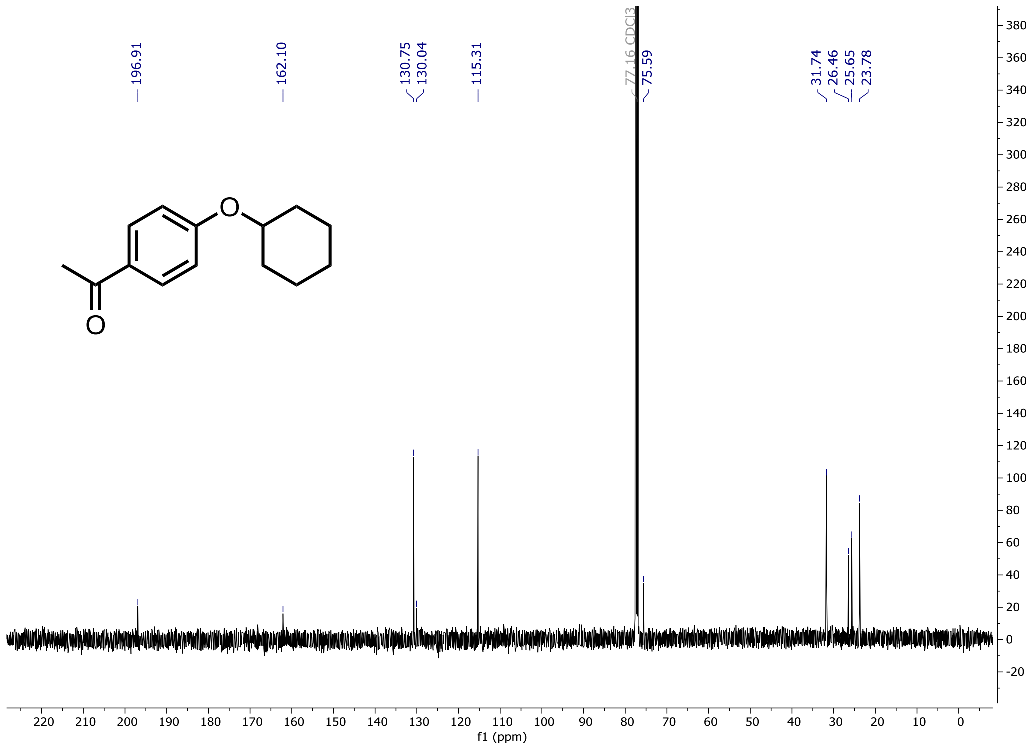

^{13}C NMR of **1p** in $CDCl_3$.

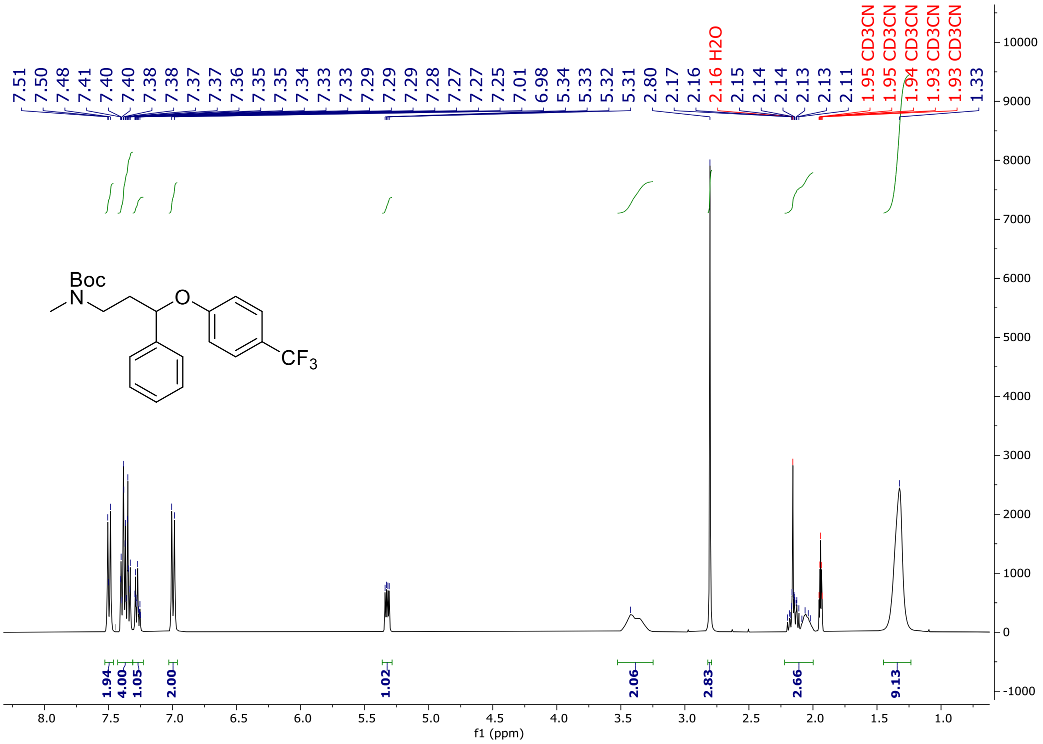

^{1}H NMR of **1q (boc-fluoxetin)** in MeCN-*d3*.

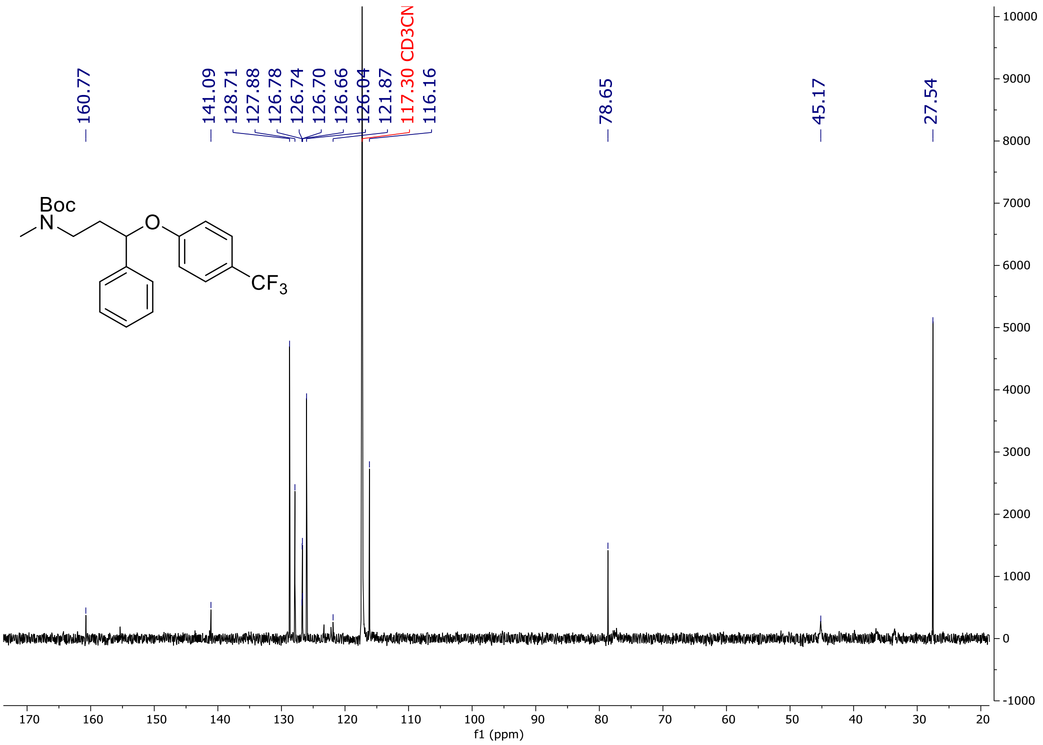

^{13}C NMR of **1q (boc-fluoxetin)** in MeCN-*d3*.

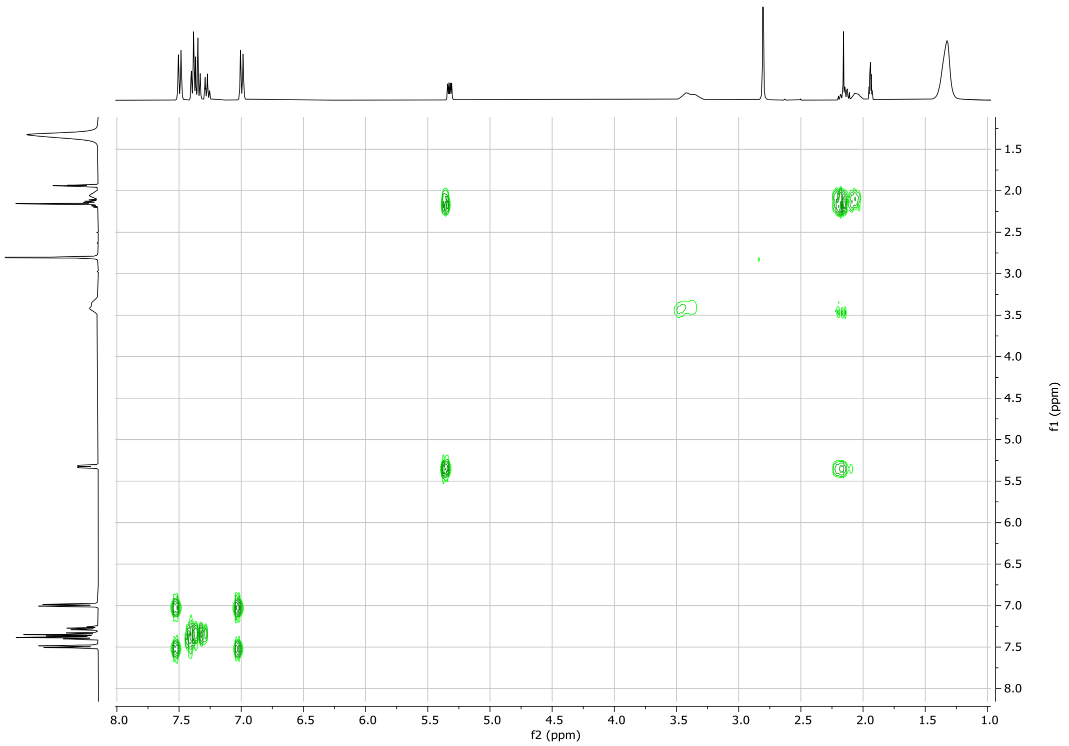

COSY NMR of **1q (boc-fluoxetin)** in MeCN-*d3*.

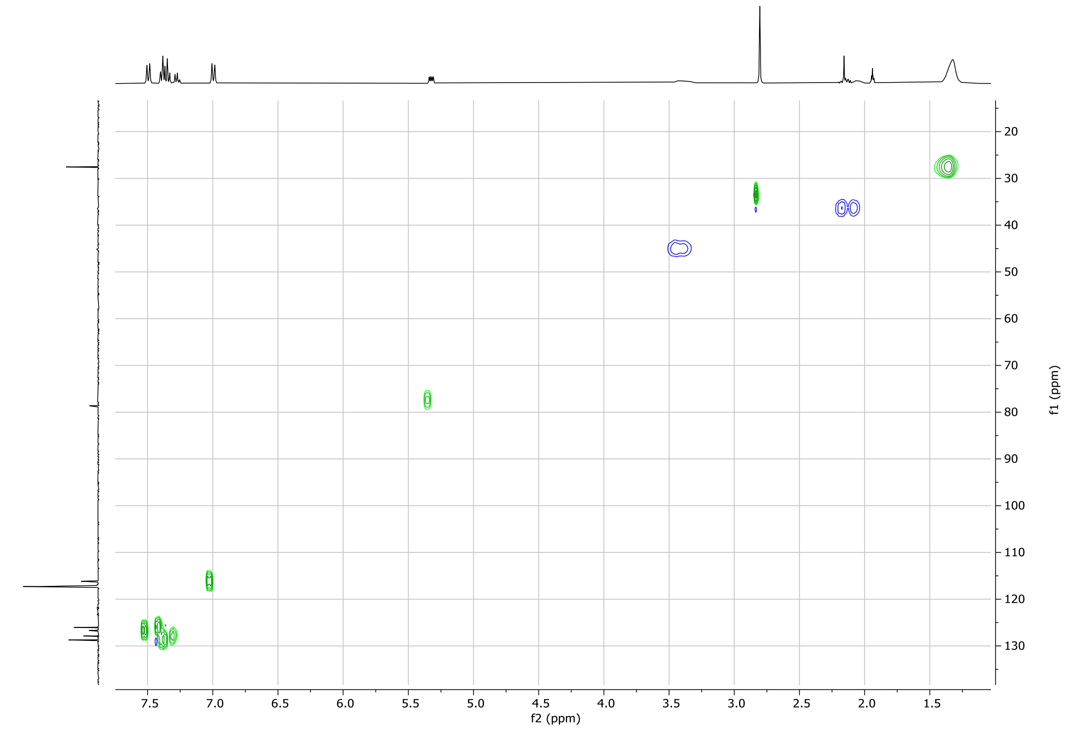

HSQC NMR of **1q (boc-fluoxetin)** in MeCN-*d3*.

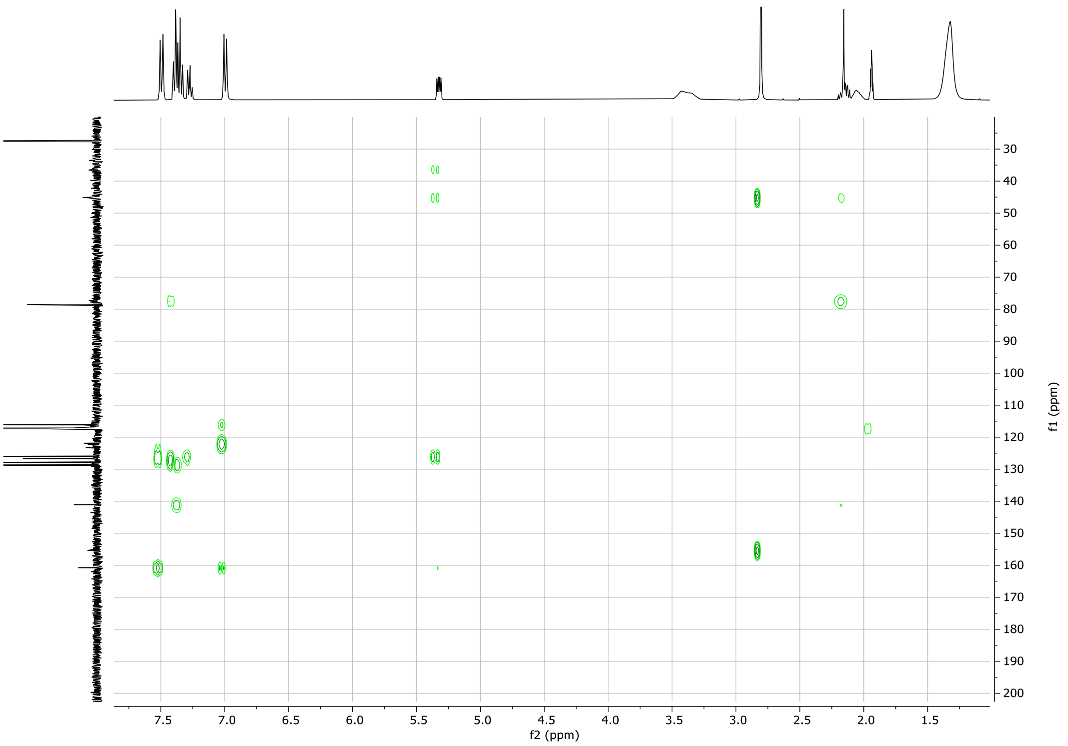

HMBC NMR of **1q (boc-fluoxetin)** in MeCN-*d3*.

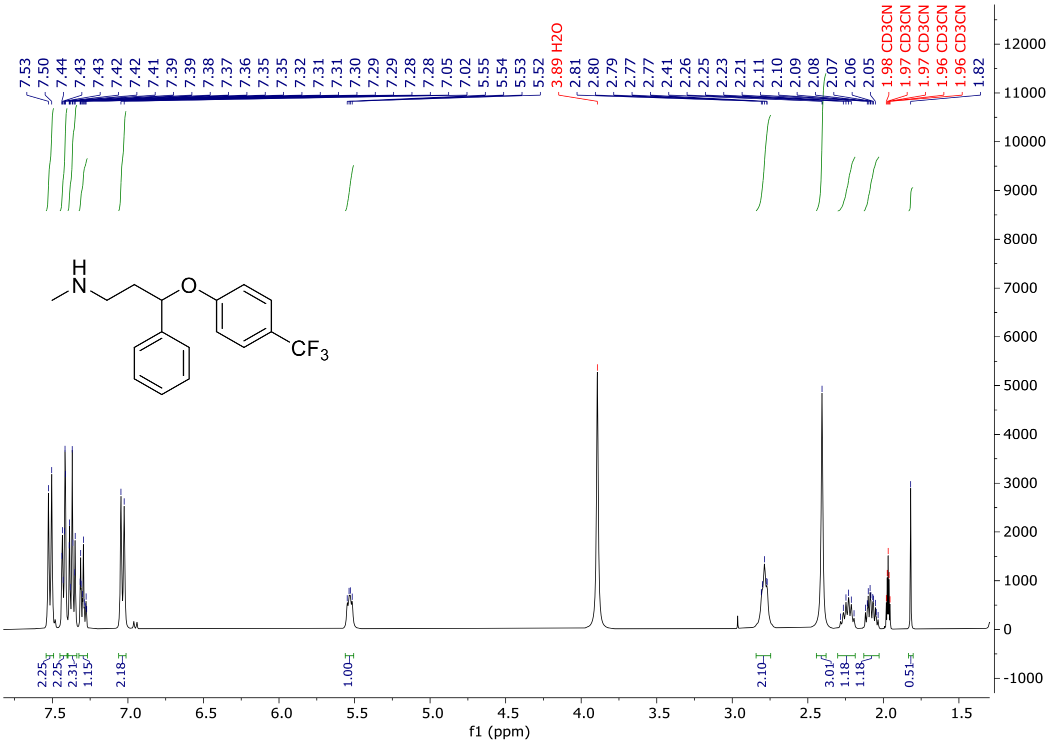

^{1}H NMR of **fluoxetine** (deprotected 1q) in MeCN-*d3*.

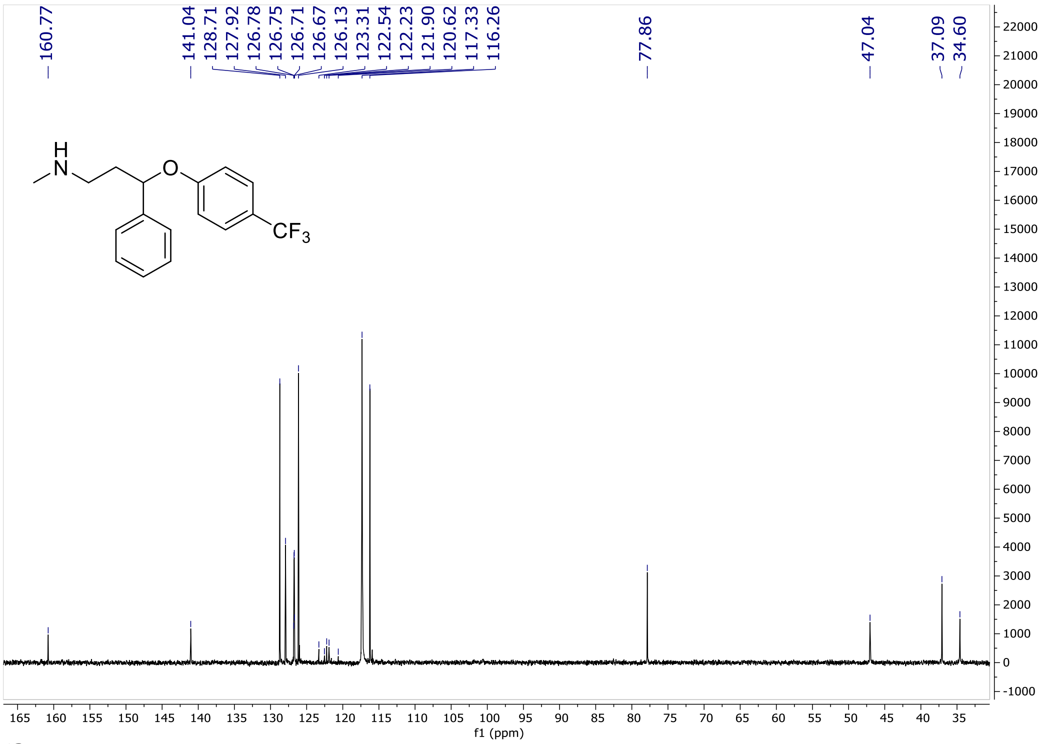

^{13}C NMR of **fluoxetine** (deprotected 1q) in MeCN-*d3*.

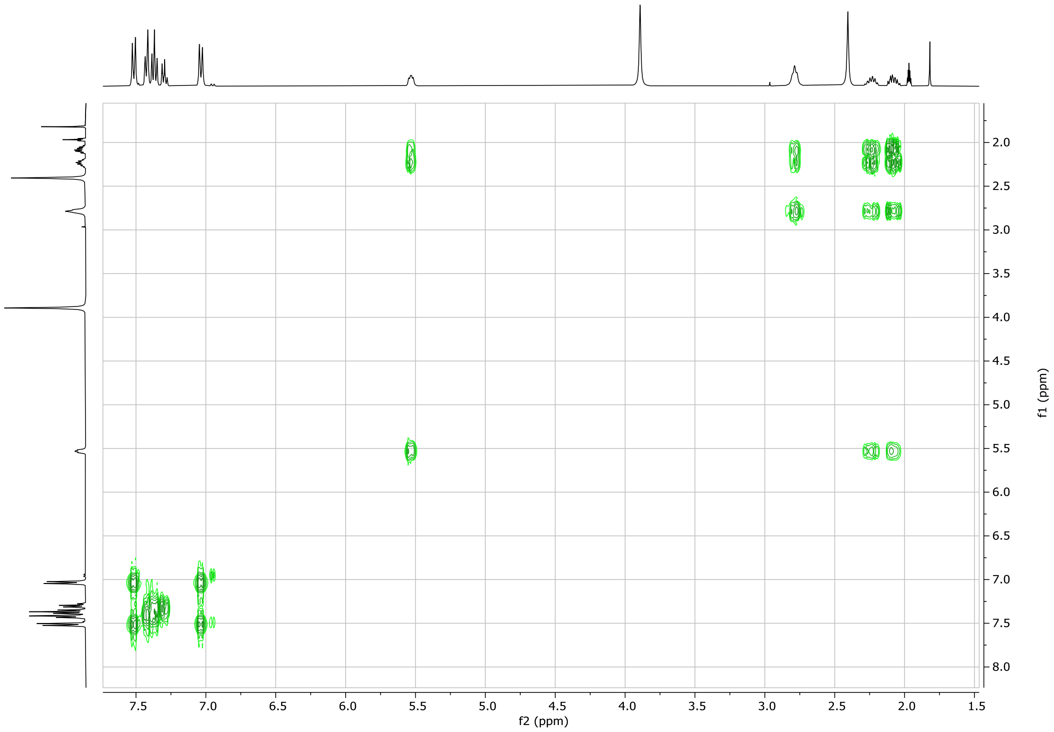

COSY NMR of **fluoxetine** (deprotected 1q) in MeCN-*d3*.

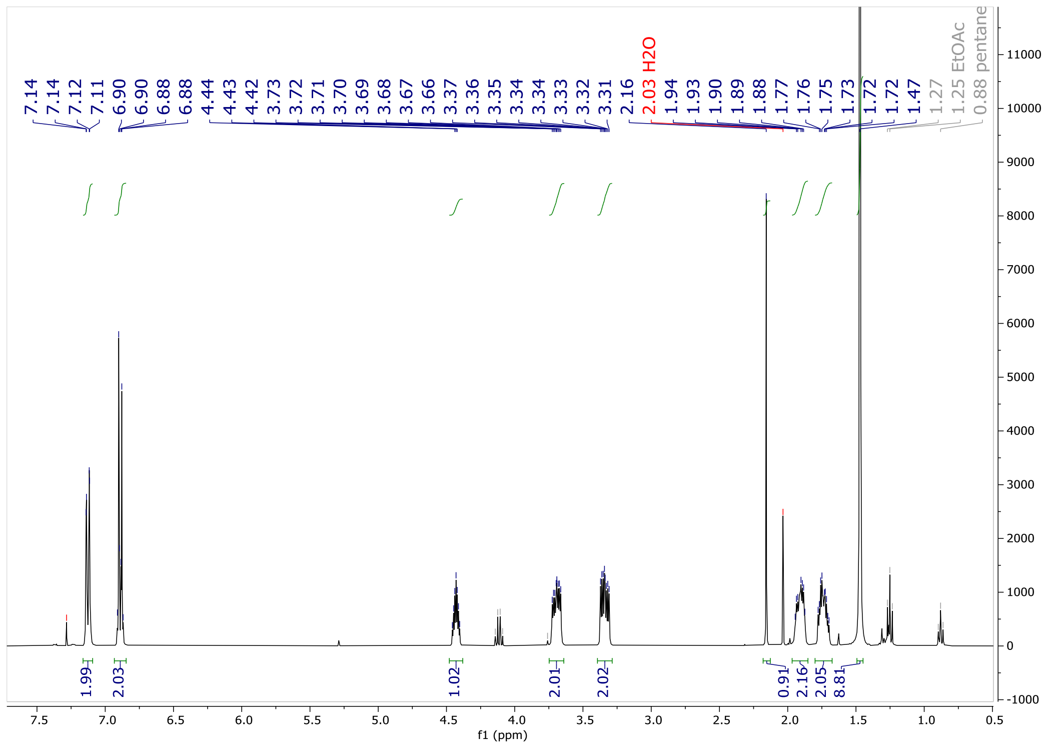

^{1}H NMR of **1r** in $CDCl_3$.

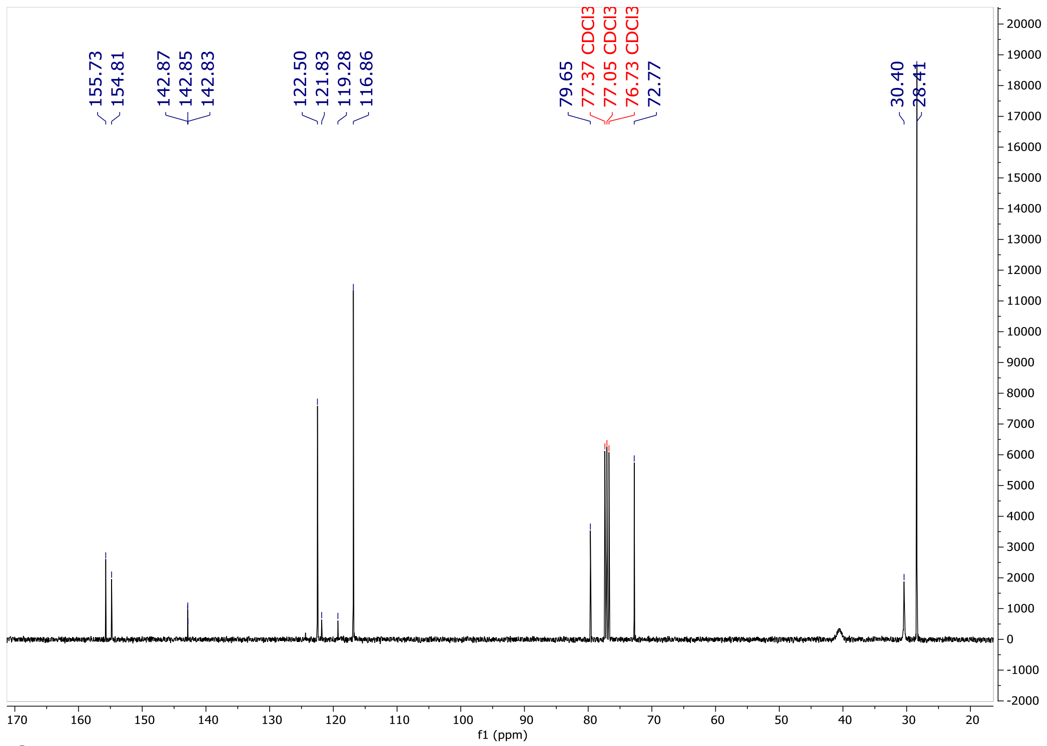

^{13}C NMR of **1r** in $CDCl_3$.

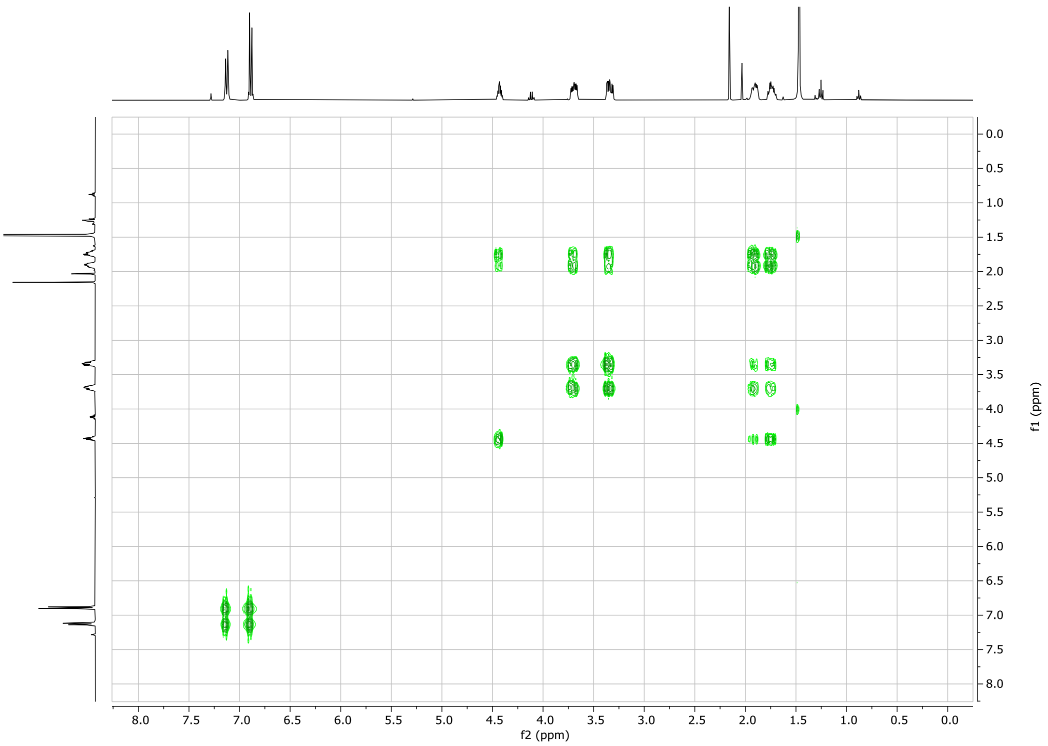

COSY NMR of **1r** in $CDCl_3$.

Transient Absorption (TA) and Time Resolved Infrared (TRIR) data

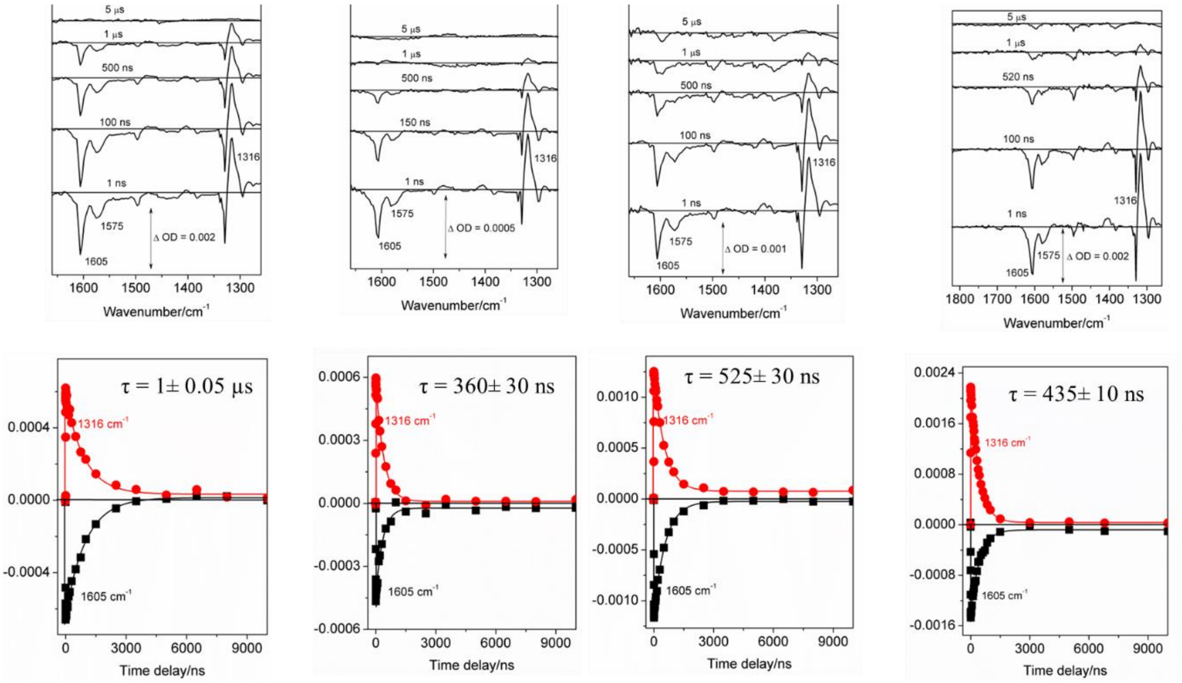

Figure S8. Selected ns-TRIR spectra showing the ^{3}MLCT triplet state of [Ir{dF(CF_3)ppy}$_2$dtbbpy)]PF_6 and its behavior in the presence of reaction components of the modified reaction conditions using TMG at 20 °C. Selected offset ns-TRIR spectra were obtained following 355 nm excitation of (A) 0.1 mM [Ir{dF(CF_3)ppy}$_2$dtbbpy)]PF_6 in CD_3CN solution at 20 oC. (B) TRIR spectra in the presence of 5mM $NiCl_2$-dtbbpy showing quenching of the ^{3}MLCT triplet state of [Ir{dF(CF_3)ppy}$_2$dtbbpy)]PF_6 (C) TRIR spectra in the presence of 10mM TMG showing quenching of the ^{3}MLCT triplet state of [Ir{dF(CF_3)ppy}$_2$dtbbpy)]PF_6. (D) TRIR spectra in the presence of 10nM TMG, 5mM $NiCl_2$-dtbbpy, 10mM 4-bromoacetophenone and 10mM 1-hexanol mixture.

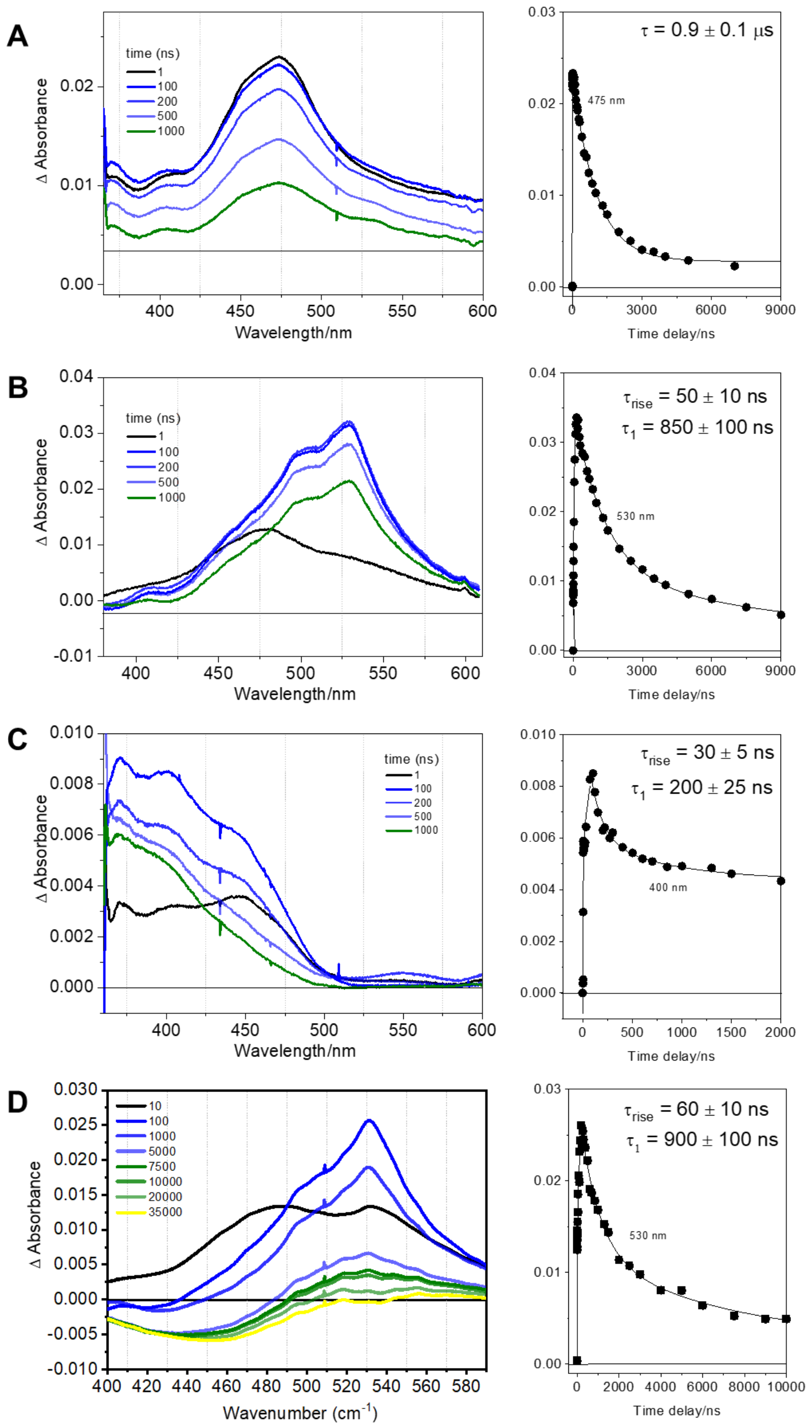

Figure S9. Selected ns-TA spectra showing the ^{3}MLCT excited state of $[Ir\{dF(CF_3)ppy\}_2dtbbpy)]PF_6$ (1mM) (A) and its behavior in the presence of different reaction components following 355 nm excitation. (B) TA spectra in the presence of 10 mM quinuclidine showing quenching of the ^{3}MLCT excited state of $[Ir\{dF(CF_3)ppy\}_2dtbbpy)]PF_6$ and formation of the reduced $[Ir\{dF(CF_3)ppy\}_2dtbbpy)]^{\cdot -}$. (C) TA spectra in the presence of 5 mM $NiCl_2$-dtbbpy showing quenching of the ^{3}MLCT excited state of $[Ir\{dF(CF_3)ppy\}_2dtbbpy)]PF_6$. (D) TA spectra in the presence of the reaction mixture (10 mM 4-bromoacetophenone, 10 mM quinuclidine, 10 mM 1-hexanol, 5 mM $NiCl_2$-dtbbpy) showing that with all reaction components, the system behavior is similar to (B). See Figure 3 to associated TRIR.

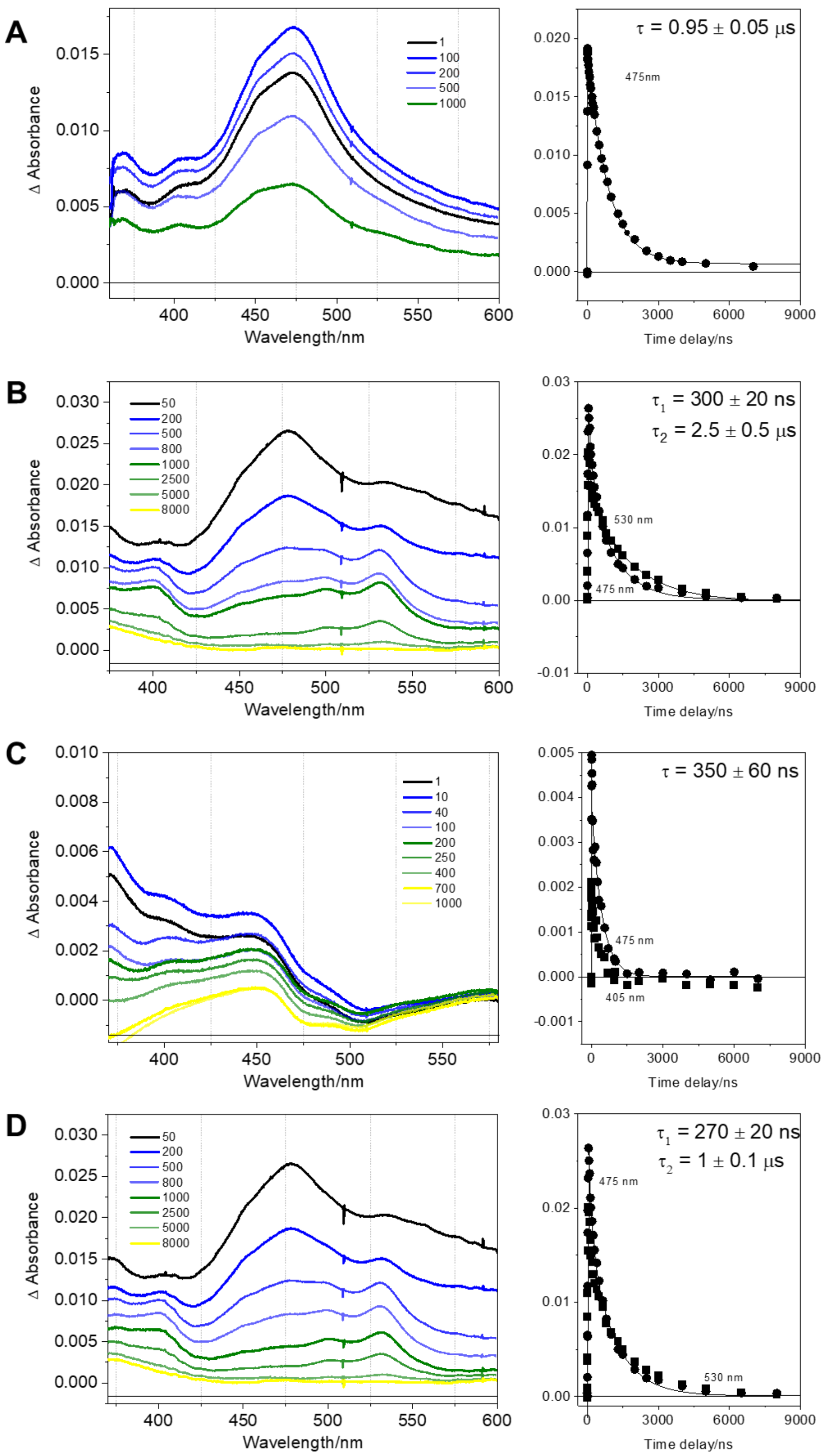

Figure S10. Selected ns-TA spectra showing the ^{3}MLCT triplet state of $[Ir\{dF(CF_3)ppy\}_2dtbbpy)]PF_6$ and its behavior in the presence of reaction components of the modified reaction conditions using TMG at 60 °C. Selected offset ns-TA spectra were obtained following 355 nm excitation of (A) 0.1 mM $[Ir\{dF(CF_3)ppy\}_2dtbbpy)]PF_6$ in CD_3CN solution at 60°C. (B) TA spectra in the presence of 10mM TMG showing quenching of the ^{3}MLCT triplet state of $[Ir\{dF(CF_3)ppy\}_2dtbbpy)]PF_6$. (C) TA spectra in the presence of 5mM $NiCl_2$-dtbbpy showing quenching of the ^{3}MLCT triplet state of $[Ir\{dF(CF_3)ppy\}_2dtbbpy)]PF_6$ and no new detectable transient. (D) TA spectra in the presence of 10nM TMG, 5mM $NiCl_2$-dtbbpy, 10mM 4-bromoacetophenone and 10mM 1-hexanol mixture. See Figure 4 to associated TRIR.
